# Talarolides Revisited: Cyclic Heptapeptides from an Australian Marine Tunicate-Associated Fungus, *Talaromyces* sp. CMB-TU011

**DOI:** 10.3390/md21090487

**Published:** 2023-09-11

**Authors:** Angela A. Salim, Waleed M. Hussein, Pradeep Dewapriya, Huy N. Hoang, Yahao Zhou, Kaumadi Samarasekera, Zeinab G. Khalil, David P. Fairlie, Robert J. Capon

**Affiliations:** 1 Institute for Molecular Bioscience, The University of Queensland, St. Lucia, QLD 4072, Australia; a.salim@uq.edu.au (A.A.S.); w.hussein@uq.edu.au (W.M.H.); p.dewapriya@uq.edu.au (P.D.); h.hoang@imb.uq.edu.au (H.N.H.); yahao.zhou@uq.net.au (Y.Z.); kaumadis@sci.pdn.ac.lk (K.S.); z.khalil@uq.edu.au (Z.G.K.); d.fairlie@imb.uq.edu.au (D.P.F.); 2 ARC Centre of Excellence for Innovations in Peptide and Protein Science, The University of Queensland, St. Lucia, QLD 4072, Australia

**Keywords:** talarolides, *Talaromyces*, cycloheptapeptide, *N*-OH glycine, MATRIX, GNPS molecular networking

## Abstract

Application of a miniaturized 24-well plate system for cultivation profiling (MATRIX) permitted optimization of the cultivation conditions for the marine-derived fungus *Talaromyces* sp. CMB-TU011, facilitating access to the rare cycloheptapeptide talarolide A (**1**) along with three new analogues, B–D (**2**–**4**). Detailed spectroscopic analysis supported by Marfey’s analysis methodology was refined to resolve *N*-Me-l-Ala from *N*-Me-d-Ala, l-*allo*-Ile from l-Ile and l-Leu, and partial and total syntheses of **2**, and permitted unambiguous assignment of structures for **1** (revised) and **2**–**4**. Consideration of diagnostic ROESY correlations for the hydroxamates **1** and **3**–**4**, and a calculated solution structure for **1**, revealed how cross-ring H-bonding to the hydroxamate moiety influences (defines/stabilizes) the cyclic peptide conformation. Such knowledge draws attention to the prospect that hydroxamates may be used as molecular bridges to access new cyclic peptide conformations, offering the prospect of new biological properties, including enhanced oral bioavailability.

## 1. Introduction

During our ongoing investigations into the natural products of Australian marine and terrestrial microbes, we have encountered many new and unusual cyclic and acyclic peptides and depsipeptides, including the antimalarial glyco-cyclohexadepsipeptide-polyketide mollemycin A from a north Queensland marine sediment-derived *Streptomyces* sp. CMB-M0244 [1]; the antitubercular cyclohexapeptide wollamides A–B from a north Queensland desert soil-derived *Streptomyces* sp. MST-115088 [2]; the acyclic peptaibol nonapeptide trichodermamides A–E from a Queensland termite nest-derived fungus *Trichoderma virens* CMB-TN16 [3]; the nitro-depsitetrapeptide-diketopiperazine waspergillamide A from a Queensland mud dauber wasp-derived *Aspergillus* sp. CMB-W031 [4]; the lipocyclopentapeptide scopularides A–H from Queensland mullet gastrointestinal tract-derived *Scopulariopsis* spp. CMB-F458 and CMB-F115, and *Beauvaria* sp. CMB-F585 [5]; and *N*-methylated acyclic undeca- and dodecapeptide talaropeptides A–D [6], and the cycloheptapeptide hydroxamate talarolide A [7], from a Queensland marine tunicate-derived fungus *Talaromyces* sp. CMB-TU011. In the latter case, we took advantage of altering cultivation conditions, with a YES broth cultivation of *Talaromyces* sp. CMB-TU011 yielding the talaropeptides [6] and an M1-saline agar cultivation yielding talarolide A [7].

**Figure 1:**
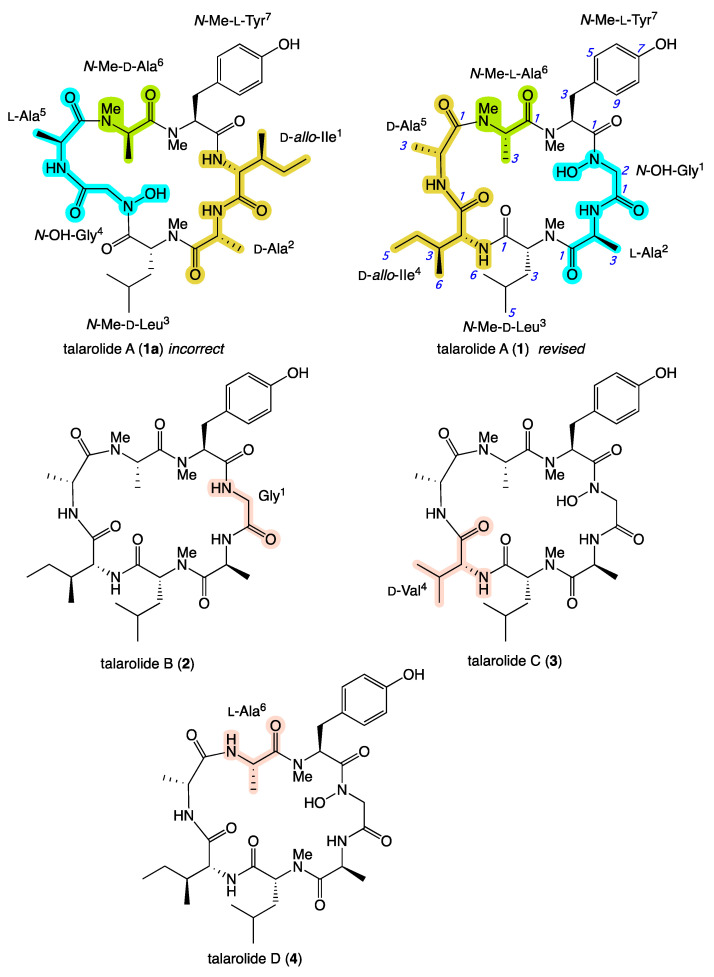
Structures for talarolide A, incorrect (**1a**) [7] and revised (**1**), and new analogues for talarolides B–D (**2**–**4**) from *Talaromyces* sp. CMB-TU011. Highlights (light blue, green, and yellow) show the difference between the incorrect structure (**1a**) and revised structure (**1**) of talarolide A. Pink highlight in structures **2**–**4** shows the amino acid variation compared to **1**.

Notwithstanding that traditional spectroscopic and chemical approaches are generally very effective at assigning structures inclusive of absolute configurations to cyclic peptides, our 2017 account of talarolide A proved challenging, with the proposed structure **1a** inconsistent with a subsequent total synthesis by Brimble et al. [8]. In an effort to address this anomaly, this report describes the application of an innovative miniaturized cultivation profiling methodology (MATRIX) [9] to optimize the production and enable the isolation and characterization of talarolides A–D (**1**–**4**). With access to larger quantities of talarolide A, we were able to secure superior NMR data, which, together with refinements to the Marfey analysis methodology, as well as partial and total syntheses, allowed us to propose a revised structure **1** for talarolide A and to assign structures to the new analogues talarolides B–D (**2**–**4**) as shown (Figure 1).

## 2. Results and Discussion

**Figure 2:**
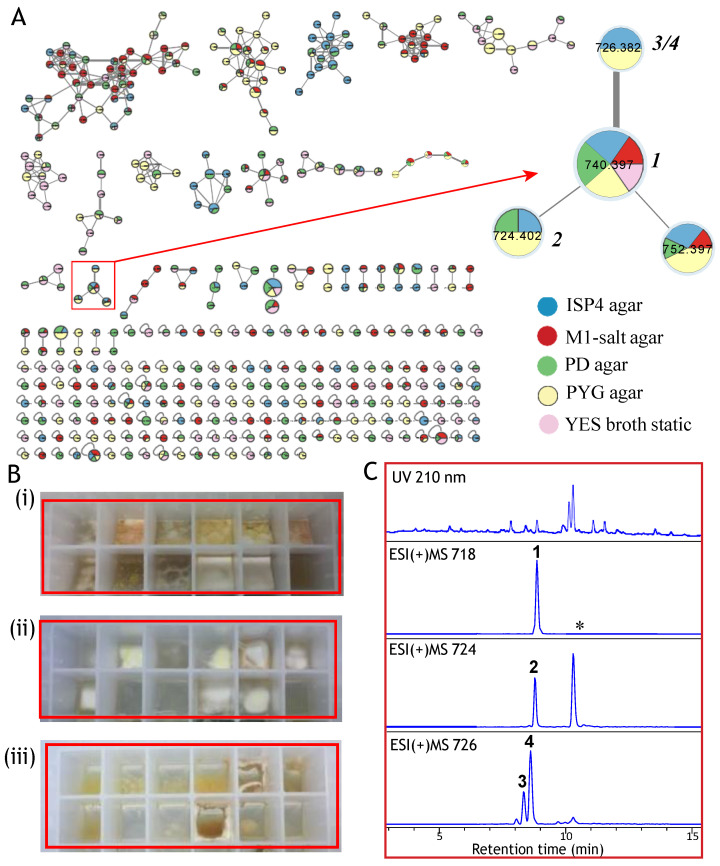
(**A**) GNPS molecular network of *Talaromyces* sp. CMB-TU011 in a selection of five media, with an expansion of the talarolide molecular family. Node segment size correlates with relative yield/metabolite/media; (**B**) images of 24-well plate MATRIX cultivation in 11 different media under three conditions: (i) agar, (ii) static broth, (iii) shaken broth; (**C**) HPLC-DAD-MS chromatograms of CMB-TU011 EtOAc extract obtained from ISP4 agar cultivation, with single ion extractions showing **1**–**4**. (* this peak is not a talarolide analogue).

Since our initial 2017 report on talarolide A [7], we have augmented our microbial biodiscovery efforts by implementing a miniaturized 24-well plate microbioreactor approach to support more comprehensive cultivation profiling (MATRIX) [9], to better optimize production and provide higher yields. Furthermore, we have integrated our MATRIX approach with a chemical profiling strategy employing in situ extraction followed by HPLC-DAD-ESI(+)MS and a UPLC-DAD-QTOF-MS/MS analysis, with the latter visualized as a Global Natural Products Social (GNPS) [10] molecular network, to better detect and prioritize target chemistry (i.e., new from known and rare from common). Applying MATRIX cultivation profiling to *Talaromyces* sp. CMB-TU011 involved 24-well plate cultivations using eleven different media (Table S1) under three conditions (solid agar, and static and shaken broth) (Figure 2B) at 26.5 °C, over 10 days. Following incubation, the resulting 36 individual wells, together with uninoculated media controls, were extracted in situ with EtOAc, and the resulting extracts subjected to chemical profiling. While visualization of the HPLC-DAD-ESIMS data using single ion extraction (SIE, *m*/*z* 718) detected **1** in most extracts, production levels were highly variable with maximum yields observed under M1-salt, ISP-4, PDA and PYG solid agar, and static and shaken broth conditions returning far lower yields (Figure S1). Significantly, a GNPS analysis of the MATRIX extracts revealed a talarolide molecular family (sodiated adducts) incorporating **1** (*m*/*z* 740), and nodes for the deoxy analogue **2** (*m*/*z* 724), lower homologue isomers **3** and **4** (*m*/*z* 726), and an unidentified minor analogue (*m*/*z* 752) (Figure 2A). Based on these analyses, a scaled up (×200 plate) 20-day solid phase ISP-4 agar cultivation of CMB-TU011 was extracted and fractionated by solvent partitioning and gel and reversed phase chromatography, to yield talarolides A–D (**1**–**4**) (Figure 2C and Figure S2). An account of the structure elucidation of **1**–**4** (including structure revision of **1**) is summarized below.

**Figure 3:**
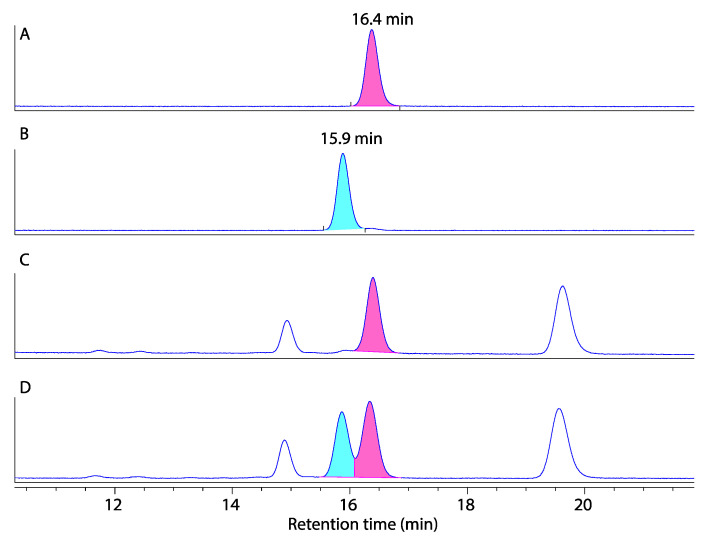
Optimized HPLC conditions for the resolution of Marfey’s derivatives l-FDAA-*N*-Me-l-Ala (pink) and l-FDAA-*N*-Me-d-Ala (light blue). (**A**) synthetic l-FDAA-*N*-Me-l-Ala; (**B**) synthetic l-FDAA-*N*-Me-d-Ala; (**C**) l-FDAA-*N*-Me-l-Ala derived from talarolide A (**1**); (**D**) synthetic l-FDAA-*N*-Me-d-Ala co-injected with l-FDAA-*N*-Me-l-Ala derived from talarolide A (**1**).

**Figure 4:**
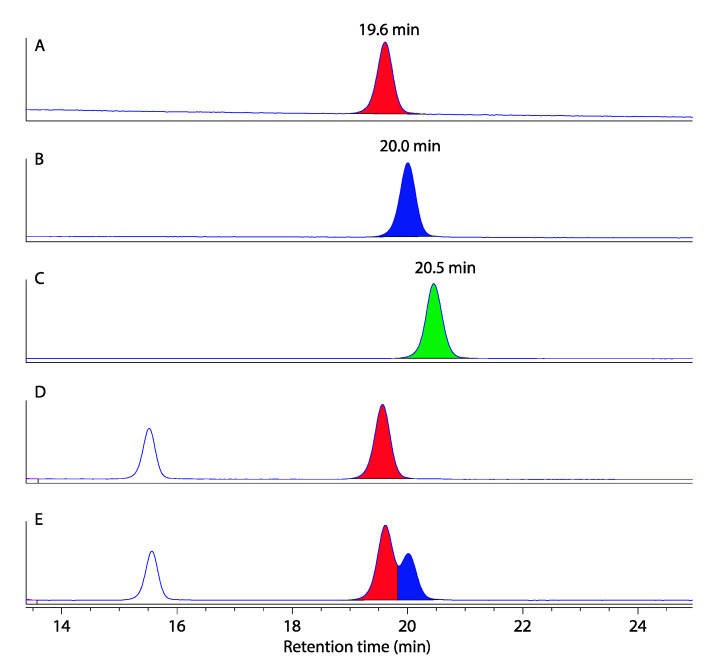
Optimized HPLC conditions for the resolution of Marfey’s derivatives l-FDAA-d-*allo*-Ile (red), l-FDAA-d-Ile (blue) and l-FDAA-d-Leu (green). (**A**) synthetic l-FDAA-d-*allo*-Ile; (**B**) synthetic l-FDAA-d-Ile; (**C**) synthetic l-FDAA-d-Leu, (**D**) l-FDAA-d-*allo*-Ile derived from talarolide A (**1**); (**E**) synthetic l-FDAA-d-Ile co-injected with l-FDAA-d-*allo*-Ile derived from talarolide A (**1**).

**Figure 5:**
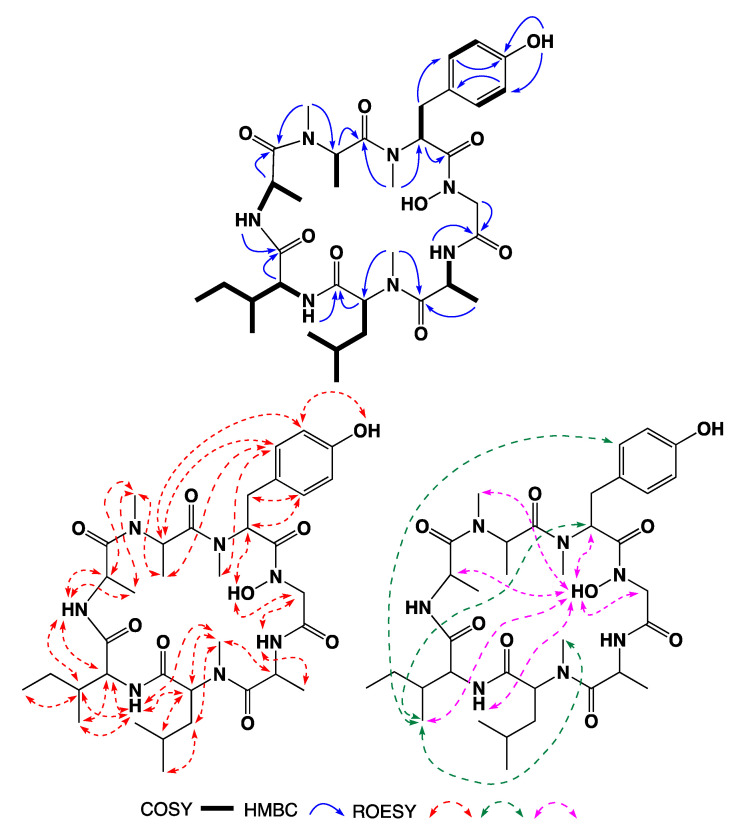
Selected 2D NMR (DMSO-*d*_6_) correlations for talarolide A (**1**).

HRESI(+)MS analysis of **1** revealed a molecular formula (C_35_H_55_N_7_O_9_, Δmmu +2.5) requiring 12 double bond equivalents (DBEs), consistent with our earlier 2017 account of talarolide A [7]. Marfey’s analysis of **1** returned *N*-Me-l-Tyr, d-*allo*-Ile, *N*-Me-d-Leu, l-Ala, d-Ala, and *N*-Me-d-Ala (Figure S34). While this analysis differed from our earlier assessment of talarolide A (i.e., *N*-Me-l-Ala rather than *N*-Me-d-Ala), on revisiting and repeating our earlier analytical HPLC protocols it became apparent that the relative retention times of Marfey’s d-FDAA (or l-FDAA) derivatives of *N*-Me-l-Ala and *N*-Me-d-Ala were very similar, so much so that replicate analyses could experience a reversal in elution times, likely due to subtle variations in eluant composition (i.e., pH) over time. To address this lack of reliability, in this current report, we rely on new analytical HPLC conditions optimized for the unambiguous resolution of Marfey’s derivatives of *N*-Me-l-Ala and *N*-Me-d-Ala (Figure 3 and Figure S38). Likewise, we also developed and applied new, superior analytical HPLC conditions optimized for the differentiation of Marfey’s derivatives of Leu, Ile, and *allo*-Ile (Figure 4 and Figure S39). With the identity and absolute configuration of the amino acid residues in **1** assigned, we next turned our attention to the amino acid sequence. In our earlier structure elucidation of talarolide A, assignment of the planar sequence of amino acid residues relied on an incomplete set of HMBC correlations and interpretation of the MS/MS fragmentation patterns (the latter challenging for cyclic peptides). Fortunately, the re-isolation of **1** enabled the acquisition of superior NMR (DMSO-*d*_6_) data (Table 1, Table 2 and Table S2, and Figure 5 and Figures S3–S8), which allowed for a comprehensive set of HMBC correlations and unambiguous assembly of the amino acid sequence, as shown. To assign the regiochemistry of the l-Ala and d-Ala residues in **1,** we relied on our earlier 2D C_3_ Marfey’s analysis [11] where talarolide A was subjected to partial hydrolysis, derivatization, and chromatographic fractionation to yield the dipeptide d-FDAA-d-*allo*-Ile-d-Ala, with the d-Ala configuration confirmed by a subsequent round of hydrolysis and Marfey’s analysis [7]. Thus, the revised structure for talarolide A (**1**) is as shown. Of particular interest is the unprecedented *N*-OH-Gly residue and its ability to engage in an extensive network of ROESY interactions (and H-bonding) across the cyclic peptide ring (Figure 5, dashed pink), which presumably also facilitates the observed long-range ROESY linkages (Figure 5, dashed green).

**Figure 6:**
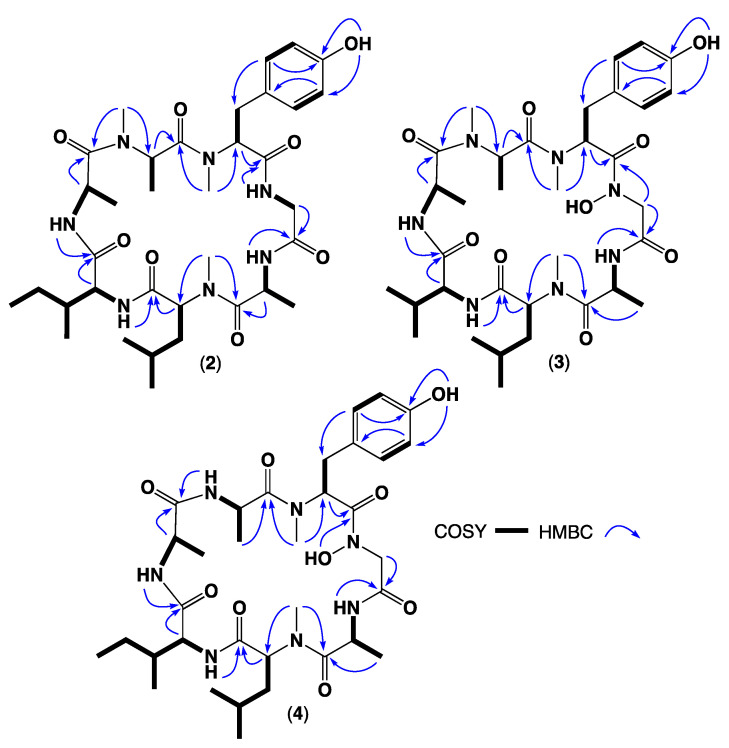
Selected 2D NMR (DMSO-*d*_6_) correlations for talarolides B–D (**2**–**4**).

**Scheme 1:**
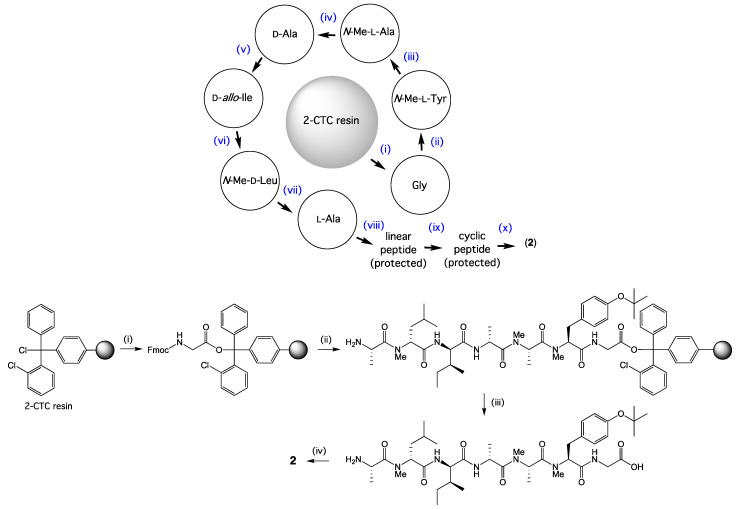
Top: General outline of the solid phase peptide synthesis (SPPS) of talarolide B (**2**). (**i**) Fmoc-Gly-OH coupling to 2-CTC resin, (**ii**–**vii**) sequential peptide chain elongation of Fmoc amino acids, (**viii**) cleavage of linear protected peptide from resin, (**ix**) cyclization of linear protected peptide and (**x**) deprotection to yield **2**. Bottom: Experimental details for SPPS of **2**: (**i**) Fmoc-Gly-OH coupling to 2-CTC resin in the presence of DIPEA (2 h), (**ii**) elongation of peptide sequence through a coupling cycle: Fmoc deprotection with 20% of piperidine in DMF (twice, 5 and 10 min), and a 5 min DMF flow-wash followed by coupling with preactivated Fmoc-amino acid (3.2 eq.) over 2 × 30 min, or 2 × 3 h for coupling of Fmoc-amino acids to sterically hindered *N*-Me-amino acids, (**iii**) cleavage of linear protected peptide from resin using 20% HFIP/DCM (3 × 20 min), (**iv**) cyclization of linear protected peptide using HATU, HOBT, and collidine (14 h), followed by deprotection of *N*Me-l-Tyr using 90% formic acid 40 min to give **2** (16 mg, 23% overall yield).

HRESI(+)MS analysis of **2** revealed a molecular formula (C_35_H_55_N_7_O_8_, Δmmu +0.4) consistent with a deoxy analogue of **1**. Indeed, Marfey’s analysis of **2** returned *N*-Me-l-Tyr, d-*allo*-Ile, *N*-Me-d-Leu, l-Ala, d-Ala, and *N*-Me-d-Ala (Figure S35), while the NMR (DMSO-*d*_6_) data for **2** (Table 1, Table 2 and Table S3, and Figure 6 and Figures S11–S16) revealed chemical shifts and diagnostic correlations that permitted assignment of the same planar amino acid sequence as **1**, where the *N*-OH-Gly in **1** had been replaced by a Gly residue in **2**. Partial hydrolysis of **2** followed by derivatization with l-FDAA followed by UPLC-DAD-MS analysis detected a dipeptide that co-eluted with an authentic synthetic sample of l-FDAA-d-*allo*-Ile-d-Ala, but not synthetic l-FDAA-d-*allo*-Ile-l-Ala (Figure S41), confirming a d-Ala and l-Ala regiochemistry in **2**, common with that independently established for **1.** To further confirm this assignment, we carried out a successful solid phase peptide synthesis of **2** (Scheme 1), with the synthetic sample proving to be identical to natural talarolide B (Figures S50–S52), including co-elution on HPLC (Figure S48).

HRESI(+)MS analysis of 3 revealed a molecular formula (C_34_H_53_N_7_O_9_, Δmmu +3.0) suggestive of a lower homologue (-CH_2_) of 1, with Marfey’s analysis returning *N*-Me-l-Tyr, *N*-Me-d-Ala, d-Ala, l-Ala, d-Val, and *N*-Me-d-Leu (Figure S36). As with 1, the *N*-OH-Gly residue in 3 was not detectable via Marfey’s analysis, although its presence was evident in the NMR (DMSO-*d*_6_) data (Table 1, Table 2 and Table S4, and Figure 4 and Figures S19–S24). Diagnostic 2D NMR correlations (Figure 6) permitted assignment of a planar amino acid sequence comparable to 1, but where the d-*allo*-Ile in 1 was replaced by d-Val in 3. The regiochemistry of the d-Ala and l-Ala residues in 3 was assigned on the basis of biogenetic comparison to 1 and 2, with the structure for talarolide C (3) assigned as shown.

HRESI(+)MS analysis of **4** revealed a molecular formula (C_34_H_53_N_7_O_9_, Δmmu +3.0) suggestive of an alternate lower homologue (-CH_2_) of **1**, with Marfey’s analysis returning *N*-Me-l-Tyr, d-Ala, l-Ala, d-*allo*-Ile, and *N*-Me-d-Leu (Figure S37). As with **1**, the *N*-OH-Gly residue in **4** was not detectable via Marfey’s analysis, although its presence was evident in the NMR (DMSO-*d*_6_) data (Table 1, Table 2 and Table S5, and Figure 6 and Figures S27–S32). Diagnostic 2D NMR correlations revealed a planar amino acid sequence comparable to **1**, but where the *N*-Me-l-Ala in **1** was replaced by an l-Ala in **4**. The regiochemistry of the d-Ala and l-Ala residues in **4** were assigned on the basis of biogenetic comparison to **1** and **2**, with the structure for talarolide D (**4**) assigned as shown.

Of note, both the *N*-OH cyclic peptides **3** and **4** exhibit the same extensive pattern of ROESY correlations (Figures S10, S18 and S26) associated with the *N*-OH moiety evident in **1**, suggesting that all three adopt a common stable conformation dominated by hydrogen bonding to the *N*-OH. Not only is such conformation stabilization not accessible to the cyclic peptide **2**, but the NMR data for **2** reveals two equilibrating conformations (Figure S53), supporting the hypothesis that *N*-hydroxylation can have a pronounced effect on cyclic peptide conformation and stabilization.

**Figure 7:**
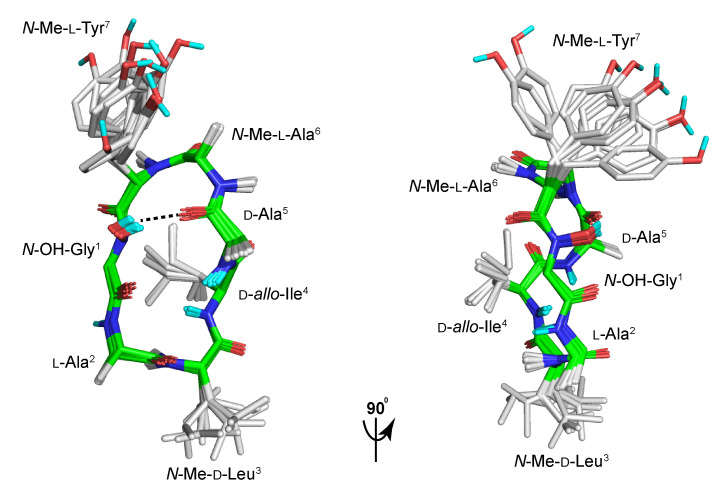
Backbone superimposition of the 10 lowest energy NMR calculated structures for **1** in DMSO-*d*_6_ at 298 K showing hydrogen bonding between *N*-OH-Gly^1^ and the carbonyl in d-Ala^5^ (dashed line) and a *cis*-amide bond between *N*-Me-l-Ala^6^ and *N*-Me-l-Tyr^7^ forming a non-classical alpha turn. The d-*allo*-Ile^4^ amide is projected inward and shielded from solvent, while l-Ala^2^ is solvent exposed. Non-polar hydrogens are omitted for clarity, with backbone carbon atoms (green), sidechain carbon atoms (grey), oxygen atoms (red), nitrogen atoms (blue), and hydrogen atoms (cyan).

**Figure 8:**
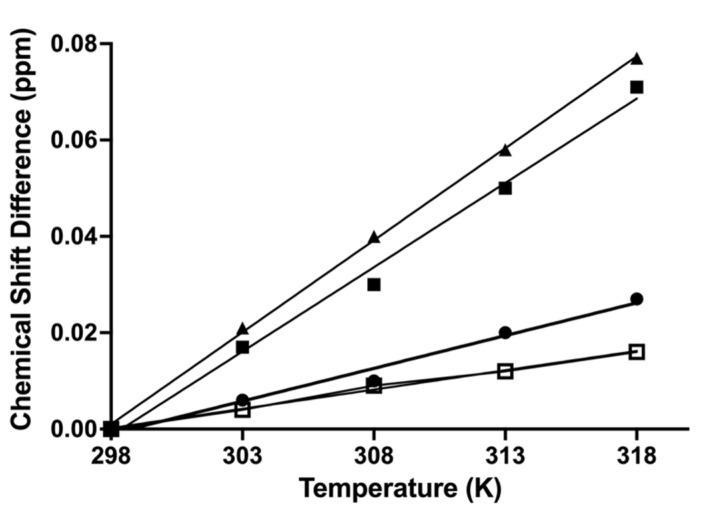
Temperature dependence of the amide NH and OH NMR (DMSO-*d*_6_) chemical shifts for **1**. Line slopes indicating temperature coefficients (Δδ/T) for each residue. Circle: *N*-OH-Gly^1^ (Δδ/T = 1.4 ppb/K); triangle: l-Ala^2^-NH (Δδ/T = 3.9 ppb/K); black square: d-Ala^5^-NH (Δδ/T = 3.5 ppb/K); opened square: d-*allo*-Ile^4^-NH (Δδ/T = 0.3 ppb/K). Small temperature coefficients (Δδ/T) for *N*-OH-Gly^1^ and d-*allo*-Ile^4^-NH indicates hydrogen-bonds or solvent shielded [14].

In an effort to understand this latter phenomenon, we calculated a solution structure for **1** DMSO-*d*_6_ at 298 K using 2D ROESY NMR spectra, calculated from 41 ROE distance restraints, three backbone φ-dihedral angle restraints derived from ^3^*J*_NH-CHα,_ one *cis*-amide between *N*-Me-l-Ala^6^-*N*-Me-l-Tyr^7^, and one hydrogen bond restraint between *N*-OH-Gly^1^ and the d-Ala^5^ carbonyl oxygen (Figure 7). This hydrogen bond restraint was supported by the low temperature coefficient for the *N*-OH-Gly^1^ in variable temperature ^1^H NMR experiments (Figure 8). Structures were calculated in XPLOR-NIH using a dynamic simulated annealing protocol in a geometric force field, and energy minimized using the CHARMM force field [12,13]. The 10 lowest energy structures for talarolide A (**1**) had no distance (≥0.2 Å) or dihedral angle (≥2°) violations and were rigid, convergent structures (average pairwise C_a_ RMSD 0.18 Å) (Figure 7). The structure for **1** supported observations made in the VT (variable temperature) NMR experiments, with the N-OH-Gly^1^ to d-Ala^5^ carbonyl oxygen hydrogen bond and *cis*-amide bond between *N*-Me-l-Ala^6^-*N*-Me-l-Tyr^7^ forming a non-classical alpha turn centered at d-Ala^5^-*N*-Me-l-Ala^6^-*N*-Me-l-Tyr^7^, and with l-Ala^2^ and *N*-Me-d-Leu^3^ forming a distorted beta turn. The d-*allo*-Ile^4^ amide proton projects toward the interior of the structure and is shielded from solvent, while the d-Ala^5^ amide proton is in close proximity to the *N*-OH-Gly^1^ carbonyl oxygen, suggestive of a hydrogen bond and also less accessible to solvent. The opposite side of the molecule features an exposed l-Ala^2^ amide proton, making it more accessible to solvent. From these observations, it can be concluded that the presence of the *N*-OH-Gly provides access to a hydrogen bond that defines the overall conformation of the cyclic peptide. It is intriguing to speculate whether this effect is unique to the talarolide scaffold, with its mix of l and d amino acid residues, or whether it is a more general phenomenon. If the latter, it is possible that *N*-hydroxylation could prove to be a valuable molecular tool for accessing new peptide chemical space.

## 3. Materials and Methods

### 3.1. General Experimental Procedures

Chiroptical measurements ([α]_D_) were obtained on a JASCO P-1010 polarimeter in a 100 × 2 mm cell at 25 °C. Nuclear magnetic resonance (NMR) spectra were acquired on a Bruker Avance 600 MHz spectrometer with either a 5 mm PASEL ^1^H/D-^13^C Z-Gradient probe or 5 mm CPTCI ^1^H/^19^F-^13^C/^15^N/DZ-Gradient cryoprobe. The spectra were acquired at 25 °C in DMSO-*d*_6_ and referenced to residual signals (δ_H_ 2.50 and δ_C_ 39.5 ppm) in deuterated solvents. High-resolution ESIMS measurements were obtained on a Bruker micrOTOF mass spectrometer by direct infusion in MeCN at 3 μL/min using sodium formate clusters as an internal calibrant. UPLC-QTOF analysis was performed on a UPLC-QTOF instrument comprising an Agilent 1290 Infinity II UPLC (Agilent Zorbax C_8_ RRHD 1.8 μm, 2.1 × 250 mm column, eluting at 0.417 mL/min with a 2.50 min gradient elution from 90% H_2_O/MeCN to 100% MeCN with a constant 0.1% formic acid modifier) coupled to an Agilent 6545 QTOF mass detector (Agilent, Mulgrave, Australia). Liquid chromatography-diode array-mass spectrometry (HPLC-DAD-MS) data were acquired on an Agilent 1260 series separation module equipped with an Agilent G6125B series single quad mass detector and diode array detector (Agilent Poroshell 120 SB-C_8_ 2.7 μm, 3.0 × 150 mm column, eluting at 0.8 mL/min with a 6.25 min gradient elution from 90% H_2_O/MeCN to 100% MeCN with a constant 0.05% formic acid modifier). Ultra-high performance liquid chromatograms (UPLCs) were obtained on an Agilent 1290 infinity UPLC system composed of a 1290 infinity binary pump, thermostat, autosampler, and diode array detector (Agilent, Mulgrave, AustraliaPreparative and semi-preparative HPLC were performed using an Agilent 1100 Series diode array and/or multiple wavelength detectors and an Agilent 1100 Series fraction collector (Agilent, Mulgrave, Australia). *N*α-(2,4-dinitro-5-fluorophenyl)-l-alaninamide (l-FDAA, synonym 1-fluoro-2-4-dinitrophenyl-5-l-alanine amide) and *N*α-(2,4-dinitro-5-fluorophenyl)-d-alaninamide (d-FDAA, synonym 1-fluoro-2-4-dinitrophenyl-5-d-alanine amide) were purchased from Merck (Darmstadt, Germany). Amino acids and standards were purchased from BAChem (Torrance, CA, USA) or Merck (Darmstadt, Germany). Analytical-grade solvents were used for solvent extractions. Chromatography solvents were of HPLC grade supplied by Labscan (Bangkok, Thailand) or Merck (Darmstadt, Germany) and filtered/degassed through 0.45 μm polytetrafluoroethylene (PTFE) membrane prior to use. Deuterated solvents were purchased from Cambridge Isotopes (Tewksbury, MA, USA). Microorganisms were manipulated under sterile conditions using a Laftech class II biological safety cabinet and incubated in either MMM Friocell incubators (Lomb Scientific, Taren Point, NSW, Australia) or an Innova 42R incubator shaker (John Morris, Chatswood, NSW, Australia).

### 3.2. Collection and Taxonomy of Talaromyces sp. CMB-TU011

The isolation and taxonomy of *Talaromyces* sp. CMB-TU011 from an unidentified tunicate collected from Tweed Heads, NSW, Australia, has been previously reported [7].

### 3.3. Cultivation and Fractionation of Talaromyces sp. CMB-TU011

A loop of spores from a 7-day old M1-salt culture of CMB-TU011 was streaked on ISP-4 agar plates (×200) and incubated for 20 days at 26.5 °C, after which the combined agar/mycelia was extracted with EtOAc (3 × 500 mL) and concentrated in vacuo to yield an extract (264 mg), which was partitioned between n-hexane and aqueous MeOH to give hexane (70 mg) and MeOH (194 mg) soluble fractions. The MeOH fraction was subjected to gel chromatography (Sephadex^®^ LH-20 (Merck, Darmstadt, Germany) in MeOH) to obtain 15 fractions, which were combined based on HPLC-DAD-MS analysis to yield a talarolides-enriched fraction (30.5 mg). Further semi-preparative HPLC (Agilent Zorbax Eclipse C_8_ column, 5 µm, 9.4 × 250 mm, 32% MeCN/H_2_O isocratic elution at 3.0 mL/min inclusive of an isocratic 0.01% TFA/MeCN modifier) was used to yield talarolide A (**1**) (*t*_R_ 25.6 min, 3.4 mg, 1.3%), talarolide B (**2**) (*t*_R_ 21.3 min, 1.1 mg, 0.42%), talarolide C (**3**) (*t*_R_ 16.1 min, 1.1 mg, 0.42%), and talarolide D (**4**) (*t*_R_ 19.3 min, 0.9 mg, 0.34%). (Note: % yields are calculated as a weight to weight of the EtOAc extract) (Figure S2).

Talarolide A (**1**): white powder; [α]_D_^25^ –17 (c 0.12, MeOH); 1D and 2D NMR (DMSO-*d*_6_) see Table 1, Table 2 and Table S2, and Figures S3–S8; UPLC-QTOF (MS/MS) fragmentation see Figure S42; HRESI(+)MS *m*/*z* 740.3978 [M + Na]^+^ (calcd for C_35_H_55_N_7_O_9_Na 740.3953) (Figure S9).

Talarolide B (**2**): colorless amorphous solid; [α]_D_^25^ –15 (c 0.059, MeOH); NMR (DMSO-*d*_6_) see Table 1, Table 2 and Table S3, and Figures S10–S16; UPLC-QTOF (MS/MS) fragmentation see Figure S43; HRESI(+)MS *m*/*z* 724.4008 [M + Na]^+^ (calcd for C_35_H_55_N_7_O_8_Na 724.4004) (Figure S17).

Talarolide C (**3**): colorless amorphous solid; [α]_D_^25^ –15 (c 0.072, MeOH); NMR (DMSO-*d*_6_) see Table 1, Table 2 and Table S4, and Figures S18–S25; UPLC-QTOF (MS/MS) fragmentation see Figure S44; HRESI(+)MS *m*/*z* 726.3827 [M + Na]^+^ (calcd for C_34_H_53_N_7_O_9_Na 726.3797) (Figure S25).

Talarolide D (**4**): colorless amorphous solid; [α]_D_^25^ –14 (c 0.054, MeOH); NMR (DMSO-*d*_6_) see Table 1, Table 2 and Table S5, and Figures S26–S32; UPLC-QTOF (MS/MS) fragmentation see Figure S45; HRESI(+)MS *m*/*z* 726.3827 [M + Na]^+^ (calcd for C_34_H_53_N_7_O_9_Na 726.3797) (Figure S33).

### 3.4. Marfey’s Analysis of Talarolides A–D

#### 3.4.1. Standard Marfey’s Hydrolysis and Derivatization Method #1

A sample analyte (50 μg) in 6 M HCl (100 μL) was heated to 100 °C in a sealed vial for 12 h, after which the hydrolysate was concentrated to dryness at 40 °C under a stream of dry N_2_. The hydrolysate was then treated with 1 M NaHCO_3_ (20 μL) and l-FDAA (1-fluoro-2,4-dinitrophenyl-5-l-alanine amide) or d-FDAA (1-fluoro-2,4-dinitrophenyl-5-d-alanine amide) as a 1% (*w*/*v*) solution in acetone (40 μL) at 40 °C for 1 h, after which the reaction was neutralized with 1 M HCl (20 μL), diluted with MeCN (200 μL) and filtered (0.45 μm PTFE) prior to analysis.

#### 3.4.2. Standard Marfey’s HPLC Method #2

An aliquot of Marfey’s derivatized analyte (3 μL) (see method #1) was subjected to HPLC-DAD-MS analysis using a binary solvent system (Phase A: 95% H_2_O: 5% MeCN: 0.1% formic acid; Phase B: 95% MeOH: 5% MeCN: 0.1% formic acid) on an Agilent Poroshell 120 SB-C_8_ 2.7 μm, 3.0 × 150 mm column, at 50 °C with a 0.8 mL/min linear gradient over 29 min from 16% to 63% Phase B in A, and with DAD (340 nm) and ESI(±)MS monitoring, supported by single ion extraction (SIE) methodology, and with comparison to authentic standards of Marfey’s derivatized amino acids.

#### 3.4.3. Marfey’s HPLC Method #3 Optimized for Resolving N-Me-Ala Derivatives

An aliquot of Marfey’s derivatized analyte (3 μL) (see method #1) was subjected to UPLC-DAD-MS analysis using the same binary solvent system and detection as described above (method #2), but with an isocratic 0.8 mL/min elution at 23% Phase B in A using an Agilent Poroshell 120 EC-C_18_ 2.7 μm, 3.0 × 150 mm column at 50 °, with comparison to authentic standards of Marfey’s derivatized amino acids (Figure 3 and Figure S38).

#### 3.4.4. Marfey’s HPLC Method #4 Optimized for Resolving Leu, Ile and *allo*-Ile Derivatives

An aliquot of Marfey’s derivatized analyte (3 μL) (see method #1) was subjected to UPLC-DAD-MS analysis using the same binary solvent system and detection as described above (method #2), but with an isocratic 0.8 mL/min elution at 37% Phase B in A, with comparison to authentic standards of Marfey’s derivatized amino acids (Figure 4 and Figure S39).

#### 3.4.5. Marfey’s Analysis of Talarolides A–D (**1**–**4**)

Samples of talarolides A–D (**1**–**4**) (50 μg) were subjected to standard Marfey’s hydrolysis and derivatization (method #1), after which individual aliquots of Marfey’s derivatized analytes (3 μL) were subjected to each of methods #2, #3, and #4 to unambiguously identify the following amino acid constituents:

Talarolide A (**1**): l-Ala, *N*-Me-d-Leu, d-*allo*-Ile, d-Ala, *N*-Me-l-Ala, *N*-Me-l-Tyr (see Figure 3, Figure 4 and Figure S34)

Talarolide B (**2**): Gly, l-Ala, *N*-Me-d-Leu, d-*allo*-Ile, d-Ala, *N*-Me-l-Ala, *N*-Me-l-Tyr (see Figure S35)

Talarolide C (**3**): l-Ala, *N*-Me-d-Leu, d-Val, d-Ala, *N*-Me-l-Ala, *N*-Me-l-Tyr (see Figure S36)

Talarolide D (**4**): l-Ala, *N*-Me-d-Leu, d-*allo*-Ile, d-Ala, l-Ala, *N*-Me-l-Tyr (see Figure S37)

### 3.5. Two-Dimensional Marfey’s Analysis of Talarolide B

#### 3.5.1. Two-Dimensional Marfey’s Method #5 Partial Hydrolysis of Talarolide B

A sample of talarolide B (**2**) (50 μg) was subjected to the standard Marfey’s hydrolysis conditions (method #1) but with a reduced reaction time from 12 to 3 h, and after derivatization yielded a dipeptide attributed to l-FDAA-d-*allo*-Ile-Ala (unspecified Ala configuration) based on an HPLC-DAD-MS (Marfey’s method #2) [*t*_R_ 22.3 min, *m*/*z* 455 (M + H)] (Figure S40).

#### 3.5.2. Synthesis of Dipeptides l-FDAA-d-*allo*-Ile-l-Ala and l-FDAA-d-*allo*-Ile-d-Ala

Syntheses were performed using standard peptide synthesis on a 2-chlorotrityl chloride (2-CTC) resin (substitution ratio: 1.55 mmol/g, 0.1 mmol scale, 64.5 mg) using hexafluorophosphate azabenzotriazole tetramethyl uranium (HATU) and *N,N*-diisopropylethylamine (DIPEA) coupling, and fluorenylmethoxycarbonyl (Fmoc) protection chemistry.

#### 3.5.3. Coupling of the First Amino Acid

After swelling the 2-CTC resin for 20 min in dry CH_2_Cl_2_ (2 mL), a solution of either Fmoc-l-Ala-OH or Fmoc-d-Ala-OH (1.2 eq.) and DIPEA (44 µL, 0.25 mmol, 2.5 eq.) in dry CH_2_Cl_2_ (2 mL) was added to the resin and mixed for 2 h. The resin was filtered and MeOH (200 µL) was added and mixed for 15 min to cap the resin. The resin was washed with dry CH_2_Cl_2_ (5 × 1 min), 1:1 CH_2_Cl_2_/MeOH (5 × 1 min) and MeOH (2 × 1 min).

#### 3.5.4. Coupling of Fmoc-d-*allo*-Ile

Coupling of Fmoc-d-*allo*-Ile was achieved by dissolving Fmoc-d-*allo*-Ile (0.32 mmol, 3.2 eq.), in 0.4 M HATU/DMF (0.75 mL, 0.3 mmol, 3.0 eq.), followed by the addition of DIPEA (105 µL, 0.6 mmol, 6.0 eq.). The coupling cycle consisted of Fmoc deprotection with 20% of piperidine in DMF (twice, 5 and 10 min), a 5 min DMF flow-wash, followed by coupling with preactivated Fmoc-D-*allo*-Ile (3.2 eq.) over 2 × 30 min.

#### 3.5.5. Derivatization with l-FDAA

Fmoc deprotection was achieved by the addition of 20% of piperidine in DMF (twice, 5 and 10 min), a 5 min DMF flow-wash, followed by coupling with l-FDAA reagent, 1% solution in acetone (3.2 eq.), in the presence of DIPEA (105 µL, 0.6 mmol, 6.0 eq.) for 1 h. The resin was washed with acetone (5 × 1 min), dry CH_2_Cl_2_ (5 × 1 min), 1:1 CH_2_Cl_2_/MeOH (5 × 1 min) and MeOH (2 × 1 min), then dried (vacuum desiccator).

#### 3.5.6. Cleavage of l-FDAA Derivatized Dipeptide from Resin

After swelling the 2-CTC resin for 20 min in dry CH_2_Cl_2_ (2 mL) the resin was mixed with 20% hexafluoro-2-propanol (HFIP)/CH_2_Cl_2_ (2 mL × 3 × 20 min) and the combined filtrate evaporated in vacuo to yield analytical samples of l-FDAA-d-*allo*-Ile-l-Ala [HRESI(+)MS *m*/*z* 477.1722 [M + Na]^+^ (calcd for C_18_H_26_N_6_O_8_Na 477.1704) and l-FDAA-d-*allo*-Ile-d-Ala [HRESI(+)MS *m*/*z* 477.1692 [M + Na]^+^ (calcd for C_18_H_26_N_6_O_8_Na 477.1704), both of which were shown to be pure by HPLC-DAD-MS (method as described in general experimental section) (Figures S46 and S47).

#### 3.5.7. Marfey’s Method #6 Optimized for l-FDAA-d-*allo*-Ile-Ala Diastereomers

An aliquot of Marfey’s derivatized analyte (3 μL) (see method #5) was subjected to UPLC-DAD-MS analysis using the same binary solvent system and detection as described above (method #2), but with an isocratic 0.6 mL/min elution at 37% Phase B in A, and with comparison between natural and synthetic Marfey’s derivatized dipeptides (Figure S41).

### 3.6. Synthesis of Talarolide B

#### 3.6.1. Coupling of the First Amino Acid

After swelling the 2-CTC resin for 20 min in dry CH_2_Cl_2_ (2 mL), a solution of Fmoc-Gly-OH (1.2 eq.) and DIPEA (44 µL, 0.25 mmol, 2.5 eq.) in dry CH_2_Cl_2_ (2 mL) was added to the resin and mixed for 2 h. The resin was filtered, then MeOH (200 µL) was added and mixed for 15 min to cap the resin. The resin was washed with dry CH_2_Cl_2_ (5 × 1 min), 1:1 CH_2_Cl_2_/MeOH (5 × 1 min) and MeOH (2 × 1 min).

#### 3.6.2. Elongation of Peptide Sequence

Amino acid activation was achieved by dissolving an Fmoc-amino acid (0.32 mmol, 3.2 eq.) in a 0.4 M HATU/DMF solution (0.75 mL, 0.3 mmol, 3.0 eq.), followed by the addition of DIPEA (105 μL, 0.6 mmol, 6.0 eq.). The coupling cycle consisted of Fmoc deprotection with 20% of piperidine in DMF (twice, 5 and 10 min), and a 5 min DMF flow-wash followed by coupling with preactivated Fmoc-amino acid (3.2 eq.) over 2 × 30 min, or 2 × 3 h for coupling of Fmoc-amino acids to sterically hindered *N*-Me-amino acids. Upon completion of the synthesis the resin was washed with DMF, CH_2_Cl_2_, and MeOH, then dried (vacuum desiccator) as described above in the synthesis of the dipeptides.

#### 3.6.3. Cleavage of Linear Protected Peptide

After swelling the 2-CTC resin for 20 min in dry CH_2_Cl_2_ (2 mL), the resin was mixed with 20% hexafluoro-2-propanol (HFIP)/CH_2_Cl_2_ (2 mL × 3 × 20 min and the combined filtrate concentrated in vacuo to give the protected linear peptide (64 mg). The product was confirmed by HPLC-DAD-MS (method as described in general experimental section): *t*_R_ = 4.4 min, ESI(+)MS *m*/*z* 776 [M + H]^+^, and was used in the next step without further purification.

#### 3.6.4. Cyclization of Linear Protected Peptide

A solution of the linear protected peptide (0.5 mg/mL in DMF (128 mL, 64 mg, 0.082 mmol) was stirred vigorously and treated by dropwise addition over 30 min with a mixture of 0.4 M HATU (618 μL, 0.247 mmol, 3 eq.), hydroxybenzotriazole (HOBT) (34 mg, 0.247 mmol, 3 eq.) and collidine (33 µL, 30 mg, 0.247mmol, 3 eq.) in DMF (2 mL). After 14 h, HPLC-DAD-MS analysis of the mixture showed the cyclization was completed. The DMF was evaporated, and the residue dissolved in MeCN (10 mL), filtered (0.45 µm filter), and purified by preparative HPLC (Agilent Zorbax Rx-C_8_ 7 μm, 21.2 × 250 mm column, with a 20 min gradient elution at 20 mL/min from 90% H_2_O/MeCN to 100% MeCN with an isocratic 0.01% trifluoroacetic acid/MeCN modifier). After lyophilization, the protected cyclic peptide was obtained as an amorphous powder. The product was confirmed by HPLC-DAD-MS (method as described in general experimental section): *t*_R_ = 5.8 min, ESI(+)MS *m*/*z* 758.5 [M + H]^+^

#### 3.6.5. Cyclic Peptide Deprotection

The protected cyclic peptide was mixed with an aqueous solution of 90% formic acid (3 mL) for 40 min, after which it was concentrated under a stream of nitrogen gas and the residue dissolved in MeCN (2 mL) and purified by preparative HPLC (Agilent Zorbax Rx-C_8_ 7 μm, 21.2 × 250 mm column, with a 20 min gradient elution at 20 mL/min from 90% H_2_O/MeCN to 100% MeCN with an isocratic 0.01% trifluoroacetic acid/MeCN modifier) to yield synthetic talarolide B (16 mg, 23% overall yield); NMR (DMSO-*d*_6_), see Figures S50–S52; HRESI(+)MS *m*/*z* 724.4008 [M + Na]^+^ (calcd for C_35_H_55_N_7_O_8_Na 724.4004); identical with natural talarolide B (**2**), including by co-injection HPLC-DAD-MS (Figure S48).

### 3.7. Three-Dimensional Solution Structure Calculations

The distance restraints used in calculating the structure for talarolide A (**1**) in DMSO-*d*_6_ were derived from ROESY spectra (recorded at 298 K) using mixing time (spin-lock) of 300 ms with 41 NOEs (see Supplementary Materials). NOE cross-peak volumes were classified manually as strong (upper distance constraint ≤ 2.7Å), medium (≤3.5Å), weak (≤5.0Å), or very weak (≤6.0Å). Standard pseudoatom distance corrections were applied for non-stereospecifically assigned protons. To address the possibility of conformational averaging, intensities were classified conservatively and only upper distance limits were included in the calculations to allow the largest possible number of conformers to fit the experimental data. Backbone dihedral angle restraints were inferred from ^3^*J*_NHCHα_ coupling constants in 1D spectra, using the Karplus equation [15] with angle ± 30°. There was one *cis*-amides bond between *N*-Me-l-Ala^6^-*N*-Me-l-Tyr^7^ (i.e., strong CHα-CHα (*i*, *i + 1*) NOEs); the rest were in *trans* configuration (ψ-angles were set to *trans* (ψ = 180˚)). Starting structures with randomized ϕ and ψ angles and extended side chains were generated using an ab initio simulated annealing protocol. The calculations were performed using the standard forcefield parameter set (PARALLHDG5.2.PRO) and topology file (TOPALLHDG5.2.PRO) in XPLOR-NIH with in-house modifications to generated N-methylated and *N*-hydroxylated residues. Refinement of structures was achieved using the conjugate gradient Powell algorithm with 4000 cycles of energy minimization and a refined forcefield based on the program CHARMM [12]. Structures were visualized with Pymol and analyzed for distance (>0.2Å) and dihedral angle (>2°) violations using noe.inp files. ^1^H NMR (DMSO-*d*_6_) variable temperatures for **1** was obtained from 298 K to 318 K in five degrees stepwise on a Bruker Avance III 600 MHz NMR spectrometer. The chemical shift differences of amide NH and *N*-OH were plotted against temperature to generate the temperature coefficient (Δδ/T) using Prism version 10.0.2 (Figure 8).

## 4. Conclusions

Application of an integrated program of cultivation (MATRIX) and chemical (HPLC-DAD and GNPS) profiling to the marine-derived fungus *Talaromyces* sp. CMB-TU011 enabled access to talarolide A (**1**), along with three new analogues, talarolides B–D (**2**–**4**). Detailed spectroscopic analysis, supported by chemical degradation and derivatization, and partial and total syntheses, permitted assignment of structures to **1** (revised) and **2**–**4**. The talarolides include rare examples of natural cyclic peptides incorporating a hydroxamate moiety, with a solution structure on **1** revealing H-bonding from the *N*-OH-Gly^1^ across the macrocyclic ring to the d-Ala^5^ carbonyl oxygen, which both defined and stabilized a unique conformation. This contrasts with the deoxy analogue **2** (i.e., Gly^1^) where the NMR data indicate two interconverting conformers. Knowledge of the talarolides draws attention to the possible inclusion of hydroxamate moieties in other cyclic peptides (natural and synthetic), as a means to access new and unusual conformations with potentially new biological properties including improved oral bioavailability [16].

## Figures and Tables

**Table 1:** ^1^H NMR (DMSO-*d*_6_) data for talarolides A–D (**1**–**4**).

	1 *δ*_H_, Mult, (*J* in Hz)	2 *δ*_H_, Mult, (*J* in Hz) ^e^	3 *δ*_H_, Mult, (*J* in Hz)	4 *δ*_H_, Mult, (*J* in Hz)
*N*-OH-Gly^1^/Gly^1^				
2a	4.75, d (17.1)	4.14 ^a^	4.75 ^a^	4.80 d (17.0)
2b	3.76, d (17.1)	3.54, dd (17.3, 2.9)	3.76, d (17.2)	3.71, d (17.0)
*N*-OH	9.31, s		9.41, s	9.13, s
*N*-H		7.53, dd (7.6, 2.9)		
l-Ala^2^				
2	4.49, qd (6.8, 4.1)	4.67, qd (6.7, 5.9)	4.50, qd (6.7, 4.0)	4.49, qd (6.8, 4.1)
3	1.19, d (6.8)	1.20, d (6.7)	1.20, d (6.7)	1.19, d (6.8)
*N*-H	8.65, d (4.1)	8.59, d (5.9)	8.65, d (4.0)	8.62, d (4.1)
*N*-Me-d-Leu^3^				
2	5.05, dd (11.8, 3.9)	5.05, dd (11.8, 3.8)	5.07, dd (11.6, 3.8)	5.06, dd (11.7, 3.8)
3a	1.79, ddd (14.4, 10.3, 3.9)	1.82, ddd (14.4, 10.5, 3.8)	1.79, ddd (14.4, 10.3, 3.8)	1.79, ddd (13.4, 10.6, 3.9)
3b	1.58, ddd (14.4, 11.8, 3.9)	1.61, ddd (14.4, 11.8, 3.7)	1.57, ddd (14.4, 11.6, 3.9)	1.57, ddd (13.4, 11.7, 3.9)
4	1.37, m	1.39, m	1.38, m	1.38 ^a^
5	0.77, d (6.5)	0.79, d (6.5)	0.78, d (6.5)	0.77, d (6.5)
6	0.88, d (6.5)	0.90, d (6.5)	0.89, d (6.5)	0.88, d (6.6)
*N*-Me	3.00, s	3.11, s	3.01, s	2.98, s
d-*allo*-Ile^4^/d-Val^4^				
2	4.72 ^a^	4.60, dd (9.3, 5.2)	4.58, dd (9.5, 4.9)	4.69, dd (9.5, 3.9)
3	1.95, m	1.95, m	2.17, m	1.91, m
4a	1.42, m	1.56, m	0.92, d (6.8)	1.39 ^a^
4b	1.07, m	1.09 ^b^		1.05, m
5	0.94, dd (7.3, 7.3)	0.95, dd (7.3, 7.3)	0.86, d (6.8)	0.92, dd (7.3, 7.3)
6	0.81, d (6.9)	0.92, d (6.9)		0.80, d (6.9)
*N*-H	7.24, d (9.6)	6.96, d (9.3)	7.23, d (9.5)	7.14, d (9.6)
d-Ala^5^				
2	4.34, qd (7.1, 5.4)	4.42 ^c^	4.33, qd (7.1, 5.0)	3.90, m
3	1.12, d (7.1)	1.10 ^b^, d (7.1)	1.12, d (7.1)	1.13, d (7.1)
*N*-H	8.87, d (5.4)	8.63, d (5.4)	8.87, d (5.0)	8.58, d (5.2)
*N*-Me-l-Ala/l-Ala^6^				
2	4.71 ^a^	4.43 ^c^	4.75 ^a^	4.37, m
3	0.49, d (6.5)	0.58, d (6.5)	0.52, d (6.5)	0.63, d (6.4)
*N*-Me	2.70, s	2.70, s	2.71, s	
*N*-H				8.27, d (9.5)
*N*-Me-l-Tyr^7^				
2	4.80, dd (10.5, 4.9)	4.15 ^a^	4.80, dd (9.8, 5.3)	5.01, dd (8.6, 6.6)
3a	2.84, dd (14.3, 10.5)	2.93, dd (14.1, 11.6)	2.82, dd (14.2, 9.8)	2.81, dd (14.3, 6.6)
3b	2.60, dd (14.3, 4.9)	2.55 ^d^	2.62, dd (14.2, 5.3)	2.77, dd (14.3, 8.6)
5/9	6.93, d (8.4)	6.90, d (8.4)	6.94, d (8.4)	6.99, d (8.4)
6/8	6.64, d (8.4)	6.63, d (8.4)	6.63, d (8.4)	6.63, d (8.4)
7-OH	9.20, s	9.25, s	9.18, s	9.15, s
*N*-Me	2.66, s	2.86, s	2.67, s	2.64, s

^a–c^ resonances with the same superscript within a column are overlapping, ^d^ signal is obscured by DMSO, detected by HSQC. ^e^ occurs as an equilibrating mixture of major and minor conformers, with the major conformer tabulated.

**Table 2:** ^13^C NMR (DMSO-*d*_6_) data for talarolides A–D (**1**–**4**).

	1 *δ*_C_	2 *δ*_C_	3 *δ*_C_	4 *δ*_C_
*N*-OH-Gly^1^/Gly^1^				
1	167.3	169.3	167.3	167.5
2	50.2	41.3	50.1	49.9
l-Ala^2^				
1	174.1	174.4	174.1	174.0
2	45.2	44.6	45.2	45.2
3	15.7	15.8	15.7	15.7
*N*-Me-d-Leu^3^				
1	169.5	169.2 ^a^	169.5	169.5
2	54.5	54.6 ^b^	54.4	54.4
3	36.0	36.1	35.9	36.0
4	24.4	24.4	24.4	24.4
5	21.0	20.9	20.9	20.9
6	23.3	23.3	23.3	23.3
*N*-Me	31.0	30.8	31.0	30.9
d-*allo*-Ile^4^/d-Val^4^				
1	172.0	171.2	171.6	171.8
2	53.7	54.6 ^b^	55.3	53.8
3	38.5	38.5	31.9	38.8
4	26.2	25.7	19.4	26.4
5	12.0	12.0	17.0	11.9
6	13.7	14.2 ^c^		13.7
d-Ala^5^				
1	171.1	171.6	171.0	170.8
2	45.8	45.7	45.8	48.6
3	14.9	14.6	14.9	16.7
*N*-Me-l-Ala/l-Ala^6^				
1	169.8	169.8	169.8	171.4
2	46.7	49.3	46.7	42.7
3	15.1	14.2 ^c^	15.1	18.5
*N*-Me	28.6 ^a^	29.1	28.6 ^a^	
*N*-Me-l-Tyr^7^				
1	168.2	169.2 ^a^	168.2	168.3
2	56.6	59.8	56.6	57.1
3	34.1	34.8	34.3	34.5
4	126.6	126.5	126.7	127.2
5/9	130.8	130.3	130.7	130.6
6/8	114.8	114.9	114.8	114.9
7	155.9	156.0	155.9	155.8
*N*-Me	28.6 ^a^	29.5	28.6 ^a^	28.6

^a–c^ resonances with the same superscript within a column are interchangeable.

## Data Availability

Raw NMR data have been deposited in the Natural Product Magnetic Resonance Database Project (NP-MRD).

## Supplementary Materials

The following supporting information can be downloaded at: https://www.mdpi.com/article/10.3390/md21090487/s1. MATRIX study of CMB TU011; NMR spectroscopic data (tabulated data and spectra), Marfey’s analysis, and MS/MS spectra of **1**–**4**; NMR spectra comparison of natural and synthetic talarolide B (**2**); 3D calculations of talarolide A (**1**).

## References

[1] Raju R., Khalil Z.G., Piggott A.M., Blumenthal A., Gardiner D.L., Skinner-Adams T.S., Capon R.J. (2014). Mollemycin A: An Antimalarial and Antibacterial Glyco-Hexadepsipeptide-Polyketide from an Australian Marine-Derived *Streptomyces* sp. (CMB-M0244). Org. Lett..

[2] Khalil Z.G., Salim A.A., Lacey E., Blumenthal A., Capon R.J. (2014). Wollamides: Antimycobacterial Cyclic Hexapeptides from an Australian Soil *Streptomyces*. Org. Lett..

[3] Jiao W.-H., Khalil Z., Dewapriya P., Salim A.A., Lin H.-W., Capon R.J. (2018). Trichodermides A-E: New Peptaibols Isolated from the Australian Termite Nest-Derived Fungus *Trichoderma virens* CMB-TN16. J. Nat. Prod..

[4] Quezada M., Shang Z., Kalansuriya P., Salim A.A., Lacey E., Capon R.J. (2017). Waspergillamide A, a Nitro Depsi-Tetrapeptide Diketopiperazine from an Australian Mud Dauber Wasp-Associated *Aspergillus* sp. (CMB-W031). J. Nat. Prod..

[5] Elbanna A.H., Khalil Z.G., Bernhardt P.V., Capon R.J. (2019). Scopularides Revisited: Molecular Networking Guided Exploration of Lipodepsipeptides in Australian Marine Fish Gastrointestinal Tract-Derived Fungi. Mar. Drugs.

[6] Dewapriya P., Khalil Z.G., Prasad P., Salim A.A., Cruz-Morales P., Marcellin E., Capon R.J. (2018). Talaropeptides A-D: Structure and Biosynthesis of Extensively N-Methylated Linear Peptides from an Australian Marine Tunicate-Derived *Talaromyces* sp. Front. Chem..

[7] Dewapriya P., Prasad P., Damodar R., Salim A.A., Capon R.J. (2017). Talarolide A, a Cyclic Heptapeptide Hydroxamate from an Australian Marine Tunicate-Associated Fungus, *Talaromyces* sp. (CMB-TU011). Org. Lett..

[8] Zhang S., De Leon Rodriguez L.M., Huang R., Leung I.K.H., Harris P.W.R., Brimble M.A. (2018). Total Synthesis of the Proposed Structure of Talarolide, A. Org. Biomol. Chem..

[9] Salim A.A., Khalil Z.G., Elbanna A.H., Wu T., Capon R.J. (2021). Methods in Microbial Biodiscovery. Mar. Drugs.

[10] Aron A.T., Gentry E.C., McPhail K.L., Nothias L.-F., Nothias-Esposito M., Bouslimani A., Petras D., Gauglitz J.M., Sikora N., Vargas F. (2020). Reproducible Molecular Networking of Untargeted Mass Spectrometry Data Using GNPS. Nat. Protoc..

[11] Vijayasarathy S., Prasad P., Fremlin L.J., Ratnayake R., Salim A.A., Khalil Z., Capon R.J. (2016). C_3_ and 2D C_3_ Marfey’s Methods for Amino Acid Analysis in Natural Products. J. Nat. Prod..

[12] Brooks B.R., Bruccoleri R.E., Olafson B.D., States D.J., Swaminathan S., Karplus M. (1983). Charmm: A Program for Macromolecular Energy, Minimization, and Dynamics Calculations. J. Comput. Chem..

[13] Brunger A.T. (1992). X-Plor Manual Version 3.1.

[14] Wishart D.S., Sykes B.D., Richards F.M. (1992). The Chemical Shift Index: A Fast and Simple Method for the Assignment of Protein Secondary Structure through NMR Spectroscopy. Biochemistry.

[15] Pardi A., Billeter M., Wüthrich K. (1984). Calibration of the Angular Dependence of the Amide Proton-Cα Proton Coupling Constants, 3_JHNα_, in a Globular Protein: Use of 3_JHNα_ for Identification of Helical Secondary Structure. J. Mol. Biol..

[16] Nielsen D.S., Hoang H.N., Lohman R.-J., Hill T.A., Lucke A.J., Craik D.J., Edmonds D.J., Griffith D.A., Rotter C.J., Ruggeri R.B. (2014). Improving on Nature: Making a Cyclic Heptapeptide Orally Bioavailable. Angew. Chem..

