# Supplementary material for: Talarolides Revisited: Cyclic Heptapeptides from an Australian Marine Tunicate-Associated Fungus, Talaromyces sp. CMB-TU011
Source: Mar Drugs. 2023 Sep 11;21(9):487. doi: 10.3390/md21090487 (PMC10533135; doi:10.3390/md21090487)
Supplement: Supplementary file 1 [file marinedrugs-21-00487-s001.zip › marinedrugs-2579786-supplementary.pdf]

Supplementary Materials

Talarolides Revisited, Cyclic Heptapeptides from an Australian Marine Tunicate-Associated Fungus, *Talaromyces* sp. CMB-TU011

Angela A. Salim[1,#], Waleed M. Hussein[1,#], Pradeep Dewapriya[1,2,#], Huy N. Hoang[1], Yahao Zhou[1], Kaumadi Samarasekera[1,3], Zeinab G. Khalil[1], David P. Fairlie[1] and and Robert J. Capon[1,*]

[1] Institute for Molecular Bioscience; The University of Queensland, St Lucia, QLD 4072, Australia;
[2] Current address: Queensland Alliance for Environmental Health Science, The University of Queensland, Wooloongabba, QLD 4102.
[3] Current address: Department of Botany, Faculty of Science, University of Peradeniya, Peradeniya 204000, Sri Lanka.

# These authors contributed equally
* Correspondence: r.capon@uq.edu.au (R.J.C.); Tel.: +61-7-3346-2979

Table of Contents

1. MATRIX study of CMB-TU011 metabolites 3
2. Spectroscopic data for talarolides A-D 5
   2.1. Talarolide A (**1**) 5
   2.2. Talarolide B (**2**) 10
   2.3. Talarolide C (**3**) 16
   2.4. Talarolide D (**4**) 22
3. Marfey's analysis of talarolides A-D 28
4. MSMS analysis of talarolides 36
5. Synthetic dipeptides and synthetic talarolide B 40
6. ROE and distance restraints for talarolide A in DMSO-d6 46

1. MATRIX study of CMB-TU011 metabolites

Table S1: Media composition for the MATRIX

**Media**	**Composition** (Per Liter)
M1 agar	Peptone (2.0 g), Yeast extract (4.0 g), Starch (10.0 g), Agar (18.0 g). pH 7.0
M1 salt agar	Peptone (2.0 g), Yeast extract (4.0 g), Starch (10.0 g), ocean sea salt (33 g), Agar (18.0 g). pH 7.0
ISP4 agar	Soluble starch (10.0 g), $CaCO_3$ (2.0 g), $(NH_4)_2SO_4$ (2.0 g), K_2HPO_4 (1.0 g), $MgSO_4.7H_2O$ (1.0 g), NaCl (1.0 g), $FeSO_4.7H_2O$ (1 mg), $MnCl_2.7H_2O$ (1.0 mg), $ZnSO_4.7H_2O$ (1.0 mg), Agar (20.0 g). pH 7.2
Tryptic soy agar	Pancreatic digest of casein (15.0 g), Papaic digest of soybean (5.0 g), NaCl (5.0 g), Agar (15.0 g). pH 7.3
Potato dextrose agar	Potato extract (4.0 g), Dextrose (20.0 g), Agar (15.0 g). pH 5.6
Oatmeal agar	Oatmeal (60 g), Agar (12.4 g)
M2 agar	Manitol (40.0 g), Maltose (40.0 g), Yeast extract (10.0 g), K_2HPO_4 (2.0 g), $MgSO_4.7H_2O$ (0.5 g), FeSO4.7H2O (0.01 g). pH 7.0
PYG agar	Peptone (1.25 g), Yeast Extract (1.25 g) Dextrose (3.0 g) Agar (20.0 g). pH 7.0
Basal agar	K_2HPO_4 (1.5 g), $MgSO_4.7H_2O$ (0.025 g), $CaCl_2$ (0.025 g), $FeSO_4.7H_2O$ (0.015 g), and $ZnSO_4.7H_2O$ (0.005). pH 7.8
Czapeck agar	K_2HPO_4 (1.0 g), $NaNO_3$ (0.3 g), KCl (0.005 g), $MgSO_4$.7H2O (0.005 g), $FeSO_4$ (0.0001 g), Sucrose (30 g). pH 7.0
YES agar	Sucrose (150 g), Yeat extract (20 g), $MgSO_4.7H_2O$ (0.5 g), $ZnSO_4.7H_2O$ (0.01 g), $CuSO_4.5H_2O$ (0.005 g). pH 7.0
ISP2 agar	Yeast extract (4.0 g), Malt extract (10.0 g), Glucose (4.0 g), Agar (20.0 g). pH 7.2

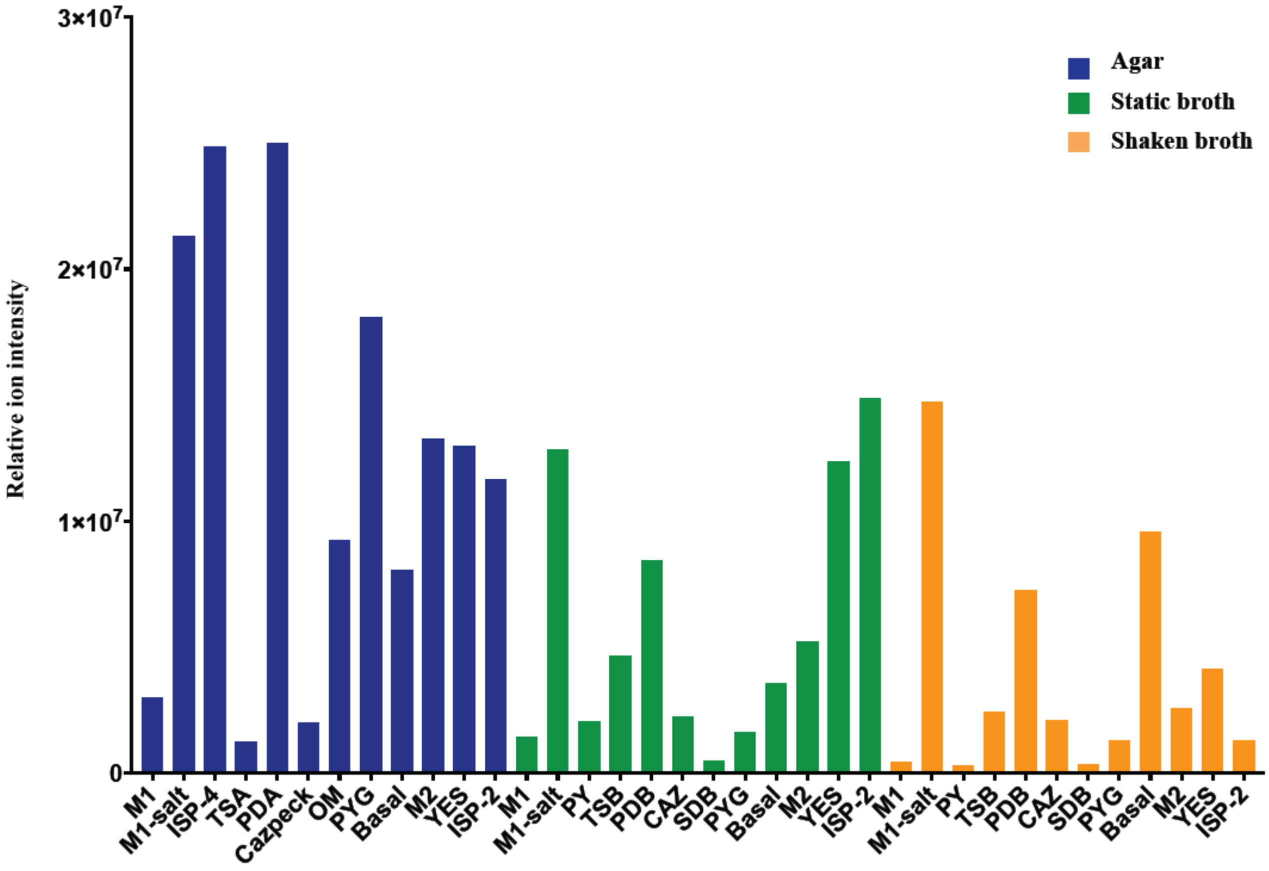

Figure S1. Talarolide A (**1**) production under different culture conditions. The expression level of talarolide A production was calculated based on the area under the peak for SIE at m/z 718 $(M+H)^+$ in HPLC-DAD-ESI(+)MS analysis.

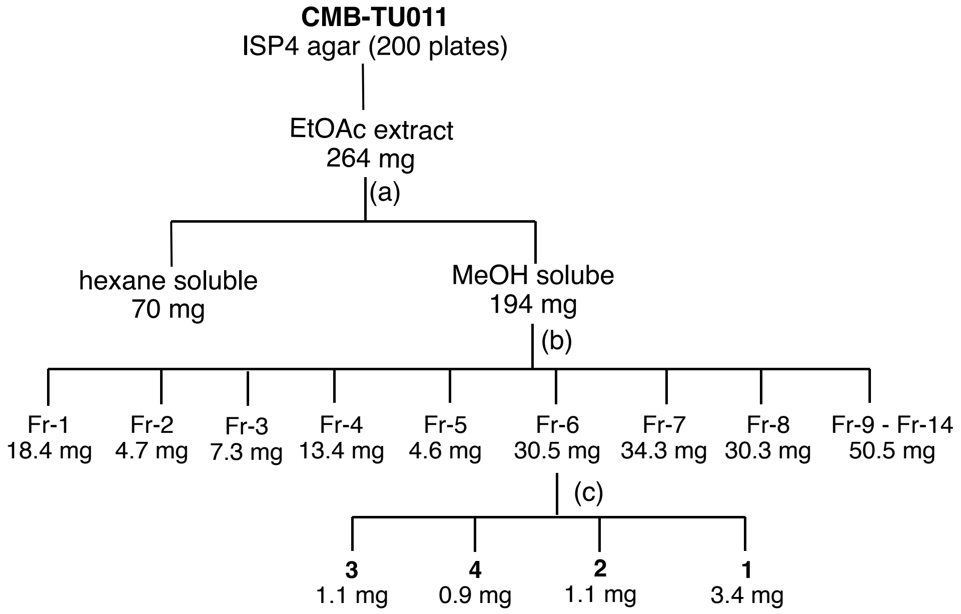

(a) Partition [*n*-hexane / aqueous MeOH]
(b) Sephadex LH-20, MeOH
(c) Semiprep HPLC, Zorbax eclipse C_8, 32% MeCN/H_2O isocratic (0.01% TFA), 3 mL/min

Figure S2. Isolation scheme for CMB-TU011

2. Spectroscopic data for talarolides A-D

2.1. Talarolide A (**1**)

Table S2: NMR (DMSO-d_6) data for talarolide A (**1**)

		δ_H, mult, (*J* in Hz)	COSY	HMBC	ROESY
N-OH-Gly[1]					
1	167.3	-	-	-	-
2	50.2	a 4.75, d (*17.1*)<br>b 3.76, d (*17.1*)	2b<br>2a	1<br>1	*N*-H(L-Ala)<br>*N*-OH, *N*-H(L-Ala)
N-OH		9.31, s	-	-	2b, 2(D-Ala), 2(*N*-Me-L-Tyr), *N*-H(D-*allo*-Ile), 6(D-*allo*-Ile), *N*-Me(*N*-Me-L-Ala)
L-Ala[2]					
1	174.1	-	-	-	-
2	45.2	4.49, qd (*6.8*, *4.1*)	3, *N*-H	3	3, *N*-H, *N*-Me(*N*-Me-D-Leu)
3	15.7	1.19, d (*6.8*)	2	1, 2	2, *N*-H
N-H		8.65, d (*4.1*)	2	2, 3, 1(*N*-OH-Gly)	2, 3, 2a(*N*-OH-Gly), 2b(*N*-OH-Gly)
N-Me-D-Leu[3]					
1	169.5	-	-	-	-
2	54.5	5.05, dd (*11.8*, *3.9*)	3a, 3b	1, 3, *N*-Me, 1(L-Ala)	3a, 3b, 4, 5, *N*-Me, *N*-H(D-*allo*-Ile)
3	36.0	a 1.79, ddd (*14.4*, *10.3*, *3.9*)<br>b 1.58, ddd (*14.4*, *11.8*, *3.9*)	2, 3b, 4<br>2, 3a, 4	2, 4, 5, 6<br>2, 5	2, 5, 6<br>4, 6, *N*-Me
4	24.4	1.37, m	3a, 3b, 5, 6	5, 6	2, 3b, 5, 6, *N*-Me
5	21.0	0.77, d (*6.5*)	4	3, 4, 6	2, 3a, 4
6	23.3	0.88, d (*6.5*)	4	3, 4, 5	3a, 3b, 4
N-Me	31.0	3.00, s	-	2, 1(L-Ala)	2, 3b, 4, 2(L-Ala), *N*-H(D-*allo*-Ile), 6(D-*allo*-Ile)
D-*allo*-Ile[4]					
1	172.0	-	-	-	-
2	53.7	4.72[b]	3, *N*-H	1, 3, 6, 1(*N*-Me-D-Leu)	3, 4a, 4b, 5, 6, *N*-H, *N*-H(D-Ala)
3	38.5	1.95, m	2, 4a, 4b, 6	4, 5, 6	2, 4a, 5, *N*-H, *N*-H(D-Ala)
4	26.2	a 1.42, m<br>b 1.07, m	3, 4b, 5<br>3, 4b, 5	2, 3, 5, 6<br>2, 3, 5, 6	2, 3, 5, 6<br>2, 3, 5, 6
5	12.0	0.94, dd (*7.3*, *7.3*)	4a, 4b	3, 4	2, 3, 4a, 4b, 5/9(*N*-Me-L-Tyr)
6	13.7	0.81, d (*6.9*)	3	2, 3, 4	2, 4a, 4b, *N*-H, *N*-Me(*N*-Me-D-Leu), 2(*N*-Me-L-Tyr), 5/9(*N*-Me-L-Tyr), *N*-OH(*N*-OH-Gly)
N-H		7.24, d (*9.6*)	2	2, 1(*N*-Me-D-Leu)	2, 3, 6, 2(*N*-Me-D-Leu), *N*-Me(*N*-Me-D-Leu), *N*-OH(*N*-OH-Gly)
D-Ala[5]					
1	171.1	-	-	-	-
2	45.8	4.34, qd (*7.1*, *5.4*)	3, *N*-H	1, 3	3, *N*-H, *N*-OH(*N*-OH-Gly), *N*-Me(*N*-Me-L-Ala)
3	14.9	1.12, d (*7.1*)	2	1, 2	2, *N*-H, *N*-Me(*N*-Me-L-Ala)
N-H		8.87, d (*5.4*)	2	2, 3, 1(D-*allo*-Ile)	2, 3, 2(D-*allo*-Ile), 3(D-*allo*-Ile)
N-Me-L-Ala[6]					
1	169.8	-	-	-	-
2	46.7	4.71[b]	3	1, 3, *N*-Me, 1(D-Ala)	3, 5/9(*N*-Me-L-Tyr), 6/8(*N*-Me-L-Tyr)
3	15.1	0.49, d *(6.5)*	2	1, 2	2, *N*-Me, 5/9(*N*-Me-L-Tyr), 6/8(*N*-Me-L-Tyr)
N-Me	28.6[a]	2.70, s	-	2, 1(D-Ala)	3, 2(D-Ala), 3(D-Ala), *N*-OH(*N*-OH-Gly),
N-Me-L-Tyr[7]					
1	168.2	-	-	-	-
2	56.6	4.80, dd (*10.5*, *4.9*)	3a, 3b	1, 3, *N*-Me, 1(*N*-Me-L-Ala)	3b, 5/9, *N*-OH(*N*-OH-Gly), 6(D-*allo*-Ile)
3	34.1	a 2.84, dd (*14.3*, *10.5*)<br>b 2.60, dd (*14.3*, *4.9*)	2, 3b<br>2, 3a	2, 4, 5/9<br>2, 4, 5/9	5/9<br>2, 5/9
4	126.6	-	-	-	-
5/9	130.8	6.93, d (*8.4*)	6/8	3, 7, 9/5	2, 3a, 3b, 6/8, *N*-Me, 2(*N*-Me-L-Ala), 3(*N*-Me-L-Ala), 5(D-*allo*-Ile), 6(D-*allo*-Ile)
6/8	114.8	6.64, d (*8.4*)	5/9	4, 7, 8/6	5/9, 7-OH, 2(*N*-Me-L-Ala), 3(*N*-Me-L-Ala)
7	155.9	-	-	-	-
7-OH		9.20, s	-	6/8, 7	6/8
N-Me	28.6[a]	2.66, s	-	2, 1(*N*-Me-L-Ala)	5/9

[a-b]signals within the same superscripts are overlapping

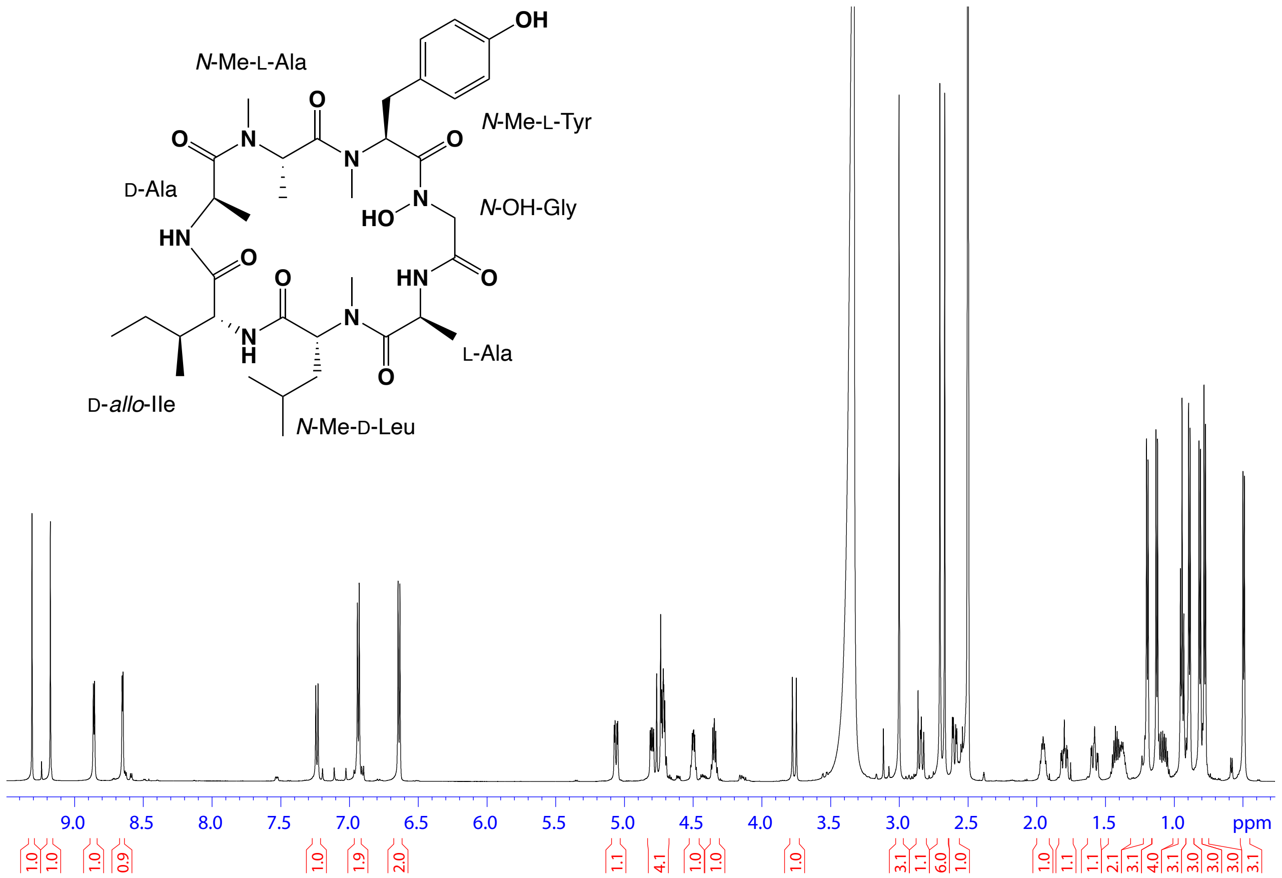

Figure S3. ^{1}H NMR (600 MHz, DMSO-d_6) spectrum of talarolide A (**1**)

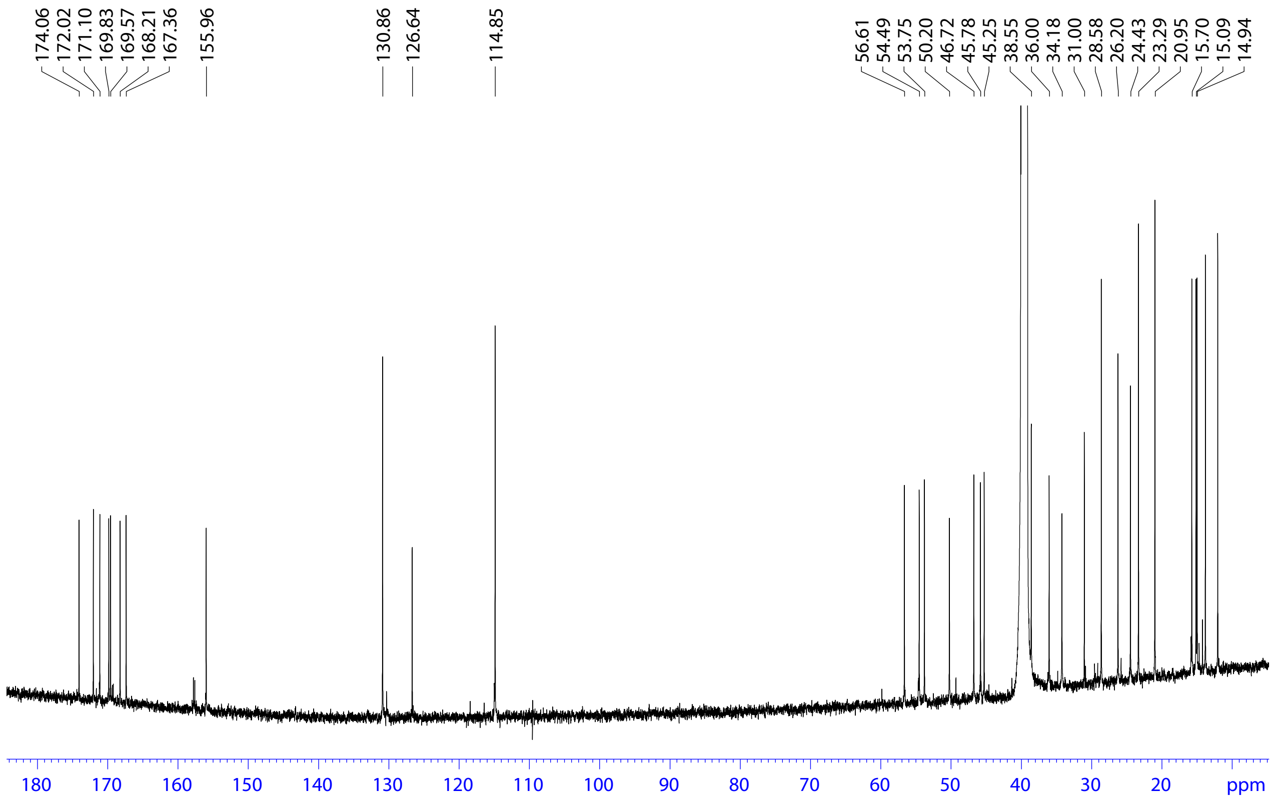

Figure S4. ^{13}C NMR (150 MHz, DMSO-d_6) spectrum of talarolide A (**1**)

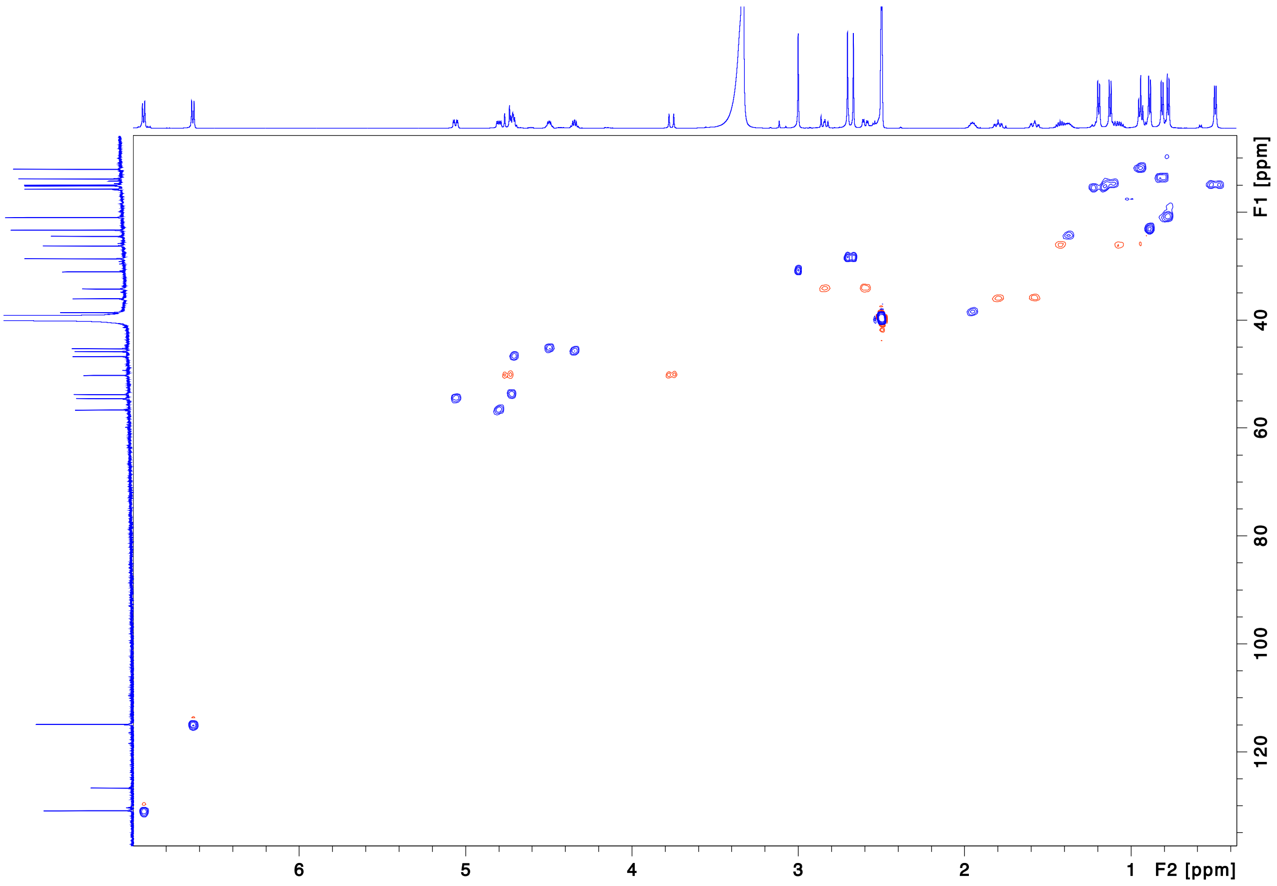

Figure S5. HSQC (600 MHz, DMSO-d_6) spectrum of talarolide A (**1**)

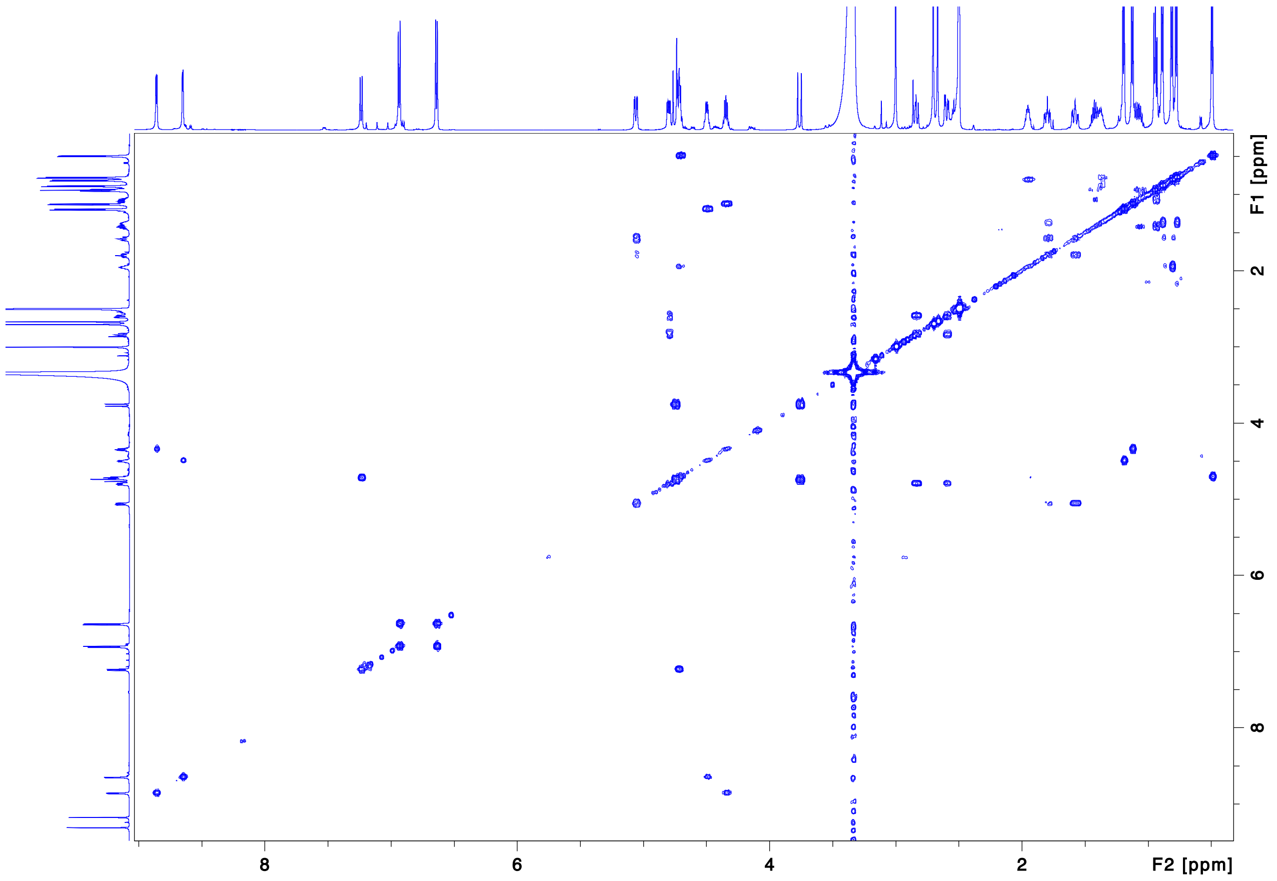

Figure S6. COSY (600 MHz, DMSO-d_6) spectrum of talarolide A (**1**)

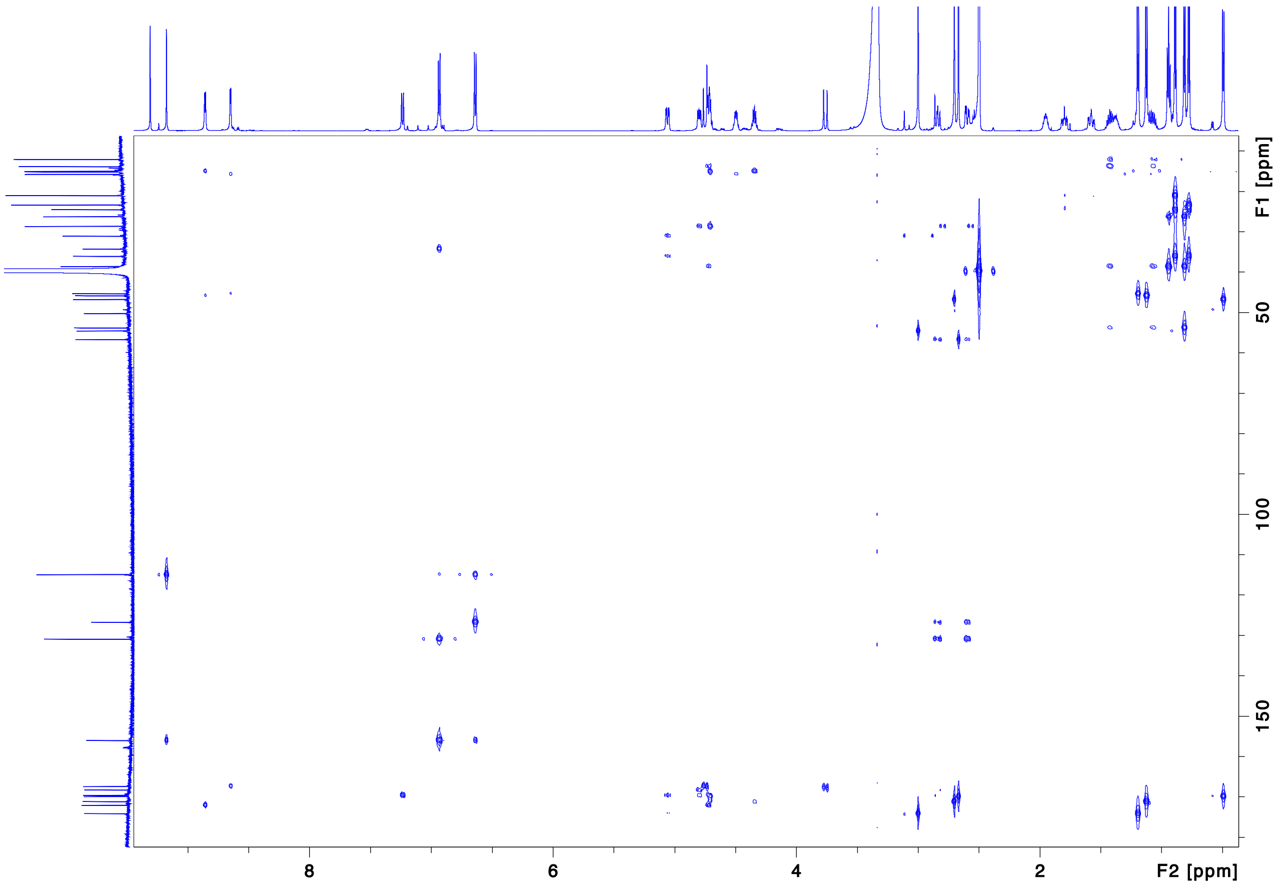

Figure S7. HMBC (600 MHz, DMSO-d_6) spectrum of talarolide A (**1**)

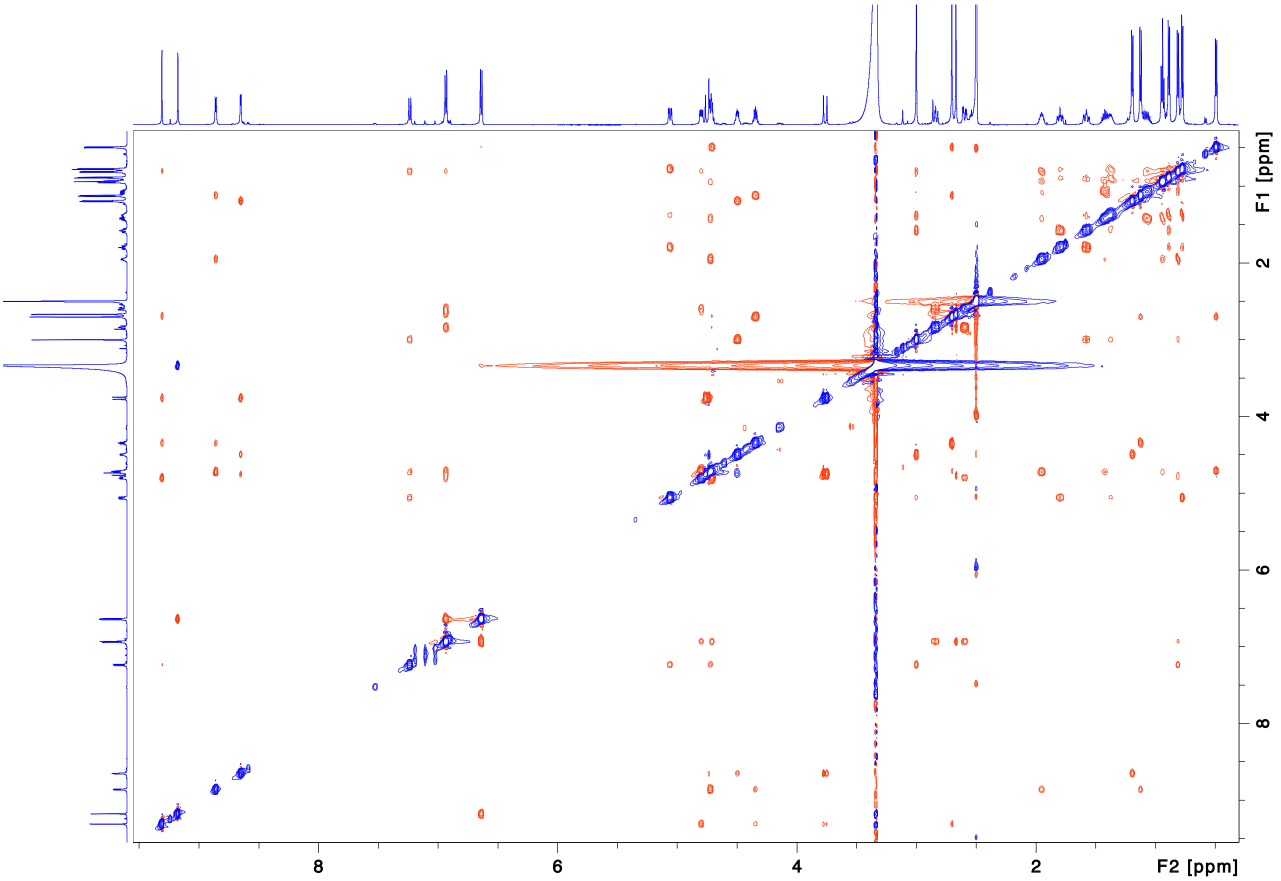

Figure S8. ROESY (600 MHz, DMSO-d_6) spectrum of talarolide A (**1**)

Mass Spectrum Molecular Formula Report

Analysis Info

Analysis Name	D:\Data\a.salim\Talarolide A.d	Acquisition Date	10/21/2022 12:45:34 PM
Method	tune-med_AP.m	Operator	a.salim
Sample Name		Instrument / Ser#	micrOTOF 213750.00232
Comment			

Acquisition Parameter

Source Type	ESI	Ion Polarity	Positive	Set Nebulizer	0.8 Bar
Focus	Not active			Set Dry Heater	180 °C
Scan Begin	100 m/z	Set Capillary	4500 V	Set Dry Gas	5.0 l/min
Scan End	1000 m/z	Set End Plate Offset	-500 V	Set Divert Valve	Source

Generate Molecular Formula Parameter

Formula, min.		
Formula, max.		
Measured m/z	Tolerance	Charge
Check Valence	Minimum	Maximum
Nirogen Rule	Electron Configuration	
Filter H/C Ratio	Minimum	Maximum
Estimate Carbon		

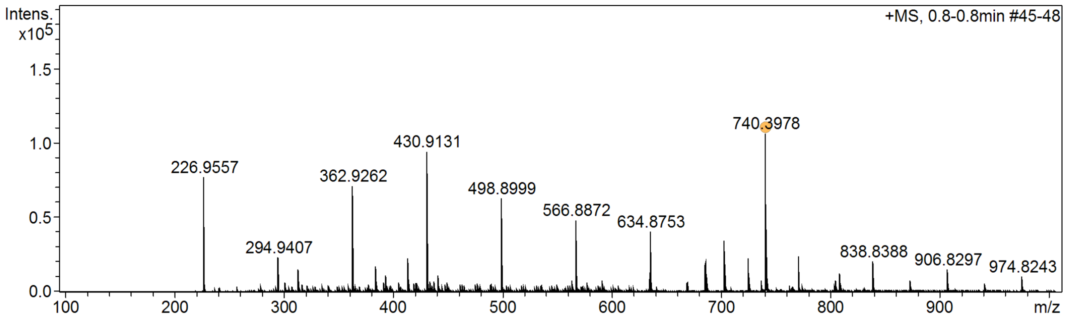

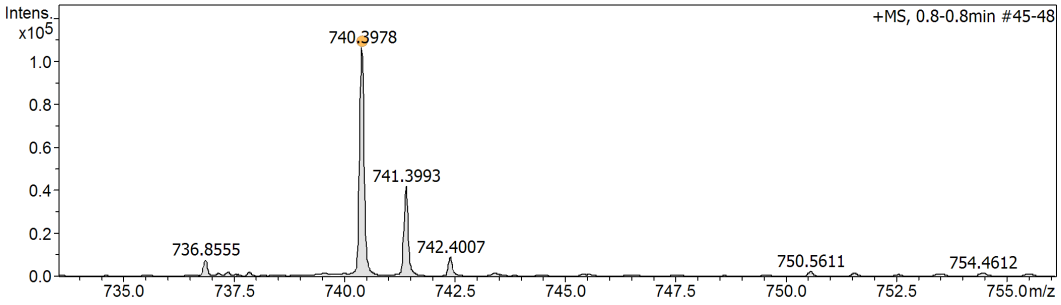

Meas. m/z	#	Ion Formula	m/z	err [ppm]	mSigma	# Sigma	Score	rdb	e¯ Conf	N-Rule
740.3978	1	C35H55N7NaO9	740.3953	3.3	13.1	2	100.00	11.5	even	ok

Figure S9. HRMS measurement for talarolide A (**1**)

2.2. Talarolide B (**2**)

Table S3. NMR (DMSO-d_6) data for talarolide B (**2**)

		δ_H, mult, (*J* in Hz)	COSY	HMBC	ROESY
Gly[1]					
1	169.3	-	-	-	-
2	41.3	a 4.14[d]	2b, *N*-H	1	*N*-H, *N*-H(L-Ala)
		b 3.54, dd (*17.3*, *2.9*)	2a, *N*-H	1	*N*-H, *N*-H(L-Ala)
N-H		7.53, dd (*7.6*, *2.9*)	2a, 2b	1(*N*-Me-L-Tyr)	2a, 2b, 2(*N*-Me-L-Tyr)
L-Ala[2]					
1	174.4	-	-	-	-
2	44.6	4.67, qd (*6.7, 5.9*)	3, *N*-H	1, 3	3, *N*-H, *N*-Me(*N*-Me-D-Leu)
3	15.8	1.20, d (*6.7*)	2	1, 2	2, *N*-H
N-H		8.59, d (*5.9*)	2	2, 3, 1(Gly)	2, 3, 2a(Gly), 2b(Gly)
N-Me-D-Leu[3]					
1	169.2[a]	-	-	-	-
2	54.6[b]	5.05, dd (*11.8*, *3.8*)	3a, 3b	1, 3, *N*-Me	3a, 3b, 4, 5, *N*-Me, *N*-H(D-*allo*-Ile)
3	36.1	a 1.82, ddd (*14.4*, *10.5*, *3.8*)	2, 3b, 4	4	2
		b 1.61, ddd (*14.4, 11.8, 3.7*)	2, 3a, 4	-	2, *N*-Me
4	24.4	1.39, m	3a, 3b, 5, 6	-	2
5	20.9	0.79, d (*6.5*)	4	3, 4, 6	2
6	23.3	0.90, d (*6.5*)	4	3, 4, 5	-
N-Me	30.8	3.11, s	-	2, 1(L-Ala)	2, 3b, 2(L-Ala), *N*-H(D-*allo*-Ile)
D-*allo*-Ile[4]					
1	171.2	-	-	-	-
2	54.6[b]	4.60, dd (*9.3*, *5.2*)	3, *N*-H	1, 3, 6	3, 4a, 5, 6, *N*-H, *N*-H(D-Ala)
3	38.5	1.95, m	2, 4b, 6	4, 5, 6	2, 6, *N*-H(D-Ala)
4	25.7	a 1.56, m	4b, 5	2, 3, 5, 6	2
		b 1.09[e]	3, 4a, 5	2, 3, 5, 6	-
5	12.0	0.95, dd (*7.3, 7.3*)	4a, 4b	3, 4	2
6	14.2[c]	0.92, d (*6.9*)	3	2, 3, 4	2, 3, *N*-H
N-H		6.96, d (*9.3*)	2	1(*N*-Me-D-Leu)	2, 6, 2(*N*-Me-D-Leu), *N*-Me(*N*-Me-D-Leu)
D-Ala[5]					
1	171.6	-	-	-	-
2	45.7	4.42[f]	3, *N*-H	1, 3	3, *N*-H
3	14.6	1.10[e], d (*7.1*)	2	2, 1	2, *N*-H
N-H		8.63, d (*5.4*)	2	2, 3, 1(D-*allo*-Ile)	2, 3, 2(D-*allo*-Ile), 3(D-*allo*-Ile)
N-Me-L-Ala[6]					
1	169.8	-	-	-	-
2	49.3	4.43[f]	3	1, 3, *N*-Me	3, *N*-Me, 2(*N*-Me-L-Tyr), 5/9(*N*-Me-L-Tyr)
3	14.2[c]	0.58, d *(6.5)*	2	1, 2	2
N-Me	29.1	2.70, s	-	2, 1(D-Ala)	2
N-Me-L-Tyr[7]					
1	169.2[a]	-	-	-	-
2	59.8	4.15[d]	3a, 3b	1, *N*-Me	3b, 5/9, *N*-H(Gly), 2(*N*-Me-L-Ala)
3	34.8	a 2.93, dd (*14.1*, *11.6*)	2, 3b	2, 4, 5/9	5/9
		b 2.55[g]	2, 3a	5/9	2, 5/9
4	126.5	-	-	-	-
5/9	130.3	6.90, d (*8.4*)	6/8	3, 7, 9/5	2, 3a, 3b, *N*-Me, 2(*N*-Me-L-Ala)
6/8	114.9	6.63, d (*8.4*)	5/9	4, 7, 8/6	7-OH
7	156.0	-	-	-	-
7-OH		9.25, s	-	6/8, 7	6/8
N-Me	29.5	2.86, s	-	2, 1(*N*-Me-L-Ala)	5/9

[a-f] signals within the same superscripts are overlapping; [g] signal under DMSO peak

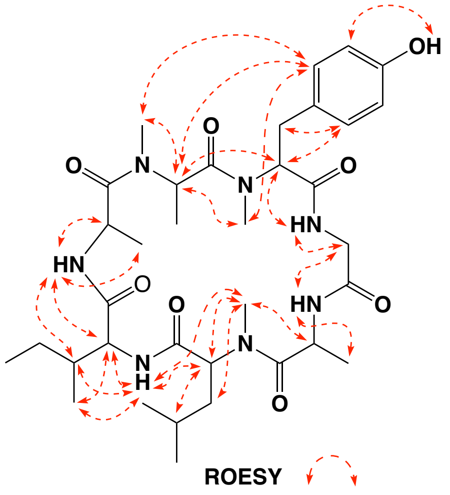

Figure S10. ROESY (DMSO-d_6) correlations for talarolide B (**2**)

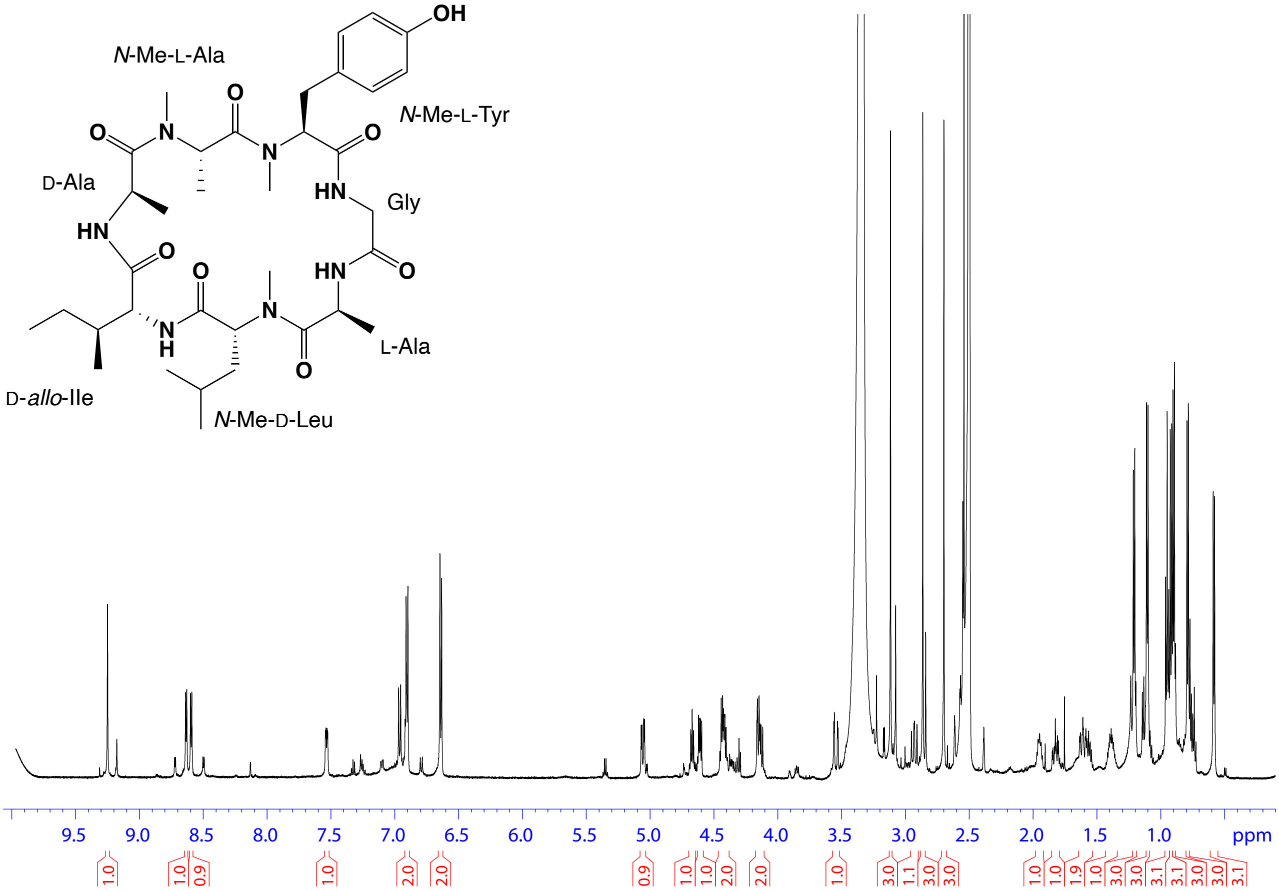

Figure S11. ^{1}H NMR (600 MHz, DMSO-d_6) spectrum of talarolide B (**2**)

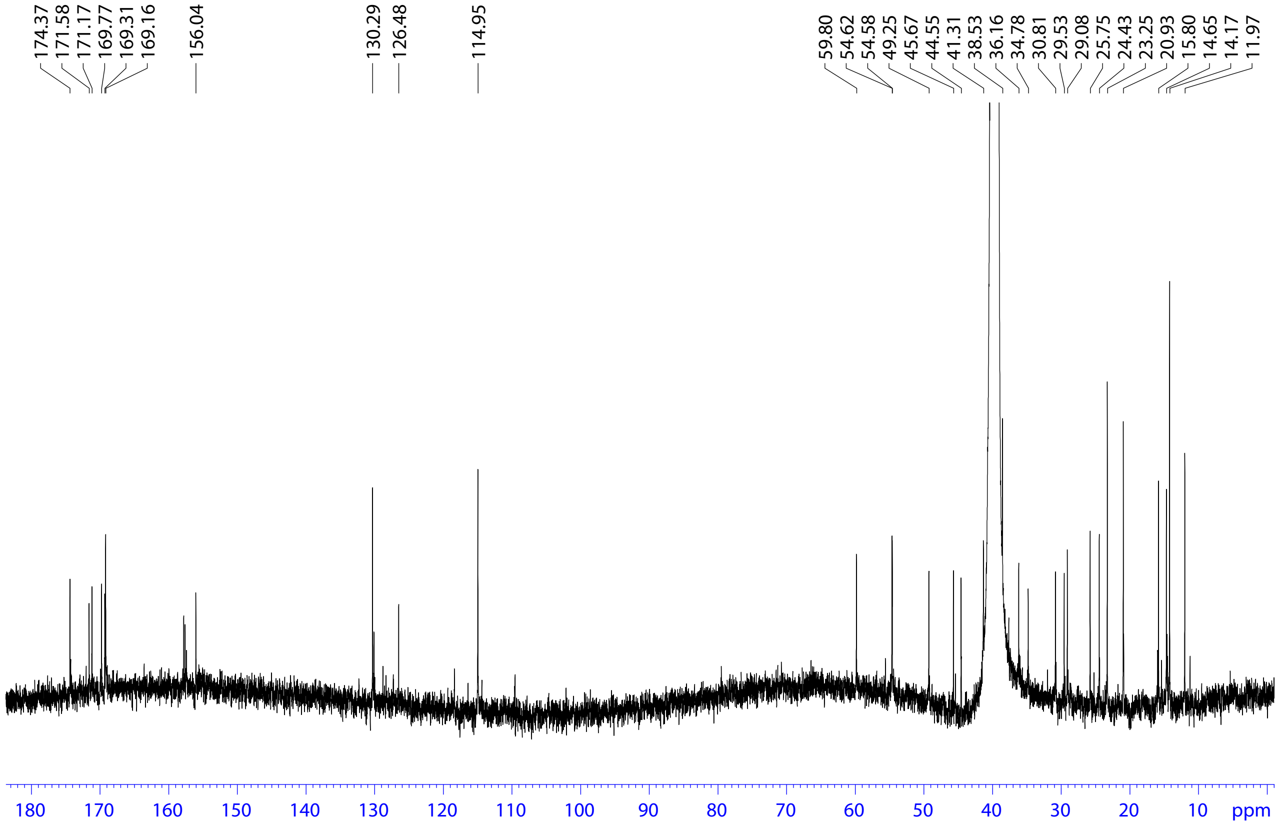

Figure S12. ^{13}C NMR (150 MHz, DMSO-d_6) spectrum of talarolide B (**2**)

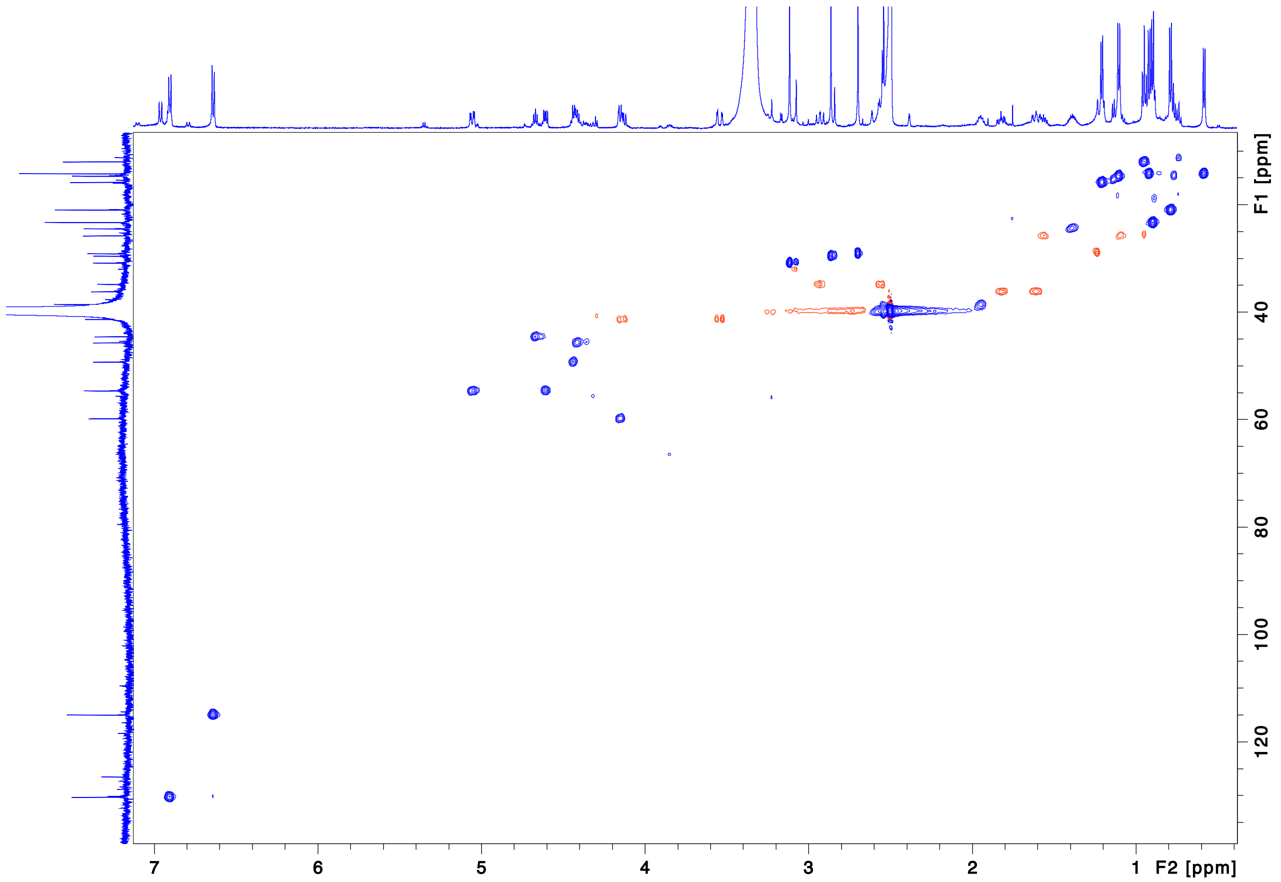

Figure S13. HSQC (600 MHz, DMSO-d_6) spectrum of talarolide B (**2**)

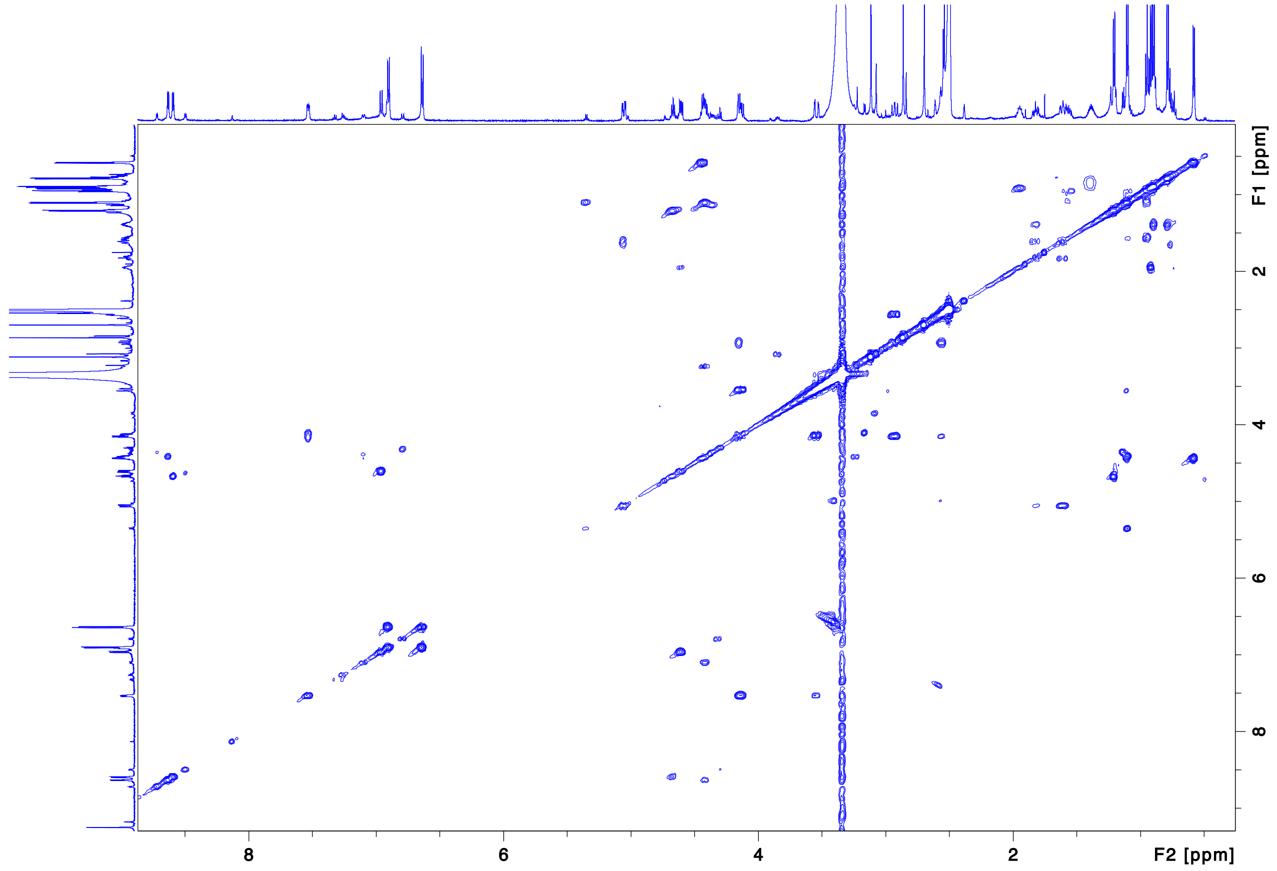

Figure S14. COSY (600 MHz, DMSO-d_6) spectrum of talarolide B (**2**)

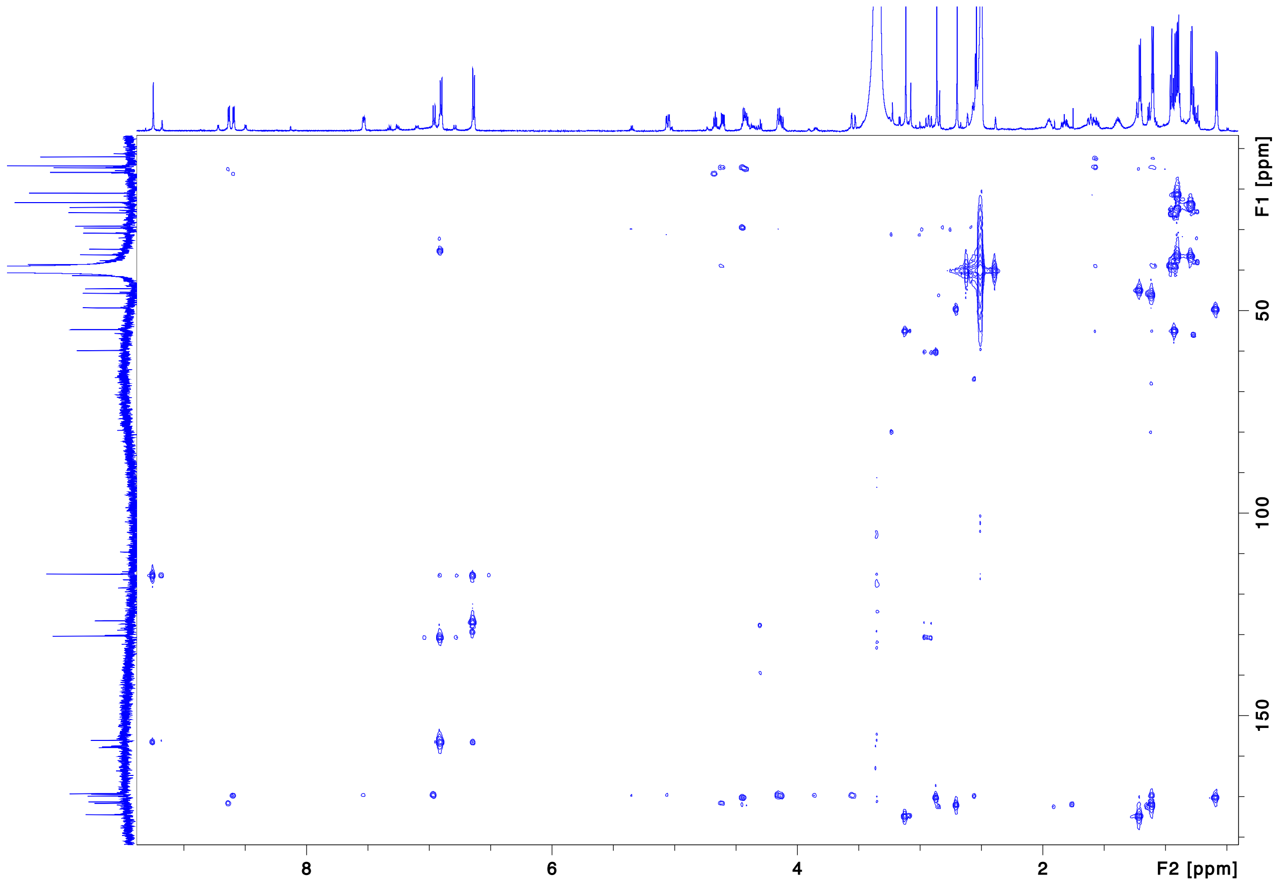

Figure S15. HMBC (600 MHz, DMSO-d_6) spectrum of talarolide B (**2**)

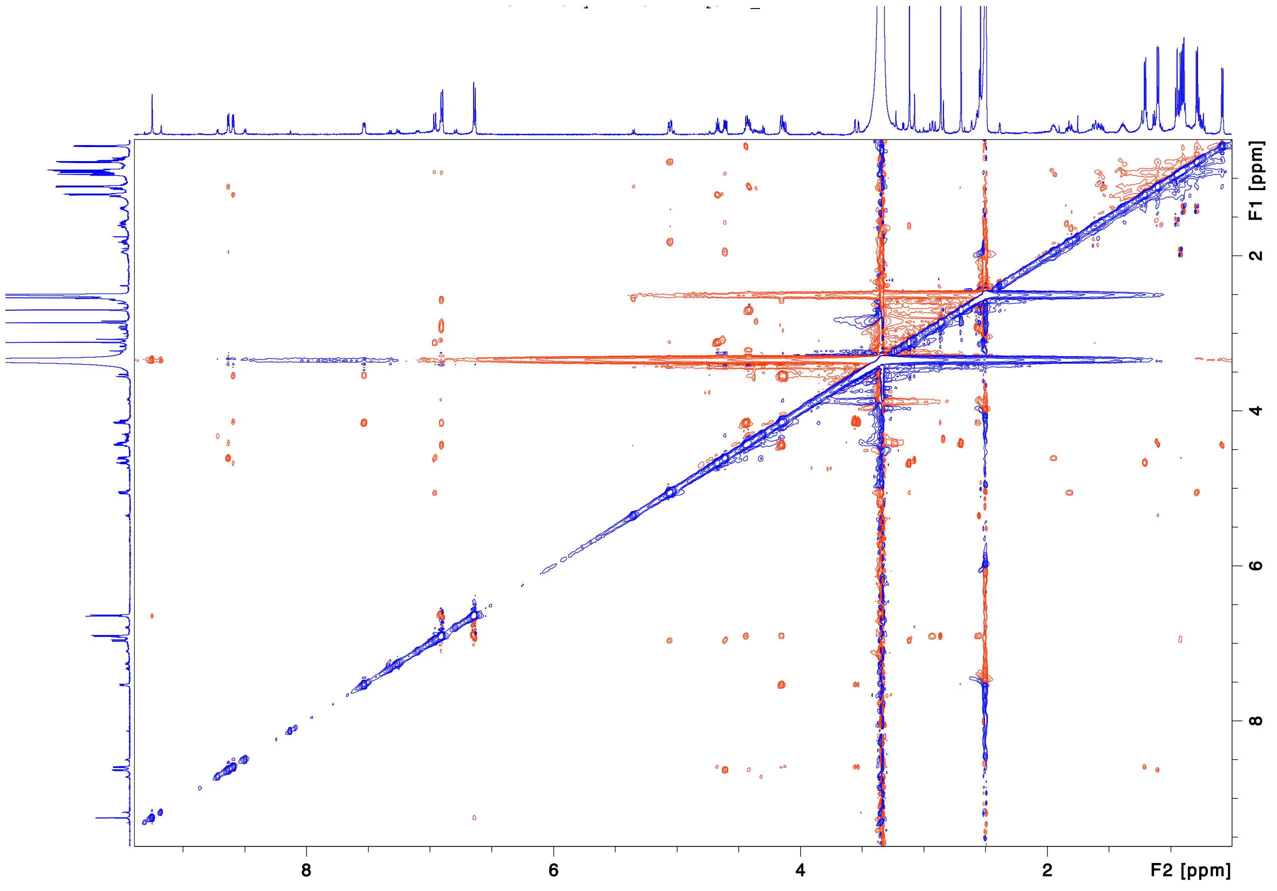

Figure S16. ROESY (600 MHz, DMSO-d_6) spectrum of talarolide B (**2**)

Mass Spectrum Molecular Formula Report

Analysis Info

Analysis Name	D:\Data\a.salim\Talarolide B.d	Acquisition Date	10/21/2022 10:52:27 AM	
Method	tune-med_AP.m	Operator	a.salim	
Sample Name		Instrument / Ser#	micrOTOF	213750.00232
Comment				

Acquisition Parameter

Source Type	ESI	Ion Polarity	Positive	Set Nebulizer	0.8 Bar
Focus	Not active			Set Dry Heater	180 °C
Scan Begin	100 m/z	Set Capillary	4500 V	Set Dry Gas	5.0 l/min
Scan End	1000 m/z	Set End Plate Offset	-500 V	Set Divert Valve	Source

Generate Molecular Formula Parameter

Formula, min.		
Formula, max.		
Measured m/z	Tolerance	Charge
Check Valence	Minimum	Maximum
Nirogen Rule	Electron Configuration	
Filter H/C Ratio	Minimum	Maximum
Estimate Carbon		

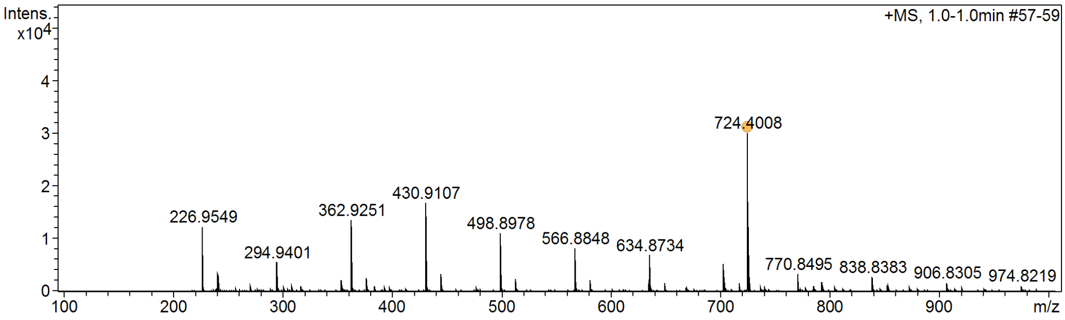

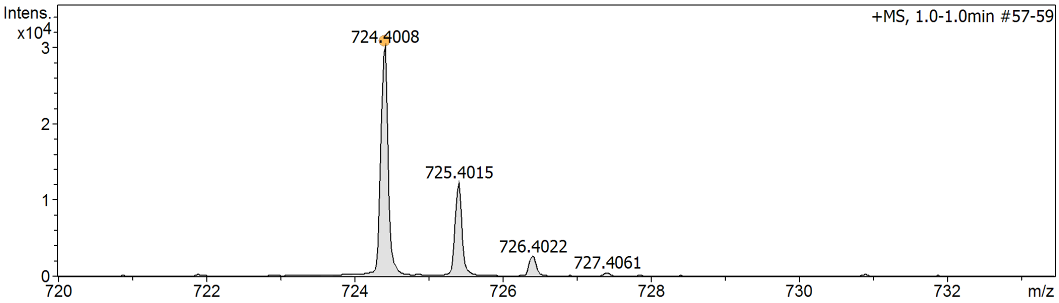

Meas. m/z	#	Ion Formula	m/z	err [ppm]	mSigma	# Sigma	Score	rdb	e⁻ Conf	N-Rule
724.4008	1	C35H55N7NaO8	724.4004	0.5	6.2	1	100.00	11.5	even	ok

Figure S17. HRMS measurement for talarolide B (**2**)

2.3. Talarolide C (3)

Table S4. NMR (DMSO-d_6) data for talarolide C (**3**)

		δ_H, mult, (*J* in Hz)	COSY	HMBC	ROESY
N-OH-Gly[1]					
1	167.3	-	-	-	-
2	50.1	a 4.75[b]	2b	1	*N*-H(L-Ala)
		b 3.76, d (*17.2*)	2a	1, 1(*N*-Me-L-Tyr)	*N*-OH, *N*-H(L-Ala)
N-OH		9.41, s			2b, 2(D-Ala), *N*-Me(*N*-Me-L-Ala), 2(*N*-Me-L-Tyr), 5(D-Val), *N*-H(D-Val)
L-Ala[2]					
1	174.1	-	-	-	-
2	45.2	4.50, qd (*6.7, 4.0*)	3, *N*-H	3	3, *N*-H, *N*-Me(*N*-Me-D-Leu)
3	15.7	1.20, d (*6.7*)	2	1, 2	2, *N*-H
N-H		8.65, d (*4.0*)	2	2, 3, 1(*N*-OH-Gly)	2, 3, 2a(*N*-OH-Gly), 2b(*N*-OH-Gly)
N-Me-D-Leu[3]					
1	169.5	-	-	-	-
2	54.4	5.07, dd (*11.6*, *3.8*)	3a, 3b	1, 3, *N*-Me	3a, 3b, 4, 5, *N*-Me, *N*-H(D-Val)
3	35.9	a 1.79, ddd (*14.4*, *10.3*, *3.8*)	2, 3b, 4	-	2, 5, 6
		b 1.57, ddd (*14.4*, *11.6*, *3.9*)	2, 3a, 4	2, 5	2, 6, *N*-Me
4	24.4	1.38, m	3a, 3b, 5, 6	-	2, 6, *N*-Me
5	20.9	0.78, d (*6.5*)	4	3, 4, 6	2, 3a
6	23.3	0.89, d (*6.5*)	4	3, 4, 5	3a, 3b, 4, *N*-Me
N-Me	31.0	3.01, s	-	2, 1(L-Ala)	2, 3b, 4, 6, 2(L-Ala), *N*-H(D-Val), 4(D-Val), 5(D-Val)
D-Val[4]					
1	171.6	-	-	-	-
2	55.3	4.58, dd (*9.5*, *4.9*)	3, *N*-H	1, 3, 4, 5, 1(*N*-Me-D-Leu)	3, 4, *N*-H, *N*-H(D-Ala)
3	31.9	2.17, m	2, 4, 5	4, 5	2, 4, 5, *N*-H, *N*-H(D-Ala), *N*-OH(*N*-OH-Gly)
4	19.4	0.92, d (*6.8*)	3	2, 3, 5	2, 3, *N*-H, 5/9(*N*-Me-L-Tyr)
5	17.0	0.86, d (*6.8*)	3	2, 3, 4	3, *N*-H, *N*-OH(*N*-OH-Gly), 2(*N*-Me-L-Tyr), *N*-Me(*N*-Me-D-Leu), 5/9(*N*-Me-L-Tyr)
N-H		7.23, d (*9.5*)	2	1, 1(*N*-Me-D-Leu)	2, 3, 4, 5, 2(*N*-Me-D-Leu), *N*-Me(*N*-Me-D-Leu), *N*-OH(*N*-OH-Gly)
D-Ala[5]					
1	171.0	-	-	-	-
2	45.8	4.33, qd (*7.1, 5.0*)	3, *N*-H	1, 3	3, *N*-H, *N*-OH(*N*-OH-Gly), *N*-Me(*N*-Me-L-Ala)
3	14.9	1.12, d (*7.1*)	2	1, 2	2, *N*-H, *N*-Me(*N*-Me-L-Ala)
N-H		8.87, d (*5.0*)	2	2, 3, 1(D-Val)	2, 3, 2(D-Val), 3(D-Val)
N-Me-L-Ala[6]					
1	169.8	-	-	-	-
2	46.7	4.75[b]	3	1, 3, NMe, 1(D-Ala)	3, *N*-Me, 3a(*N*-Me-L-Tyr), 5/9(*N*-Me-L-Tyr), 6/8(*N*-Me-L-Tyr)
3	15.1	0.52, d *(6.5)*	2	1, 2	2, *N*-Me, 5/9(*N*-Me-L-Tyr), 6/8(*N*-Me-L-Tyr)
N-Me	28.6[a]	2.71, s	-	2, 1(D-Ala)	2, 3, 2(D-Ala),3(D-Ala), *N*-OH(*N*-OH-Gly)
N-Me-L-Tyr[7]					
1	168.2	-	-	-	-
2	56.6	4.80, dd (*9.8*, *5.3*)	3a, 3b	1, 3, *N*-Me	3a, 3b, 5/9, *N*-OH(*N*-OH-Gly, 5(D-Val)
3	34.3	a 2.82, dd (*14.2*, *9.8*)	2, 3b	2, 4, 5/9	2, 5/9, 2(*N*-Me-L-Ala)
		b 2.62, dd (*14.2*, *5.3*)	2, 3a	2, 4, 5/9	2, 5/9
4	126.7	-	-	-	-
5/9	130.7	6.94, d (*8.4*)	6/8	3, 7, 9/5	2, 3a, 3b, 6/8, *N*-Me, 2(*N*-Me-L-Ala), 3(*N*-Me-L-Ala), 4(D-Val), 5(D-Val)
6/8	114.8	6.63, d (*8.4*)	5/9	4, 7, 8/6	5/9, 7-OH, 2(*N*-Me-L-Ala), 3(*N*-Me-L-Ala)
7	155.9	-	-	-	-
7-OH		9.18, s	-	6/8, 7	6/8
N-Me	28.6[a]	2.67, s	-	2, 1(*N*-Me-L-Ala)	5/9

[a-b] signals within the same superscripts are overlapping

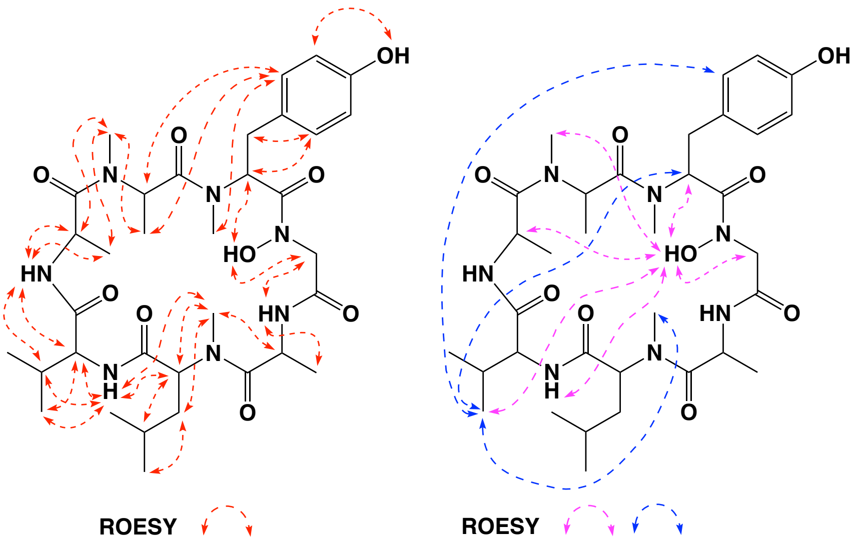

Figure S18. ROESY (DMSO-d_6) correlations for talarolide C (**3**)

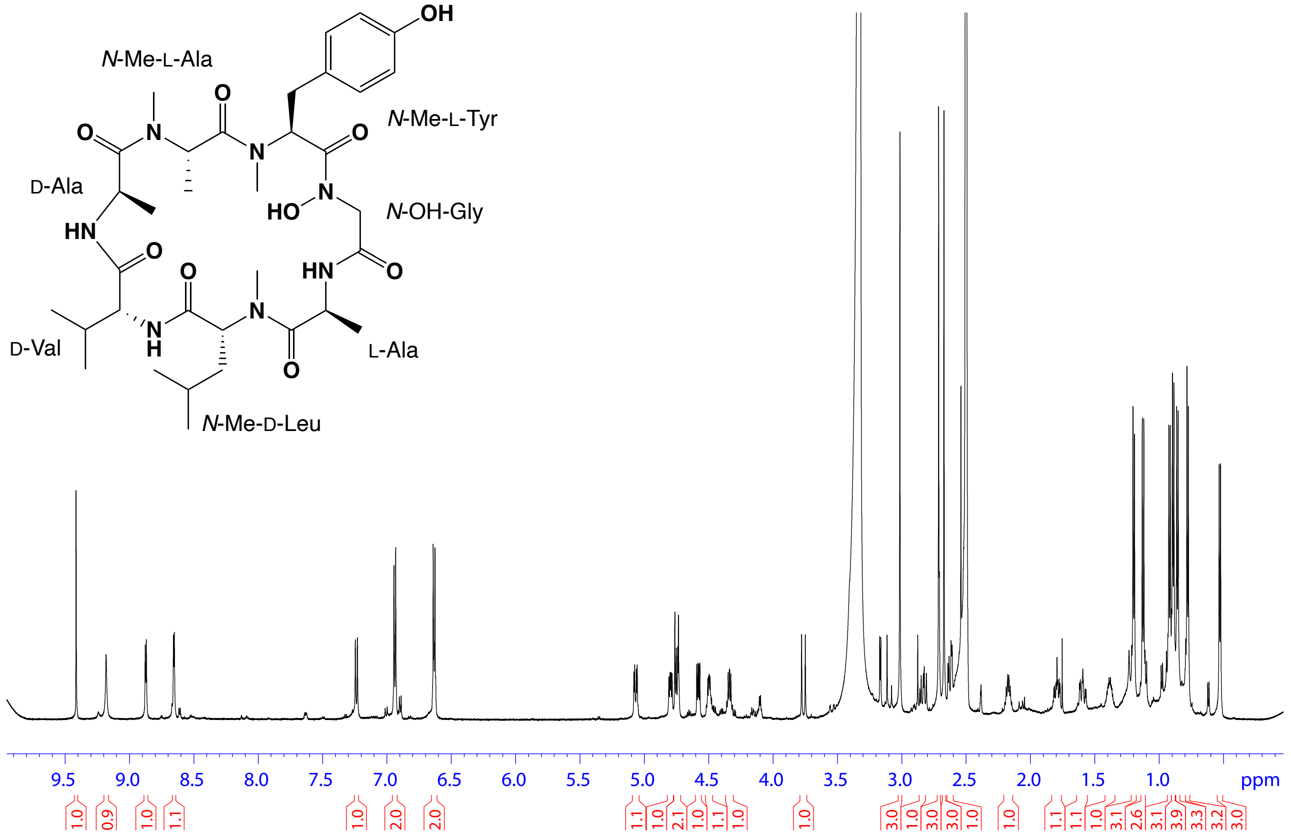

Figure S19. ^{1}H NMR (600 MHz, DMSO-d_6) spectrum of talarolide C (**3**)

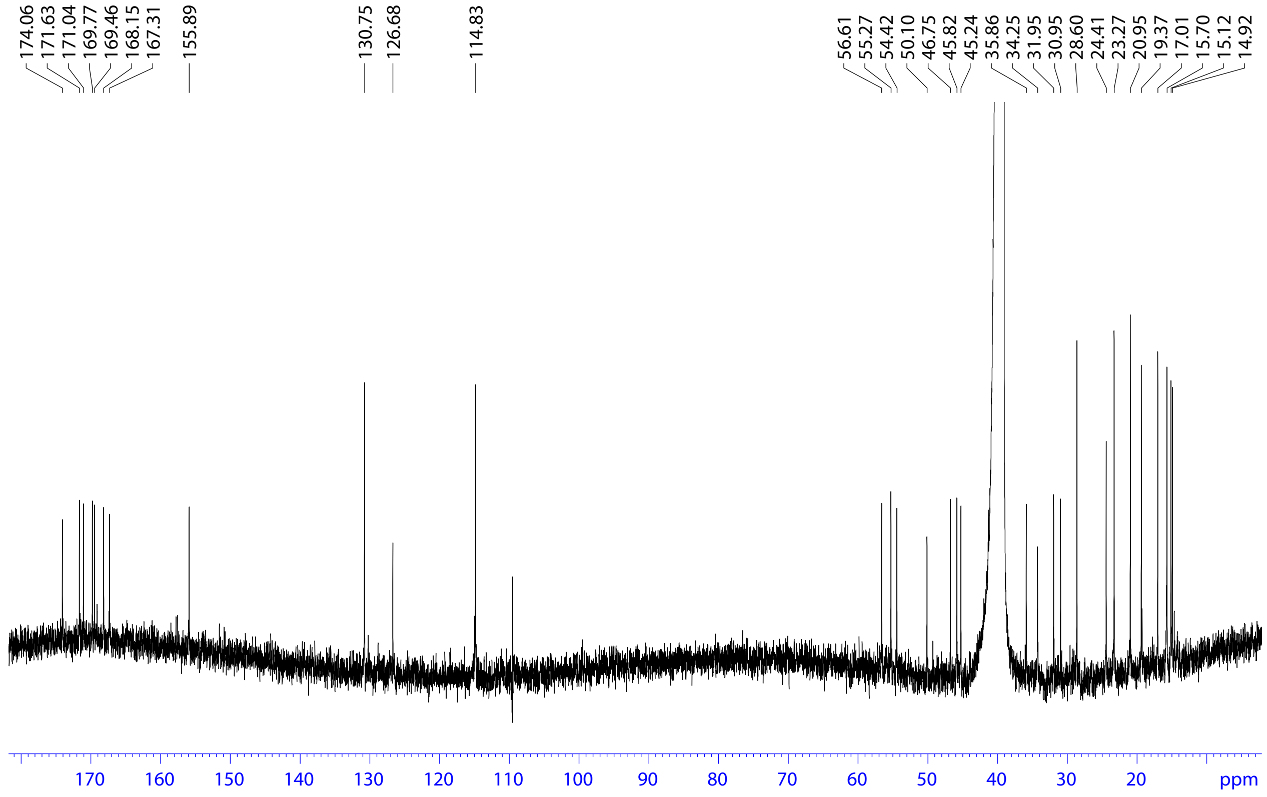

Figure S20. ^{13}C NMR (150 MHz, DMSO-d_6) spectrum of talarolide C (**3**)

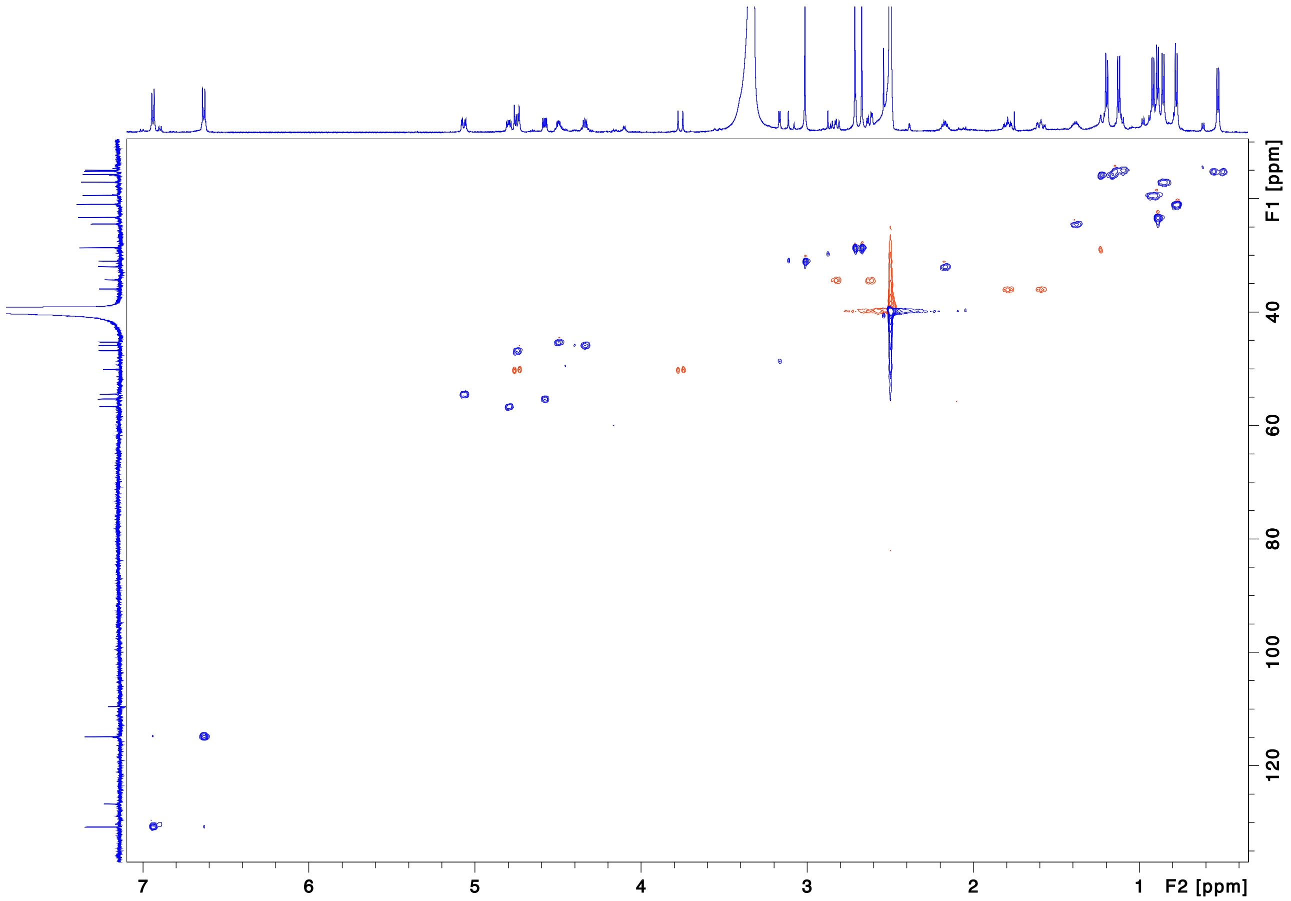

Figure S21. HSQC (600 MHz, DMSO-d_6) spectrum of talarolide C (**3**)

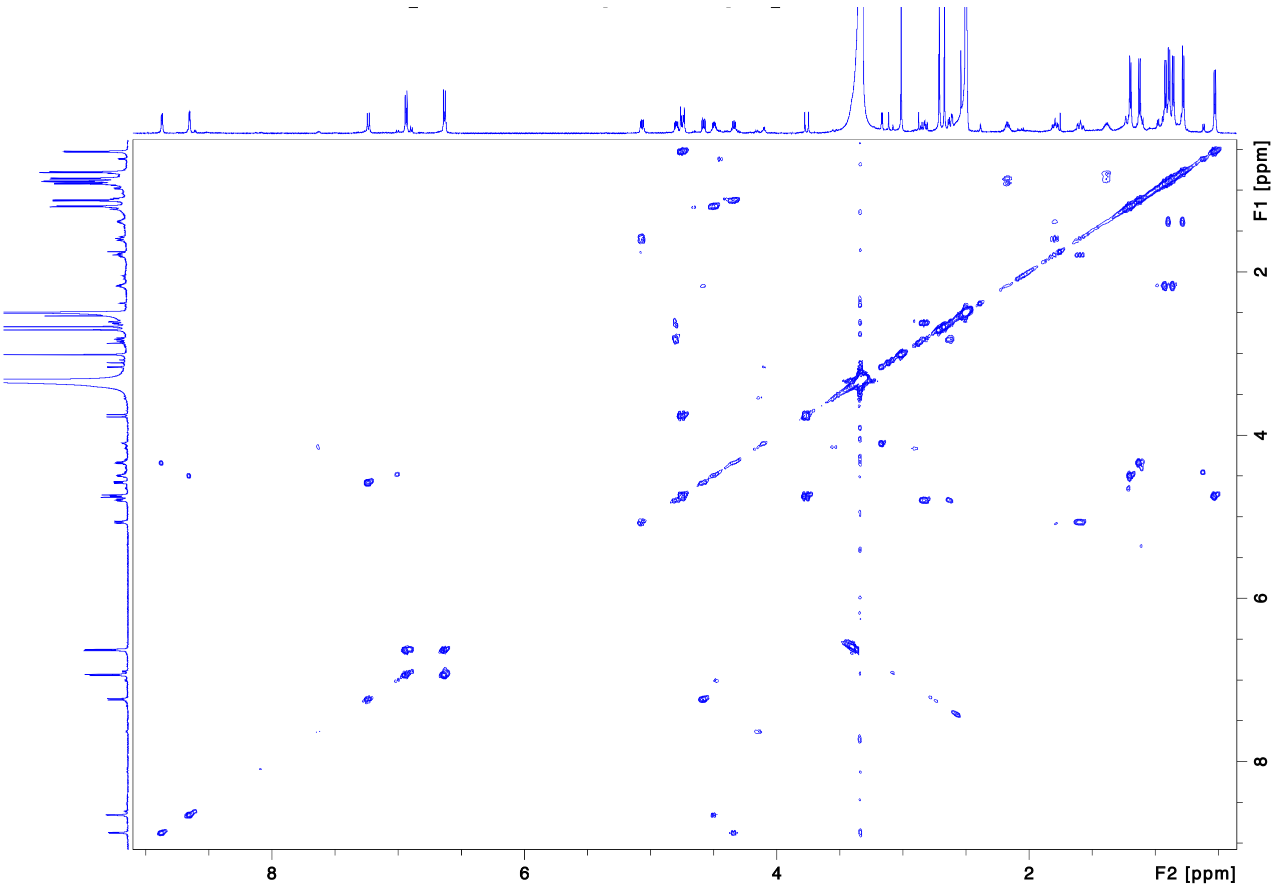

Figure S22. COSY (600 MHz, DMSO-d_6) spectrum of talarolide C (**3**)

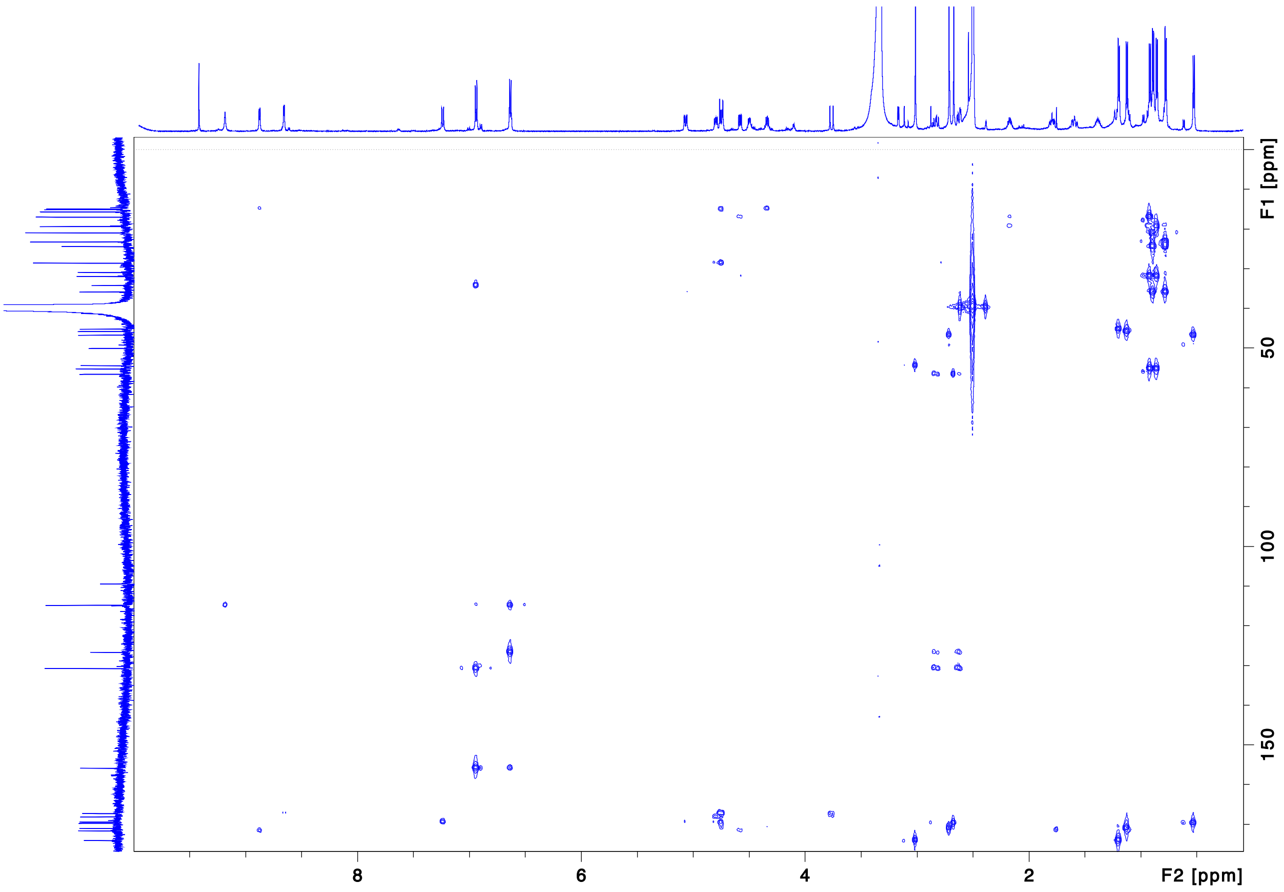

Figure S23. HMBC (600 MHz, DMSO-d_6) spectrum of talarolide C (**3**)

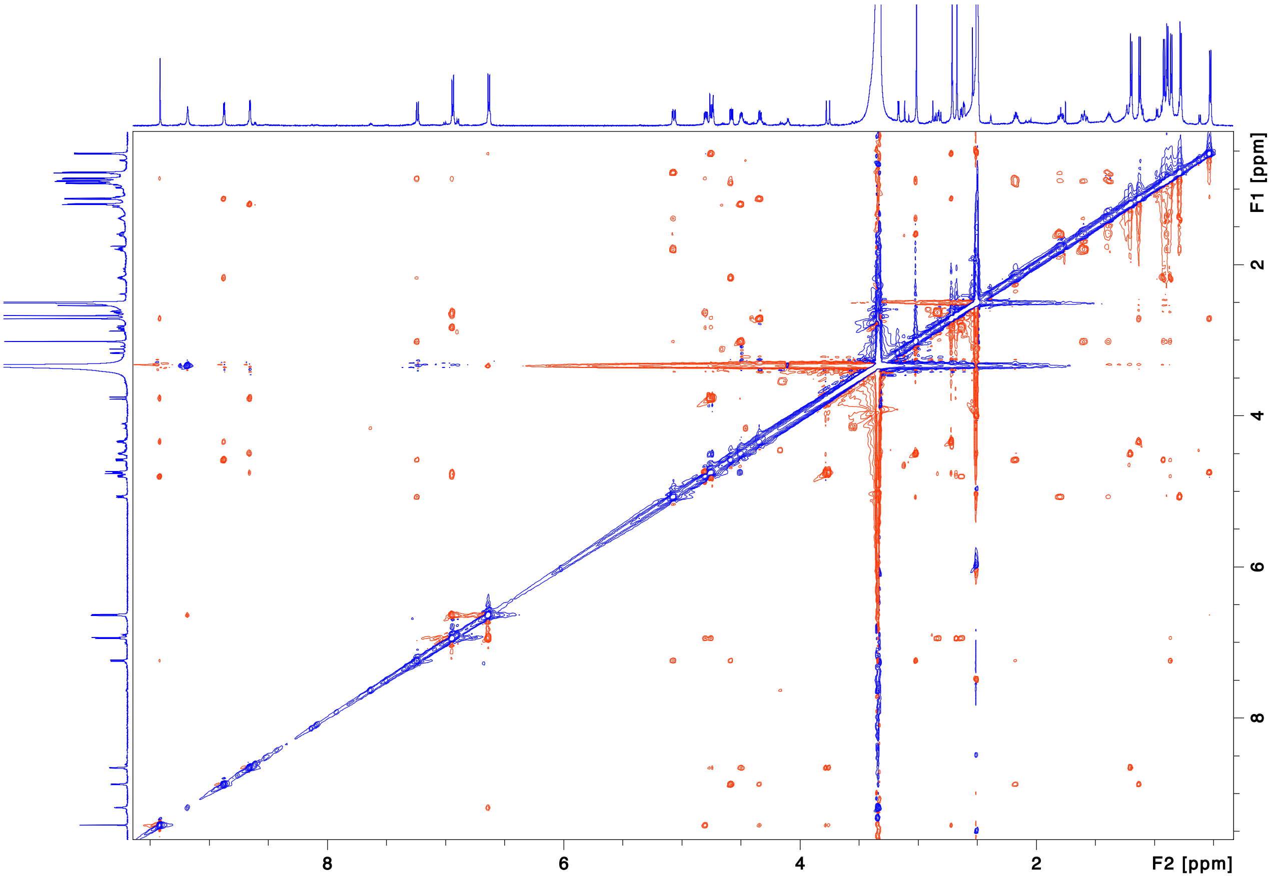

Figure S24. ROESY (600 MHz, DMSO-d_6) spectrum of talarolide C (**3**)

Mass Spectrum Molecular Formula Report

Analysis Info

Analysis Name	D:\Data\a.salim\Talarolide C.d	Acquisition Date	10/21/2022 12:54:25 PM
Method	tune-med_AP.m	Operator	a.salim
Sample Name		Instrument / Ser#	micrOTOF 213750.00232
Comment			

Acquisition Parameter

Source Type	ESI	Ion Polarity	Positive	Set Nebulizer	0.8 Bar
Focus	Not active			Set Dry Heater	180 °C
Scan Begin	100 m/z	Set Capillary	4500 V	Set Dry Gas	5.0 l/min
Scan End	1000 m/z	Set End Plate Offset	-500 V	Set Divert Valve	Source

Generate Molecular Formula Parameter

Formula, min.		
Formula, max.		
Measured m/z	Tolerance	Charge
Check Valence	Minimum	Maximum
Nirogen Rule	Electron Configuration	
Filter H/C Ratio	Minimum	Maximum
Estimate Carbon		

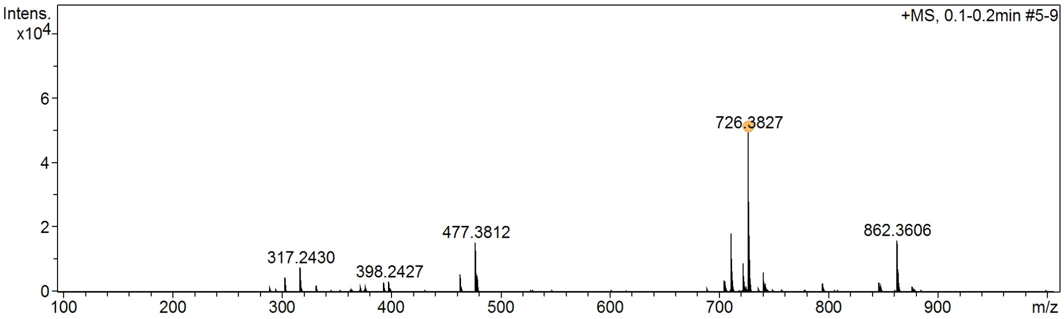

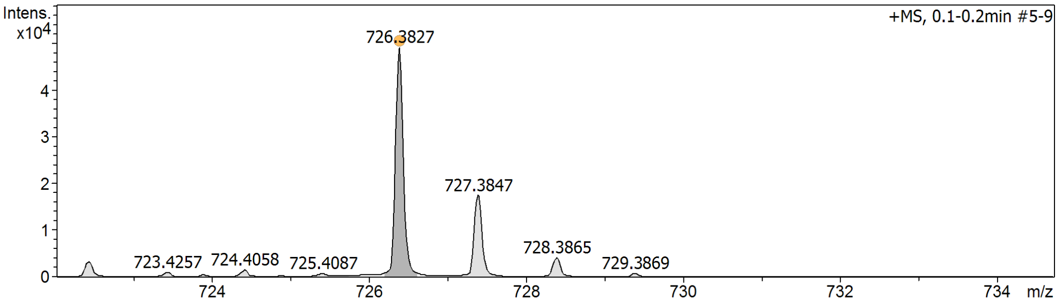

Meas. m/z	#	Ion Formula	m/z	err [ppm]	mSigma	# Sigma	Score	rdb	e¯ Conf	N-Rule
726.3827	1	C34H53N7NaO9	726.3797	4.2	24.9	2	66.03	11.5	even	ok

Figure S25. HRMS measurement for talarolide C (**3**)

2.4. Talarolide D (4)

Table S5. NMR (DMSO-*d*$_6$) data for talarolide D (**4**)

		δ_H, mult, (*J* in Hz)	COSY	HMBC	ROESY
N-OH-Gly[1]					
1	167.5	-	-	-	-
2	49.9	a 4.80 d (*17.0*)	2b	1	*N*-H-(L-Ala[2])
		b 3.71, d (*17.0*)	2a	1	*N*-OH, *N*-H-(L-Ala[2])
N-OH		9.13, s	-	1(*N*-Me-L-Tyr)	2b, 2(D-Ala), 2(*N*-Me-L-Tyr), *N*-H(D-*allo*-Ile), 6(D-*allo*-Ile)
L-Ala[2]					
1	174.0	-	-	-	-
2	45.2	4.49, qd (*6.8, 4.1*)	3, *N*-H	3	3, *N*-H, *N*-Me(*N*-Me-D-Leu)
3	15.7	1.19, d (*6.8*)	2	1, 2	2, *N*-H
N-H		8.62, d (*4.1*)	2	3, 1(*N*-OH-Gly)	2, 3, 2a(*N*-OH-Gly), 2b(*N*-OH-Gly)
N-Me-D-Leu[3]					
1	169.5	-	-	-	-
2	54.4	5.06, dd (*11.7*, *3.8*)	3a, 3b	1, 3, *N*-Me	3a, 4, 5, *N*-Me, *N*-H(D-*allo*-Ile)
3	36.0	a 1.79, ddd (*13.4, 10.6, 3.9*)	2, 3b, 4	-	2, 5, 6
		b 1.57, ddd (*13.4, 11.7, 3.9*)	2, 3a, 4	-	*N*-Me
4	24.4	1.38[a]	3a, 3b, 5, 6	-	2, 5, 6, *N*-Me
5	20.9	0.77, d (*6.5*)	4	3, 4, 6	2, 3a, 4
6	23.3	0.88, d (*6.6*)	4	3, 4, 5	4
N-Me	30.9	2.98, s	-	2, 1(L-Ala[1])	2, 3b, 4, 2(L-Ala[2]), *N*-H(D-*allo*-Ile), 6(D-*allo*-Ile)
D-*allo*-Ile[4]					
1	171.8	-	-	-	-
2	53.8	4.69, dd (*9.5*, *3.9*)	3, *N*-H	1, 3, 6	3, 4a, 5, 6, *N*-H, *N*-H(D-Ala)
3	38.8	1.91, m	2, 4a, 4b, 6	-	2, 5, *N*-H, *N*-H(D-Ala)
4	26.4	a 1.39[a]	3, 4b, 5	2, 3, 5, 6	2, 6, *N*-H
		b 1.05, m	3, 4a, 5	2, 3, 5, 6	6, *N*-H, *N*-Me(*N*-Me-D-Leu)
5	11.9	0.92, dd (*7.3, 7.3*)	4a, 4b	3, 4	2, 3, 5/9(*N*-Me-L-Tyr)
6	13.7	0.80, d (*6.9*)	3	2, 3, 4	2, 4a, 4b, *N*-H, *N*-Me(*N*-Me-D-Leu), 5/9(*N*-Me-L-Tyr), 6/8(*N*-Me-L-Tyr), *N*-OH(*N*-OH-Gly)
N-H		7.14, d (*9.6*)	2	1(*N*-Me-D-Leu)	2, 3, 4a, 4b, 6, 2(*N*-Me-D-Leu), *N*-Me(*N*-Me-D-Leu), *N*-OH(*N*-OH-Gly, 2(L-Ala[1])
D-Ala[5]					
1	170.8	-	-	-	-
2	48.6	3.90, m	3, *N*-H	1, 3	3, *N*-H, *N*-H(L-Ala[6]), *N*-OH(*N*-OH-Gly)
3	16.7	1.13, d (*7.1*)	2	1, 2	2, *N*-H, *N*-H(L-Ala[6])
N-H		8.58, d (*5.2*)	2	3, 1(D-*allo*-Ile)	2, 3, 2(D-*allo*-Ile), 3(D-*allo*-Ile)
L-Ala[6]					
1	171.4	-	-	-	-
2	42.7	4.37, m	3, *N*-H	3	3, *N*-H, 2(*N*-Me-L-Tyr), 5/9(*N*-Me-L-Tyr)
3	18.5	0.63, d *(6.4)*	2	1, 2	2, *N*-H, 5/9(*N*-Me-L-Tyr), 6/8(*N*-Me-L-Tyr)
N-H		8.27, d *(9.5)*	2	3, 1(D-Ala)	2, 3, 2(D-Ala), 3(D-Ala)
N-Me-L-Tyr[7]					
1	168.3	-		-	-
2	57.1	5.01, dd (*8.6, 6.6*)	3a, 3b	1, 3, 4, *N*-Me, 1(L-Ala[6])	5/9, *N*-OH(*N*-OH-Gly), 2(L-Ala[6]), 6(D-*allo*-Ile)
3	34.5	a 2.81, dd (*14.3, 6.6*)	2, 3b	1, 2, 4, 5/9	5/9
		b 2.77, dd (*14.3, 8.6*)	2, 3a	1, 2, 4, 5/9	5/9
4	127.2	-	-	-	-
5/9	130.6	6.99, d (*8.4*)	6, 8	3, 7, 6/8, 9/5	2, 3a, 3b, 6/8, *N*-Me, 2(L-Ala[6]), 3(L-Ala[6]), 5(D-*allo*-Ile), 6(D-*allo*-Ile)
6/8	114.9	6.63, d (*8.4*)	5, 9	4, 7, 8/6	5/9, 7-OH, 3(L-Ala[6]), 6(D-*allo*-Ile)
7	155.8	-	-	-	-
7-OH		9.15, s	-	6/8, 7	6/8
N-Me	28.6	2.64, s	-	2, 1(L-Ala[6])	5/9

[a] signals are overlapping

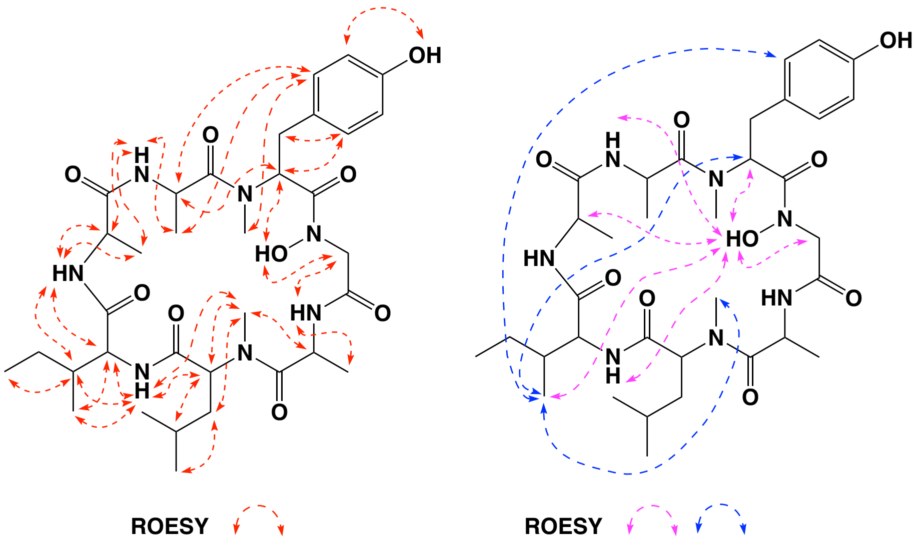

Figure S26. ROESY (DMSO-d_6) correlations for talarolide D (**4**)

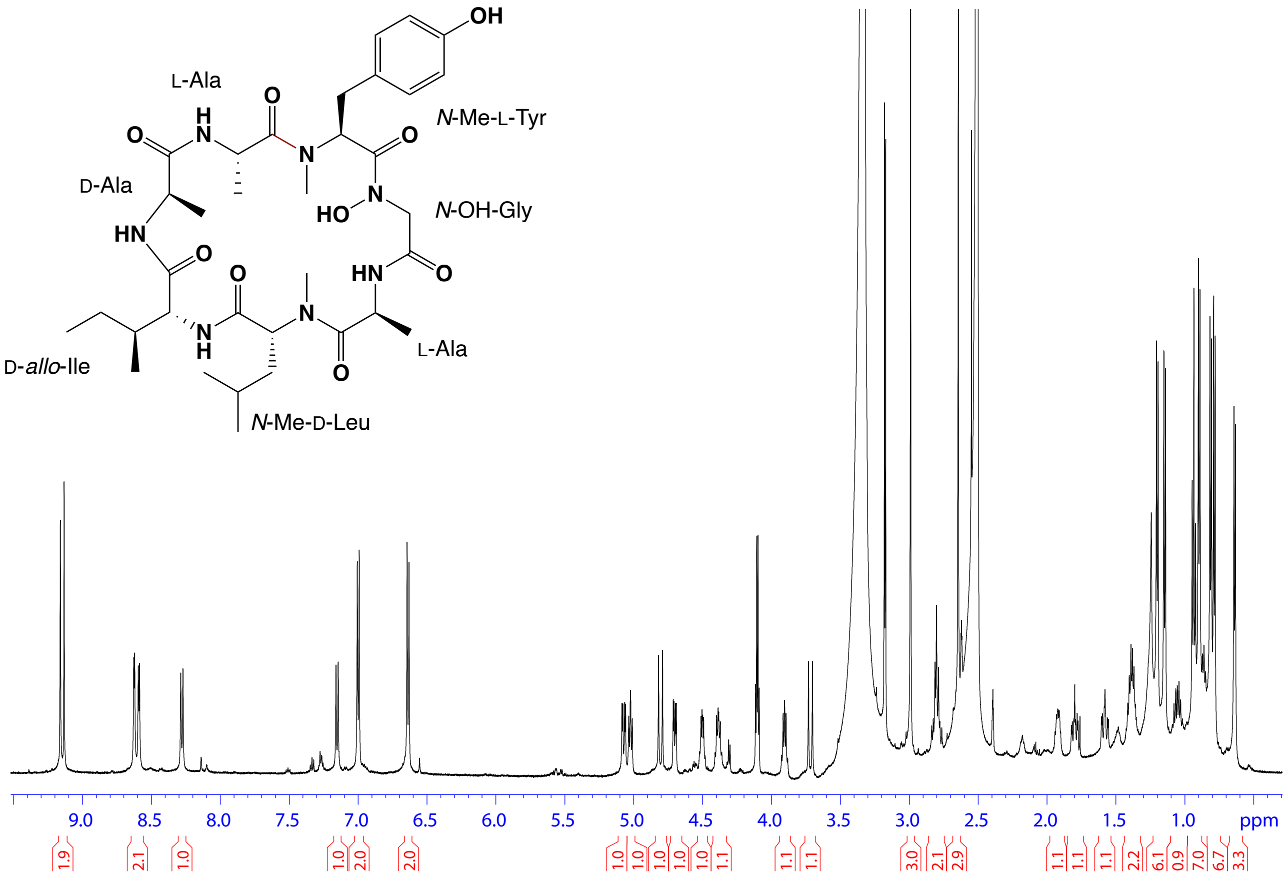

Figure S27. ^{1}H NMR (600 MHz, DMSO-d_6) spectrum of talarolide D (**4**)

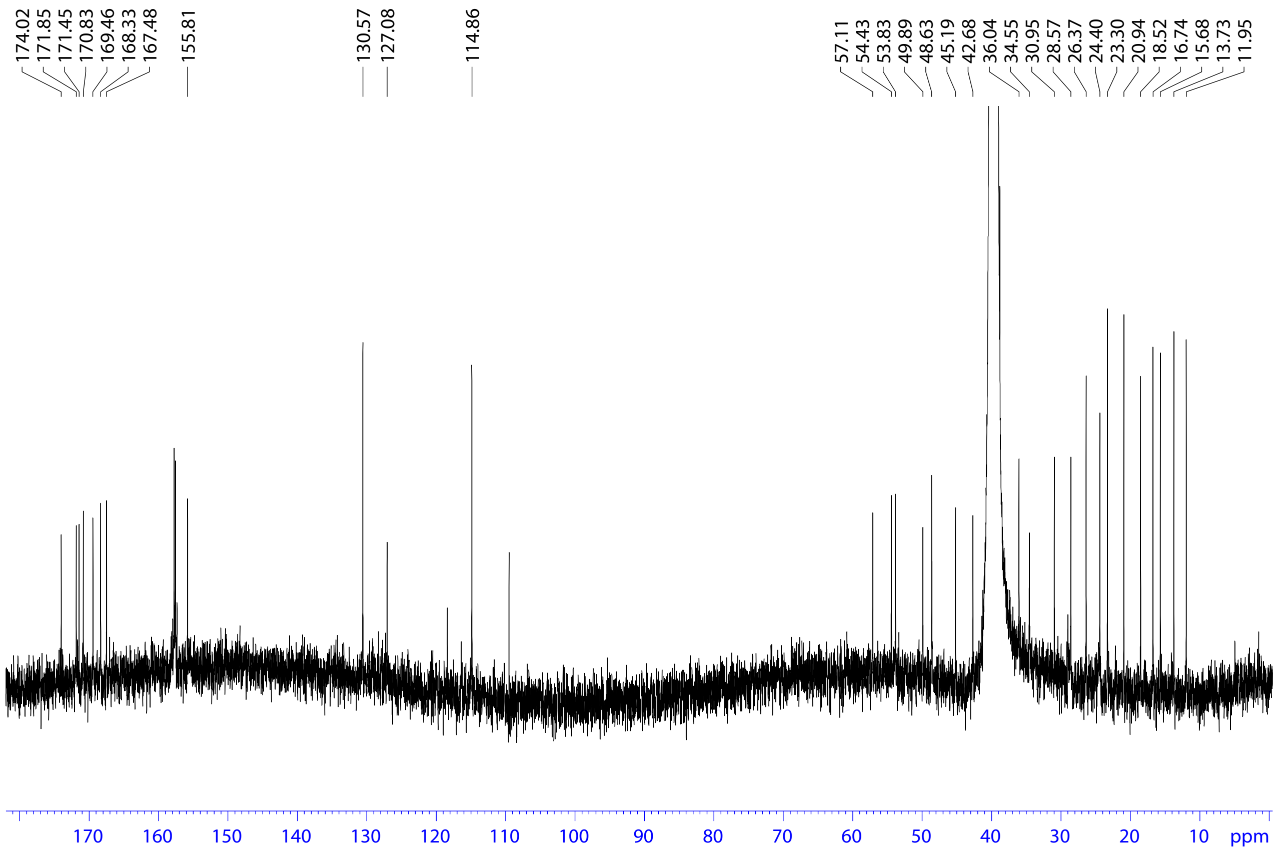

Figure S28. ^{13}C NMR (150 MHz, DMSO-d_6) spectrum of talarolide D (**4**)

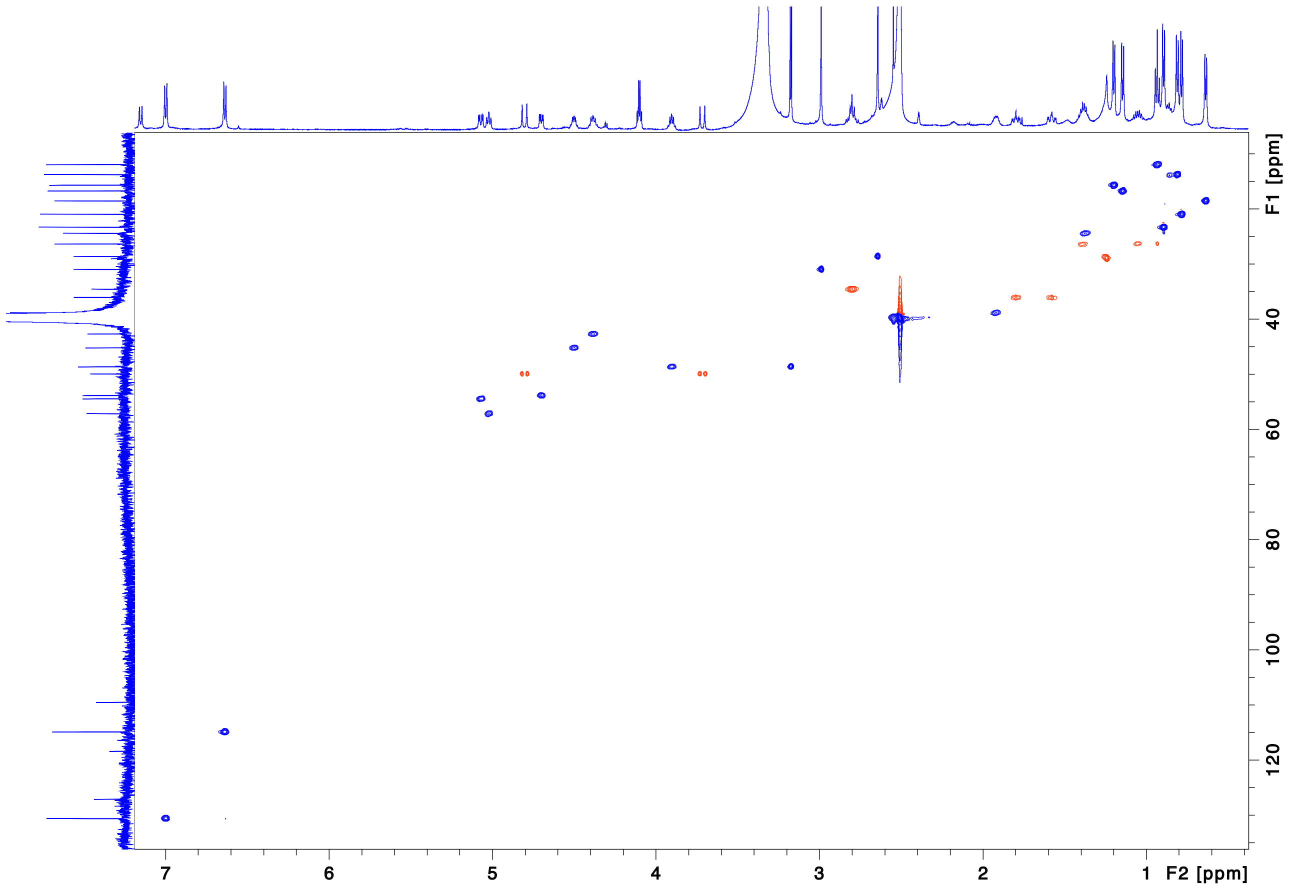

Figure S29. HSQC (600 MHz, DMSO-d_6) spectrum of talarolide D (**4**)

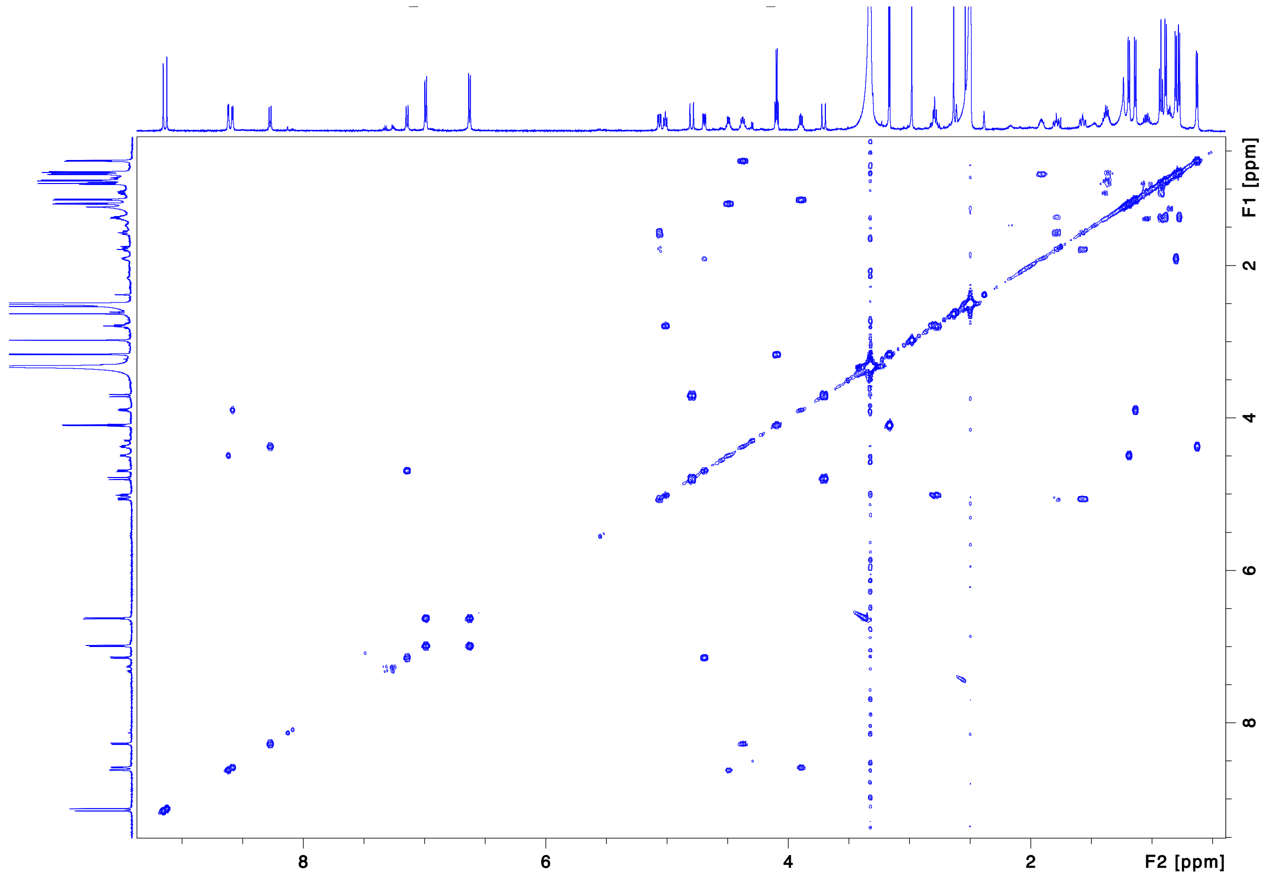

Figure S30. COSY (600 MHz, DMSO-d_6) spectrum of talarolide D (**4**)

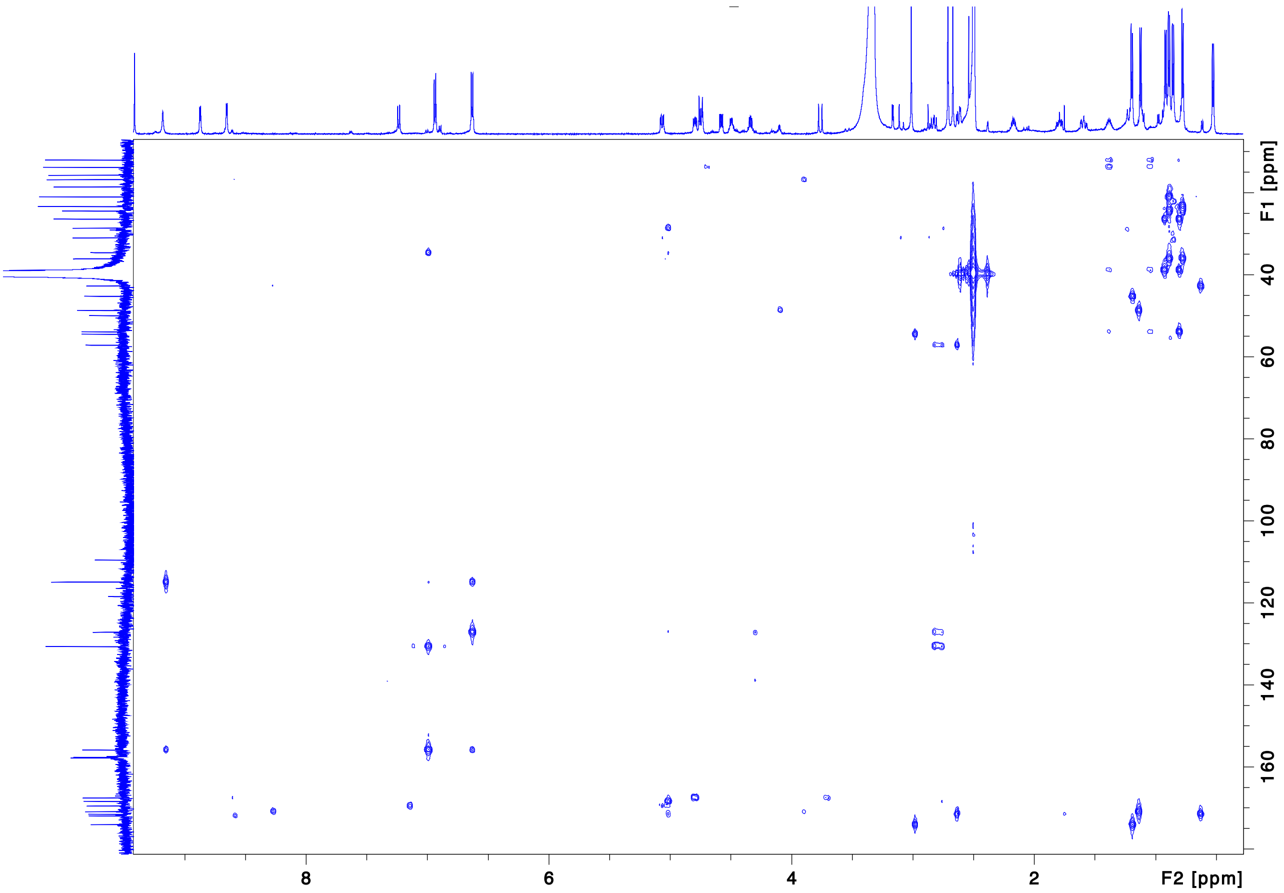

Figure S31. HMBC (600 MHz, DMSO-d_6) spectrum of talarolide D (**4**)

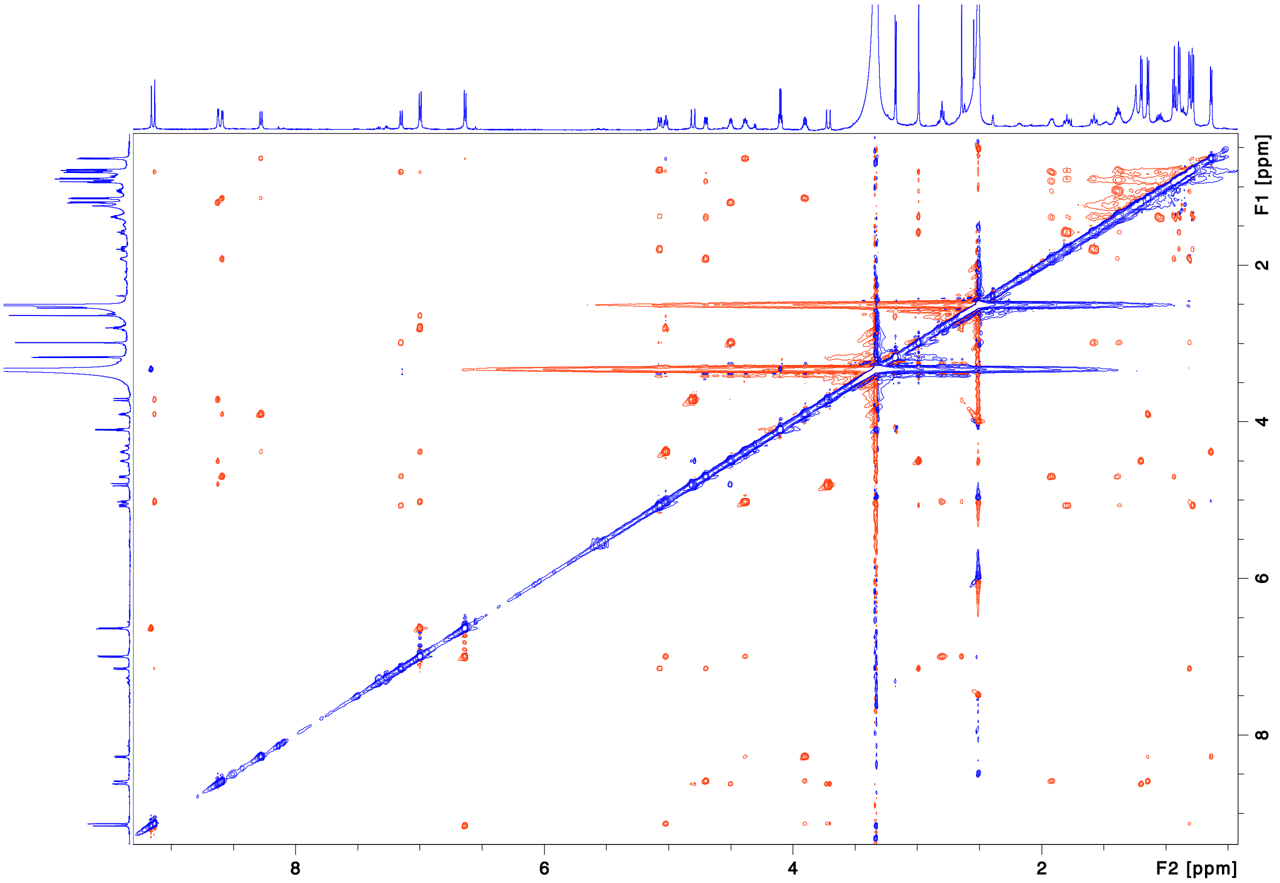

Figure S32. ROESY (600 MHz, DMSO-d_6) spectrum of talarolide D (**4**)

Mass Spectrum Molecular Formula Report

Analysis Info

Analysis Name	D:\Data\a.salim\Talarolide D.d	Acquisition Date	10/21/2022 1:24:48 PM
Method	tune-med_AP.m	Operator	a.salim
Sample Name		Instrument / Ser#	micrOTOF 213750.00232
Comment			

Acquisition Parameter

Source Type	ESI	Ion Polarity	Positive	Set Nebulizer	0.8 Bar
Focus	Not active			Set Dry Heater	180 °C
Scan Begin	100 m/z	Set Capillary	4500 V	Set Dry Gas	5.0 l/min
Scan End	1000 m/z	Set End Plate Offset	-500 V	Set Divert Valve	Source

Generate Molecular Formula Parameter

Formula, min.		
Formula, max.		
Measured m/z	Tolerance	Charge
Check Valence	Minimum	Maximum
Nirogen Rule	Electron Configuration	
Filter H/C Ratio	Minimum	Maximum
Estimate Carbon		

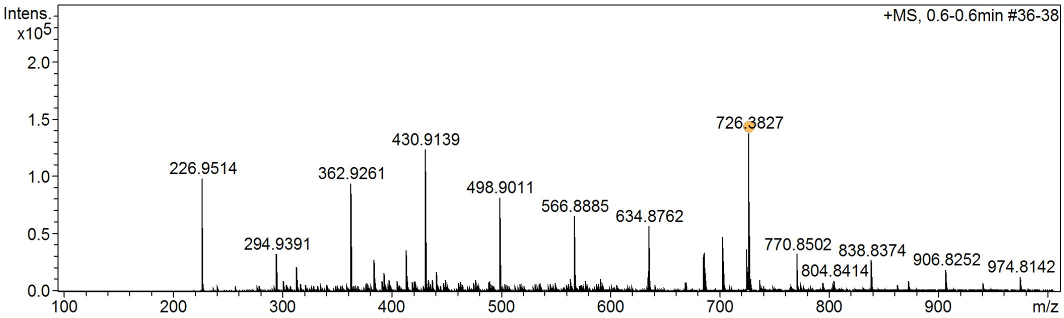

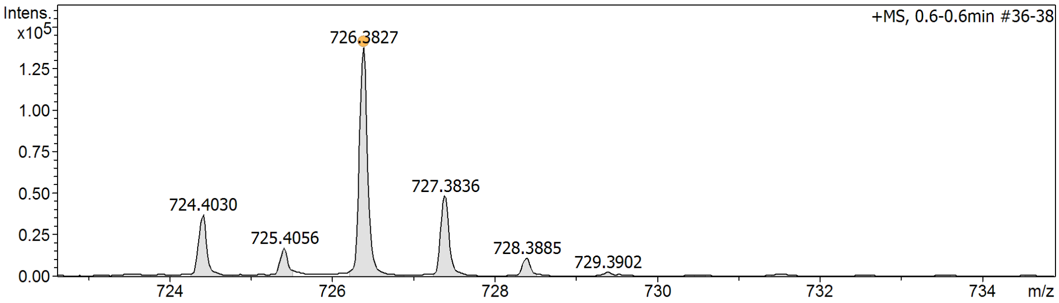

Meas. m/z	#	Ion Formula	m/z	err [ppm]	mSigma	# Sigma	Score	rdb	e⁻ Conf	N-Rule
726.3827	1	C34H53N7NaO9	726.3797	-4.2	27.6	2	57.26	11.5	even	ok

Figure S33. HRMS measurement for talarolide D (**4**)

3. Marfey's analysis of talarolides A-D

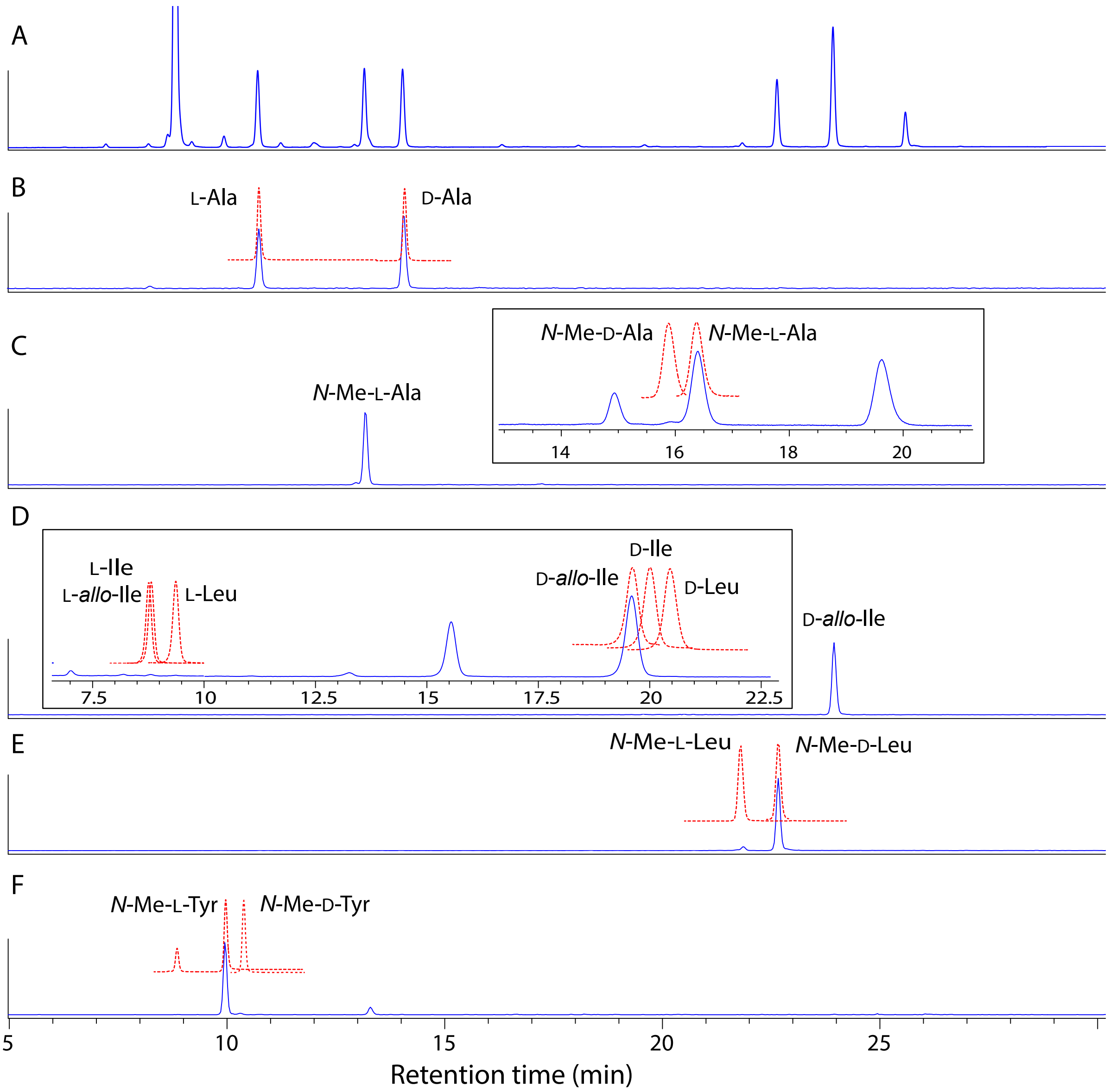

Figure S34. Marfeys analysis of talarolide A (**1**). (A) HPLC-DAD (340 nm) chromatogram showing L-FDAA amino acid derivatives of the acid hydrolysate **1**. (B-F) HPLC-MS(+)-SIE (single ion extraction) chromatograms for L-FDAA derivatives of authentic amino acid standards (red traces) and the acid hydrolysate of **1** (blue traces). The insets in (C) and (D) showed the UPLC-DAD (340 nm) chromatograms. Traces confirm that **1** incorporates (B) L-Ala and D-Ala (SIE *m/z* 342), (C) *N*-Me-L-Ala (SIE *m/z* 356), (D) D-*allo*-Ile (SIE *m/z* 384), (E) *N*-Me-D-Leu (SIE *m/z* 398), and (F) *N*-Me-L-Tyr (SIE *m/z* 448).

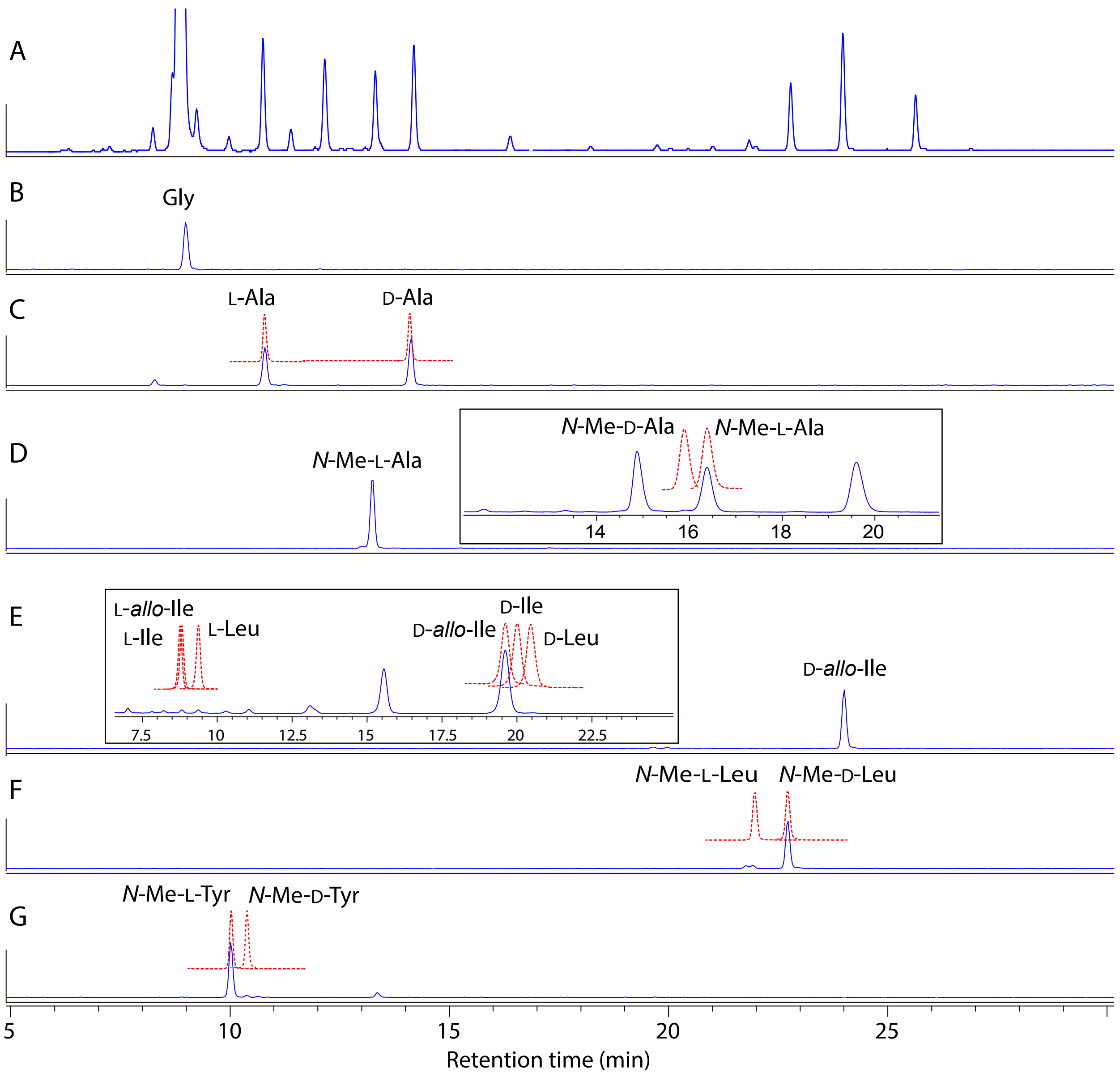

Figure S35. Marfeys analysis of talarolide B (**2**). (A) HPLC-DAD (340 nm) chromatogram showing L-FDAA amino acid derivatives of the acid hydrolysate **2**. (B-G) HPLC-MS(+)-SIE (single ion extraction) chromatograms for L-FDAA derivatives of authentic amino acid standards (red traces) and the acid hydrolysate of **2** (blue traces). The insets in (D) and (E) showed the UPLC-DAD (340 nm) chromatograms. Traces confirm that **2** incorporates (B) Gly (SIE *m/z* 328), (C) L-Ala and D-Ala (SIE *m/z* 342), (D) *N*-Me-L-Ala (SIE *m/z* 356), (E) D-*allo*-Ile (SIE *m/z* 384), (F) *N*-Me-D-Leu (SIE *m/z* 398), and (G) *N*-Me-L-Tyr (SIE *m/z* 448).

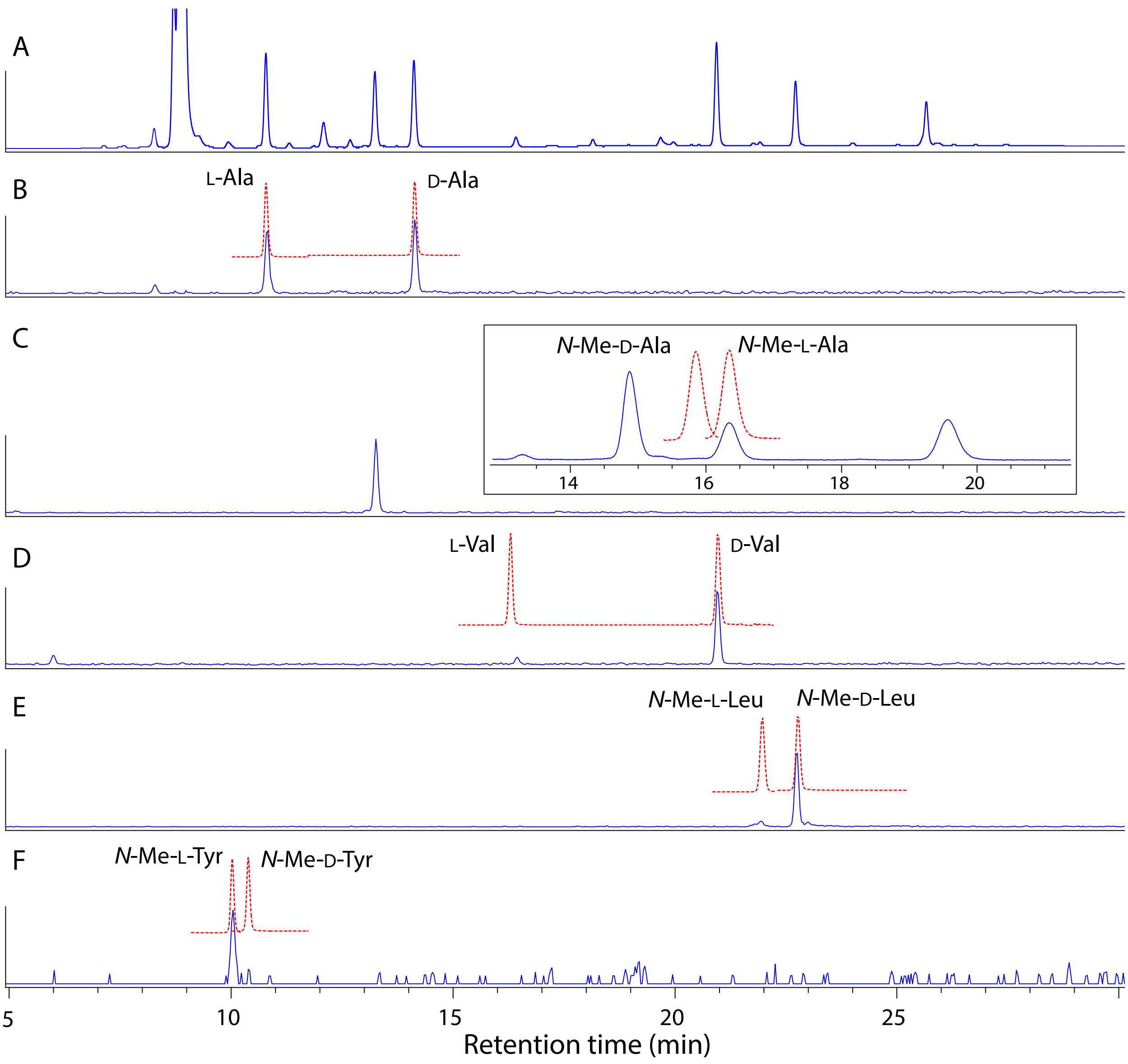

Figure S36. Marfeys analysis of talarolide C (**3**). (A) HPLC-DAD (340 nm) chromatogram showing L-FDAA amino acid derivatives of the acid hydrolysate **3**. (B-F) HPLC-MS(+)-SIE (single ion extraction) chromatograms for L-FDAA derivatives of authentic amino acid standards (red traces) and the acid hydrolysate of **3** (blue traces). The inset in (C) showed the UPLC-DAD (340 nm) chromatogram. Traces confirm that **3** incorporates (B) L-Ala and D-Ala (SIE *m/z* 342), (C) *N*-Me-L-Ala (SIE *m/z* 356), (D) D-Val (SIE *m/z* 370), (E) *N*-Me-D-Leu (SIE *m/z* 398), and (F) *N*-Me-L-Tyr (SIE *m/z* 448).

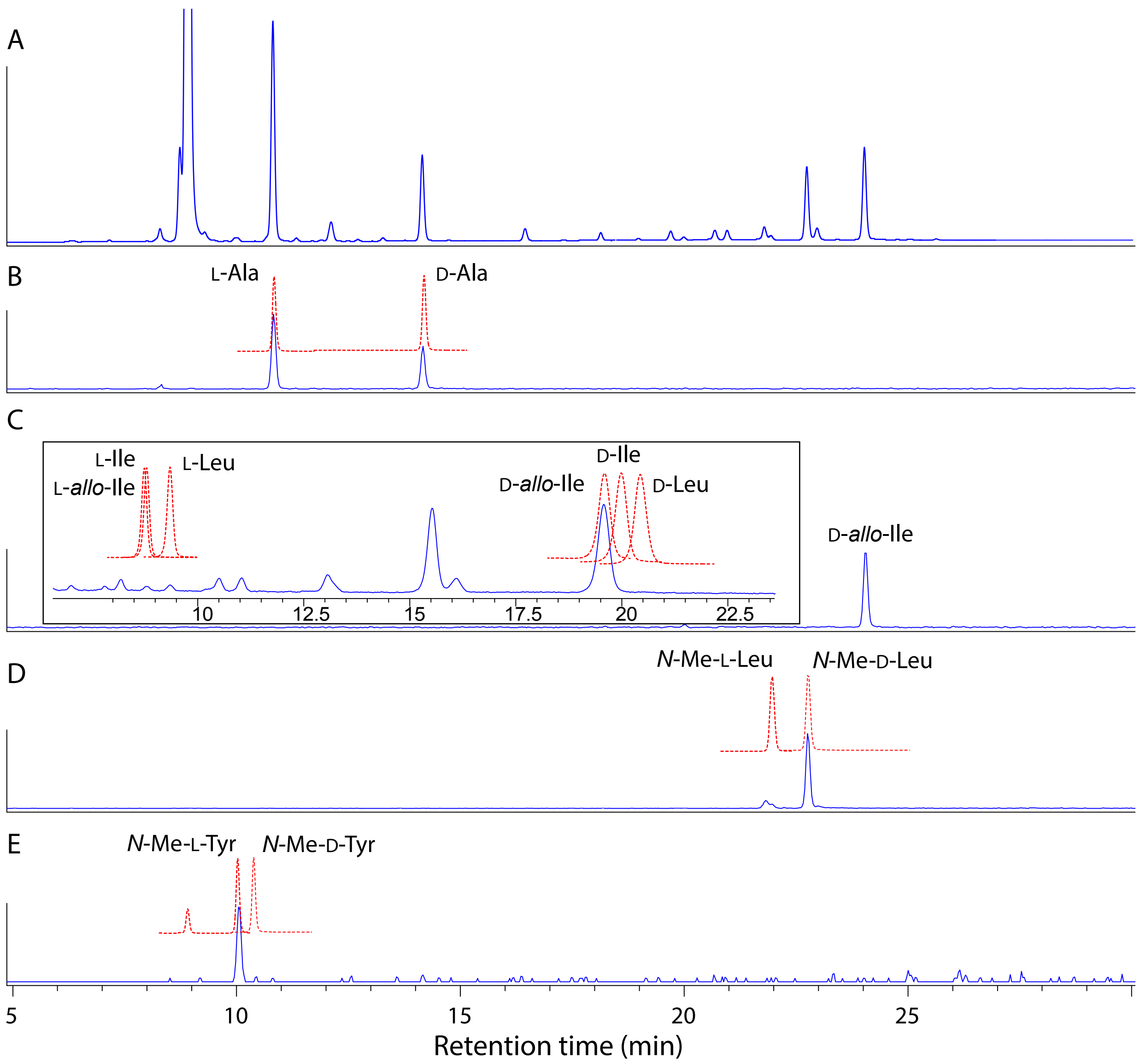

Figure S37. Marfeys analysis of talarolide D (**4**). (A) HPLC-DAD (340 nm) chromatogram showing L-FDAA amino acid derivatives of the acid hydrolysate **4**. (B-F) HPLC-MS(+)-SIE (single ion extraction) chromatograms for L-FDAA derivatives of authentic amino acid standards (red traces) and the acid hydrolysate of **4** (blue traces). The insets in (C) showed the UPLC-DAD (340 nm) chromatogram. Traces confirm that **4** incorporates (B) L-Ala and D-Ala (SIE *m/z* 342), (C) D-*allo*-Ile (SIE *m/z* 384), (D) *N*-Me-D-Leu (SIE *m/z* 398), and (E) *N*-Me-L-Tyr (SIE *m/z* 448).

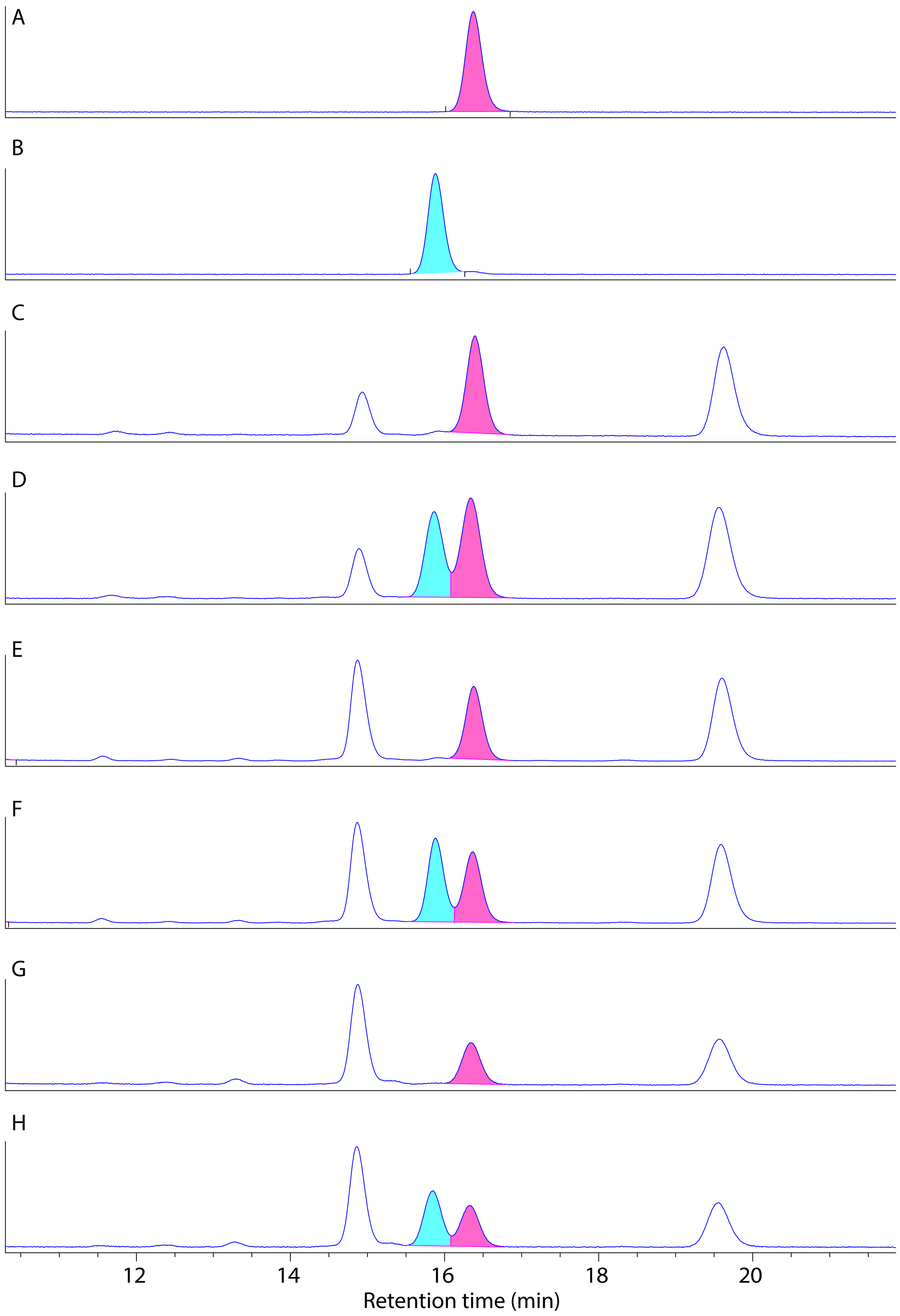

Figure S38. UPLC-DAD (340 nm) chromatograms showing (A) synthetic L-FDAA-*N*-Me-L-Ala; (B) synthetic L-FDAA-*N*-Me-D-Ala; (C) acid hydrolysate of **1**, (D) acid hydrolysate of **1** co-injected with synthetic L-FDAA-*N*-Me-D-Ala; (E) acid hydrolysate of **2**; (F) acid hydrolysate of **2** co-injected with synthetic L-FDAA-*N*-Me-D-Ala; (G) acid hydrolysate of **3**; (H) acid hydrolysate of **3** co-injected with synthetic L-FDAA-*N*-Me-D-Ala, confirming the presence of *N*-Me-L-Ala in **1-3**.

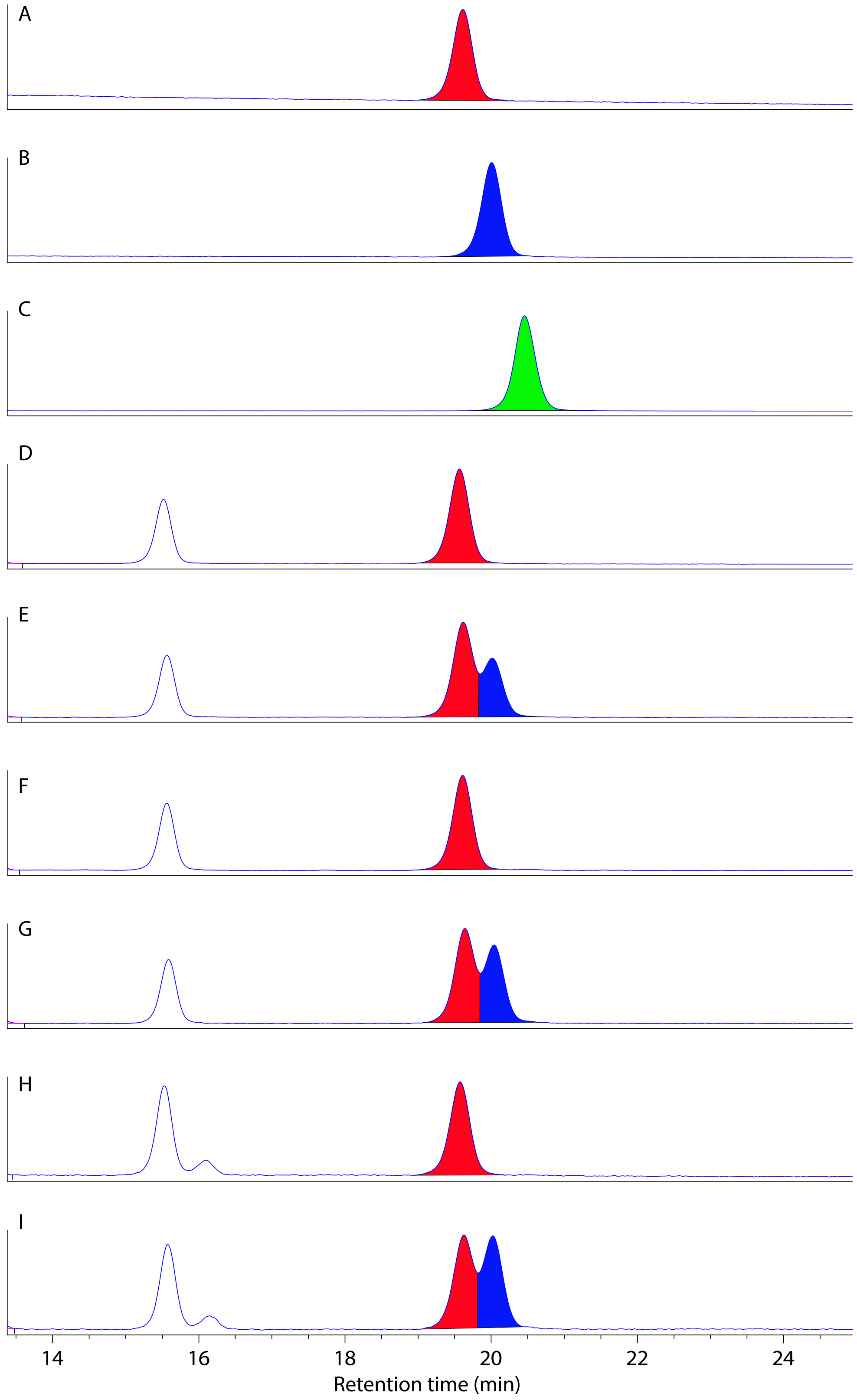

Figure S39. UPLC-DAD (340 nm) chromatograms showing: (A) synthetic L-FDAA-D-*allo*-Ile; (B) synthetic L-FDAA-D-Ile; (C) synthetic L-FDAA-D-Leu; (D) acid hydrolysate of **1**; (E) acid hydrolysate of **1** co-injected wtih L-FDAA-D-Ile; (F) acid hydrolysate of **2**; (G) acid hydrolysate of **2** co-injected wtih L-FDAA-D-Ile; (H) acid hydrolysate of **4**; (E) acid hydrolysate of **4** co-injected wtih L-FDAA-D-Ile; confirming the presence of D-*allo*-Ile in **1**, **2** and **4**.

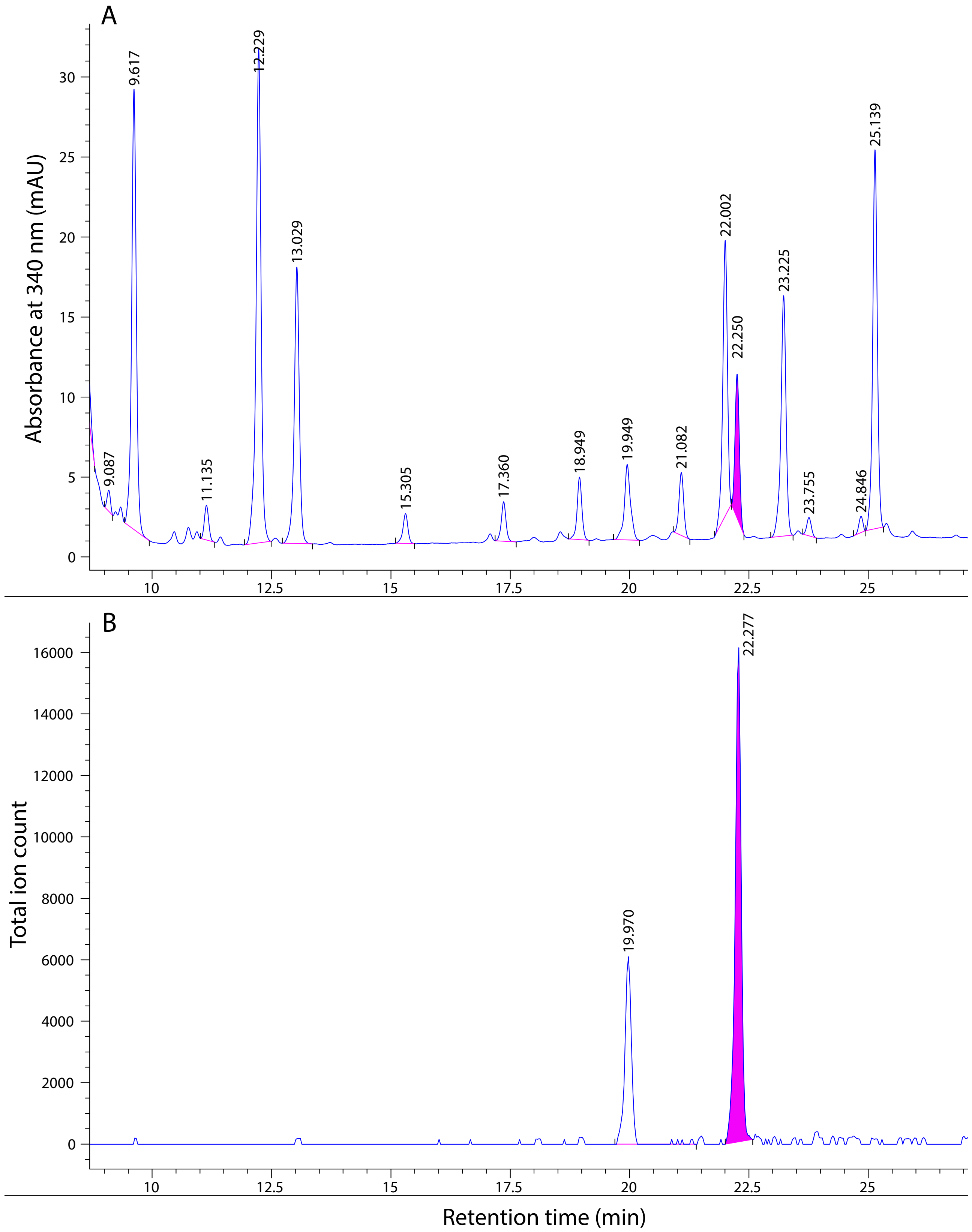

Figure 40. (A) HPLC-DAD (340 nm) chromatogram showing L-FDAA derivatives of partial hydrolysate of **2** and (B) HPLC-MS(+)-SIE (single ion extraction) chromatogram for L-FDAA-D-*allo*-Ile-Ala (*m/z* 455) (peak shaded in magenta).

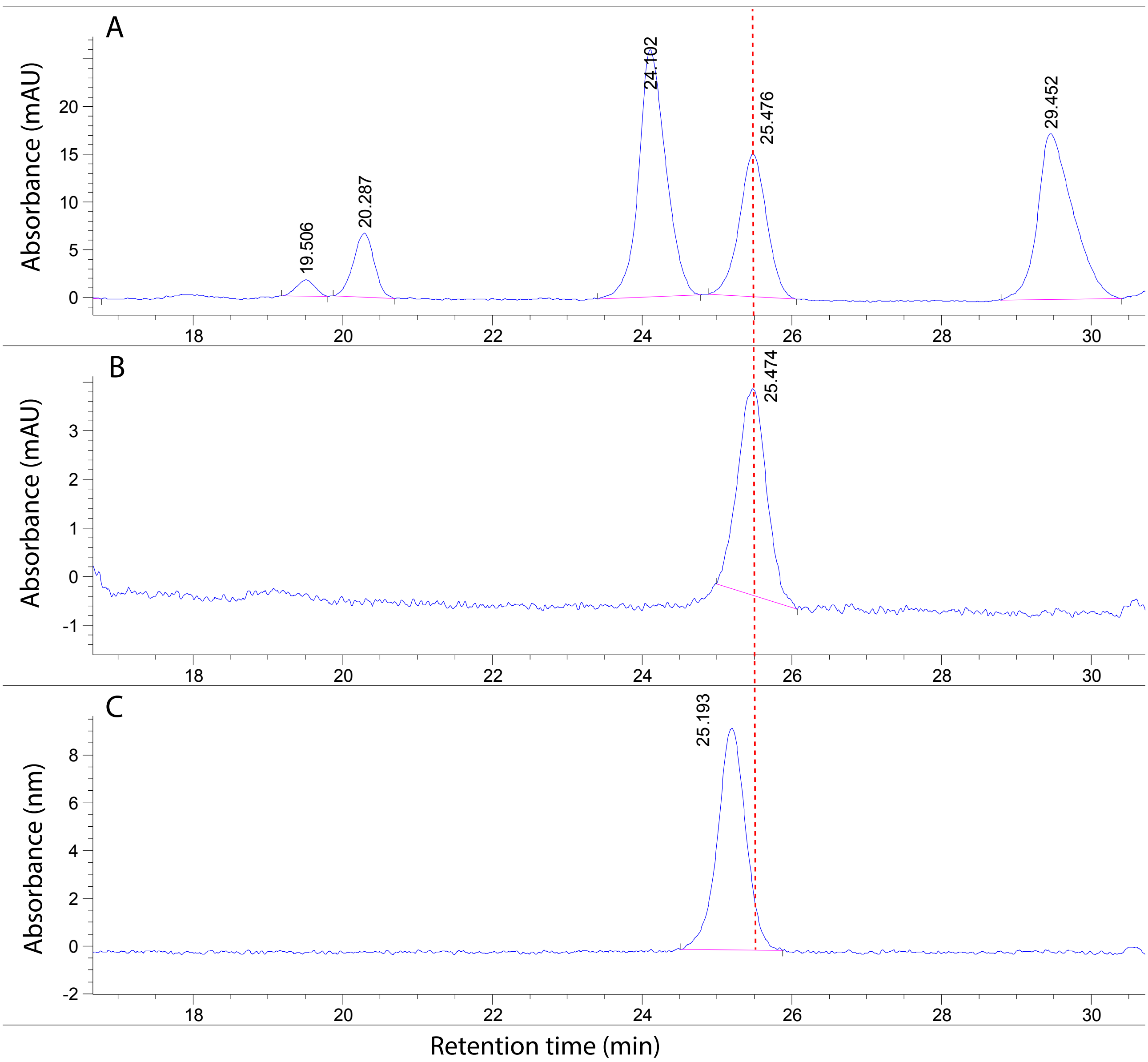

Figure S41. (A) HPLC-DAD (340 nm) chromatogram showing (A) L-FDAA derivatives of partial hydrolysate of **2**, and authentic standards of (B) L-FDAA-D-*allo*-Ile-D-Ala and (C) L-FDAA-D-*allo*-Ile-L-Ala

4. MSMS analysis of talarolides

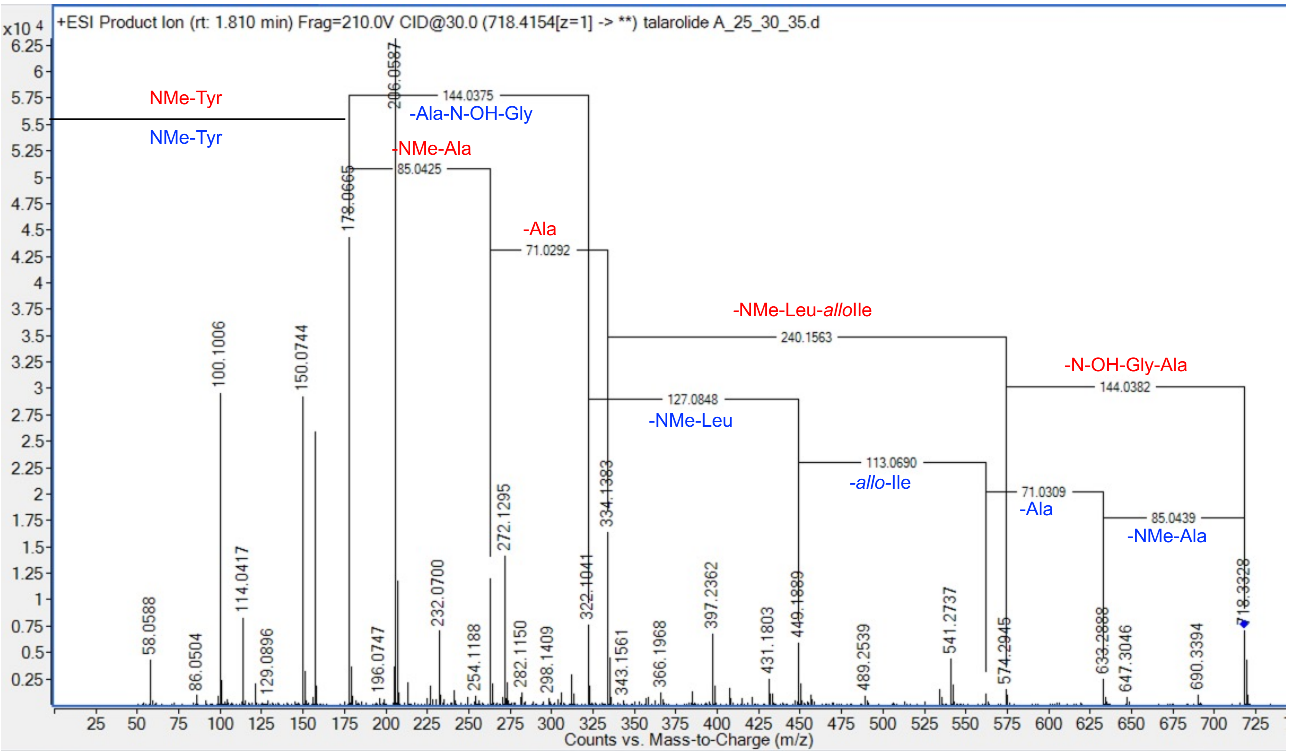

Figure S42. UPLC-QTOF-MS/MS spectrum of talarolide A (**1**) at 30 V colision energy with two different diagnostic fragmentations (in red and blue)

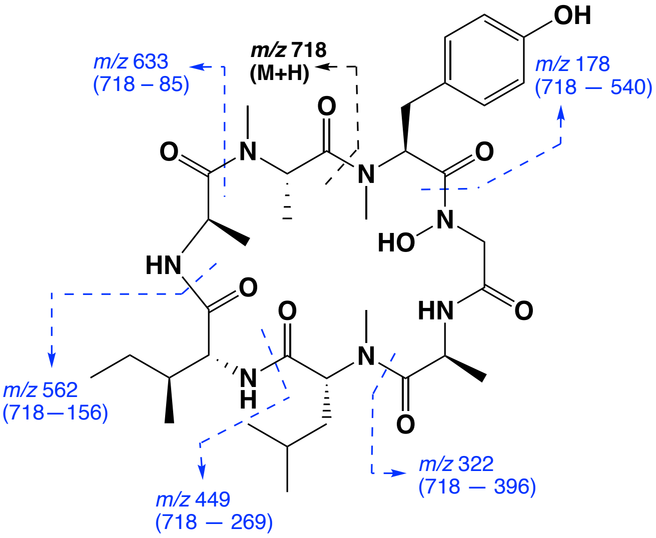

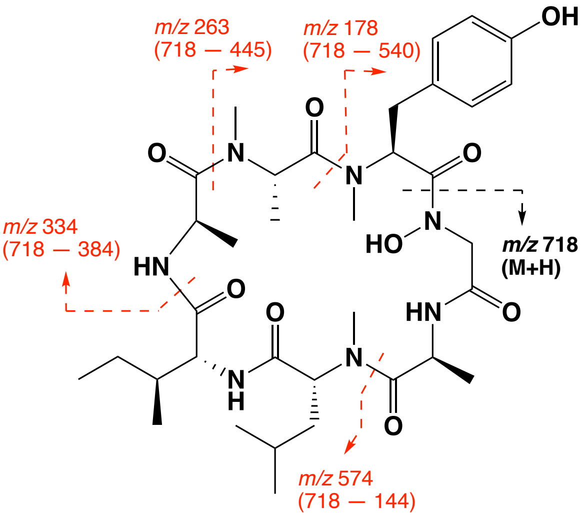

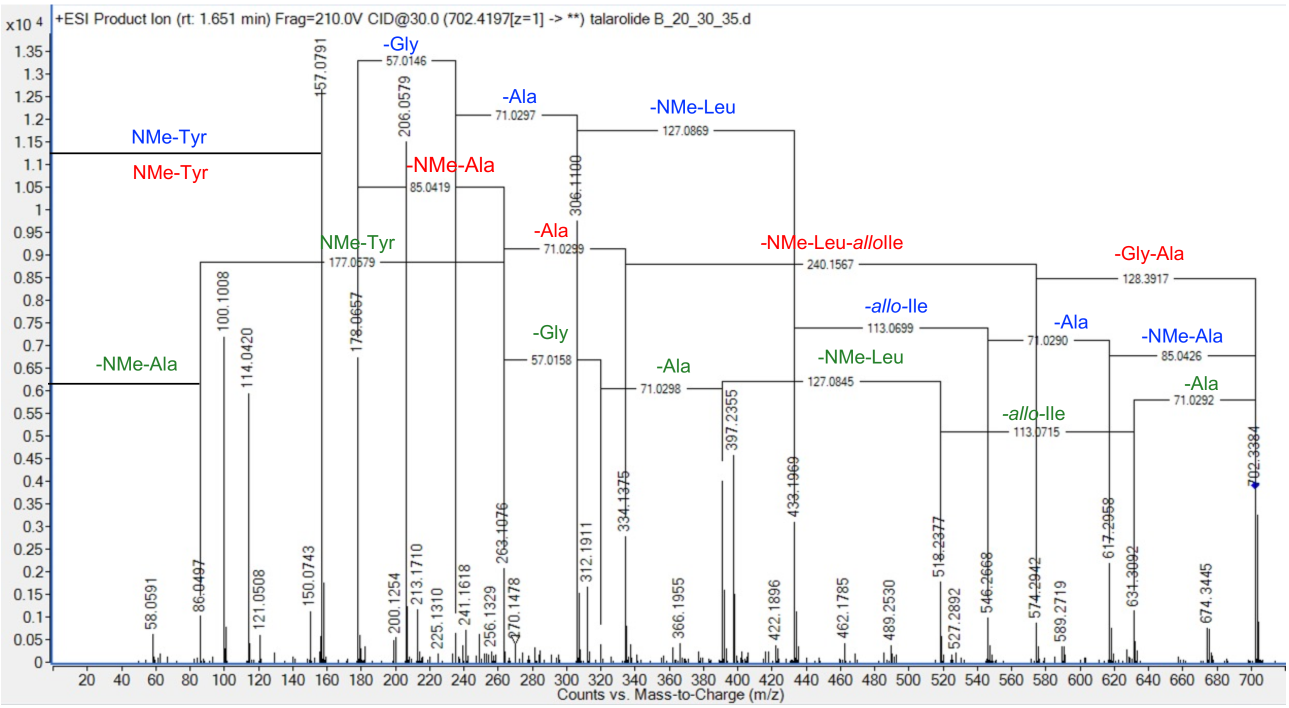

Figure S43. UPLC-QTOF-MS/MS spectrum of talarolide B (**2**) at 30 V colision energy with three different diagnostic fragmentations (in red, blue and green)

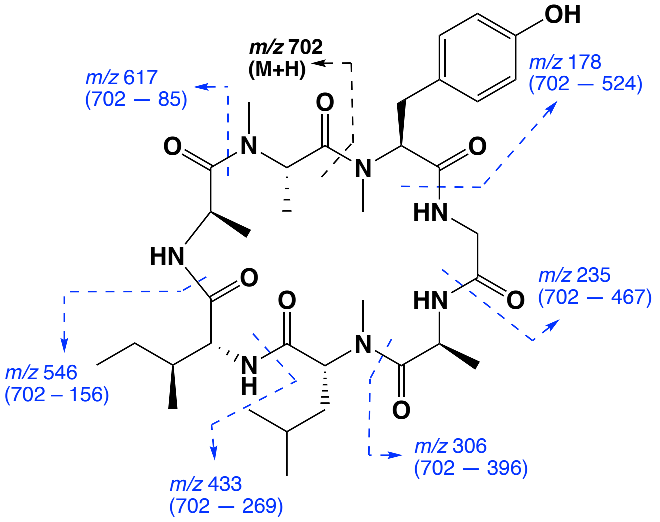

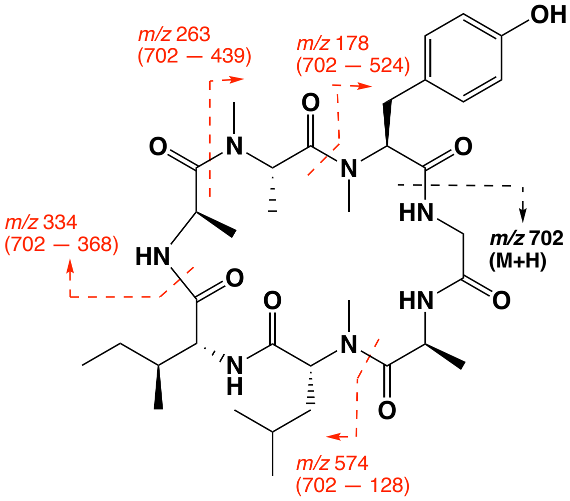

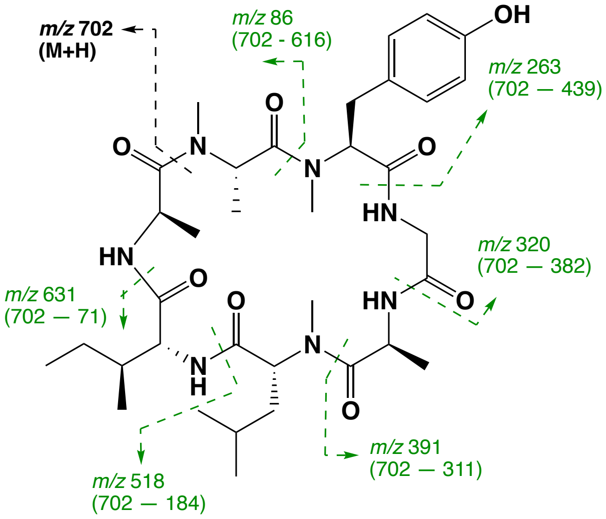

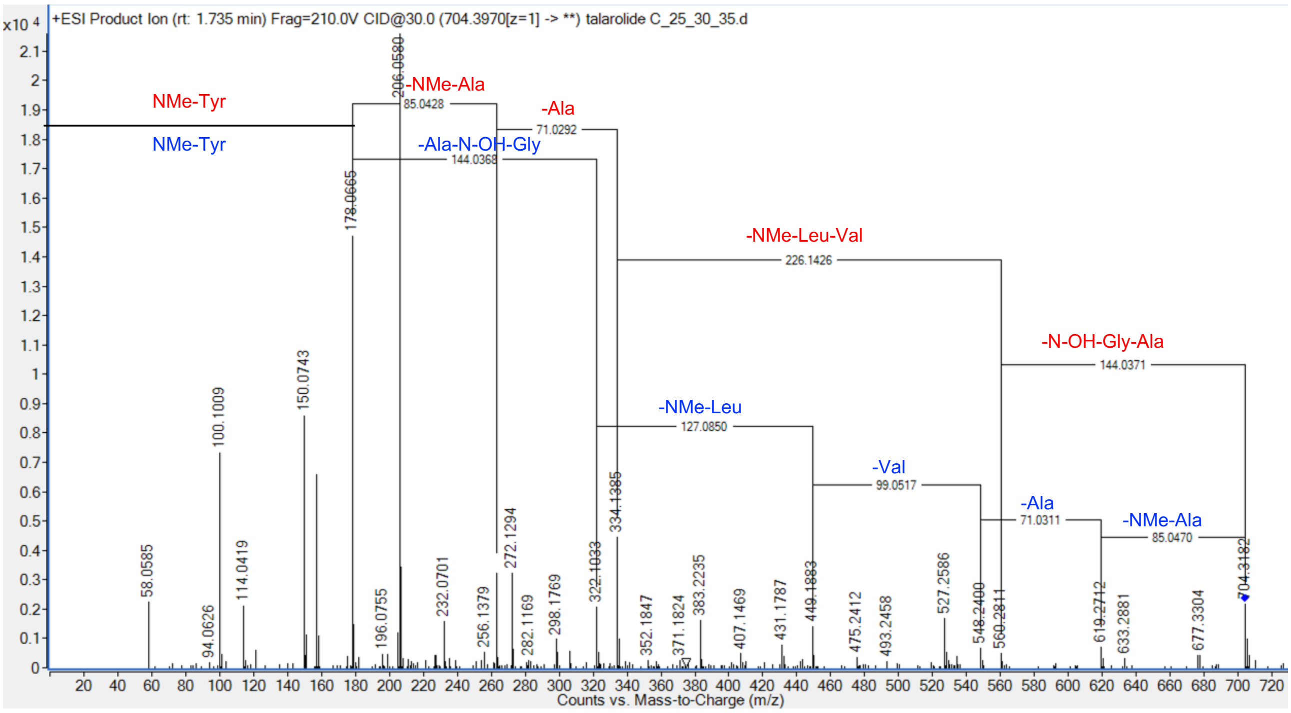

Figure S44. UPLC-QTOF-MS/MS spectrum of talarolide C (**3**) at 30 V colision energy with two different diagnostic fragmentations (in red and blue)

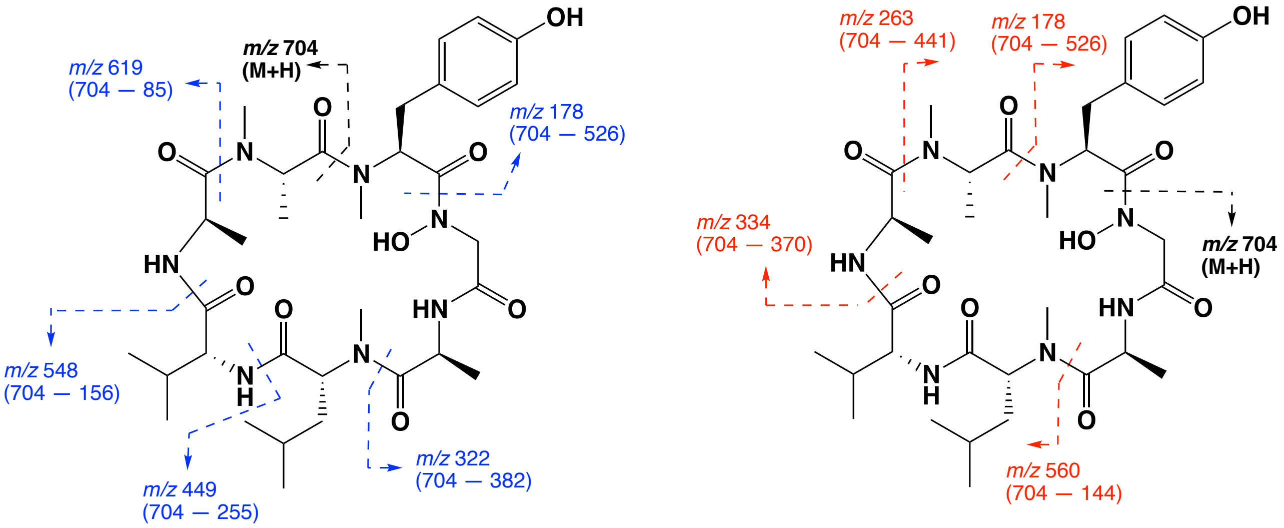

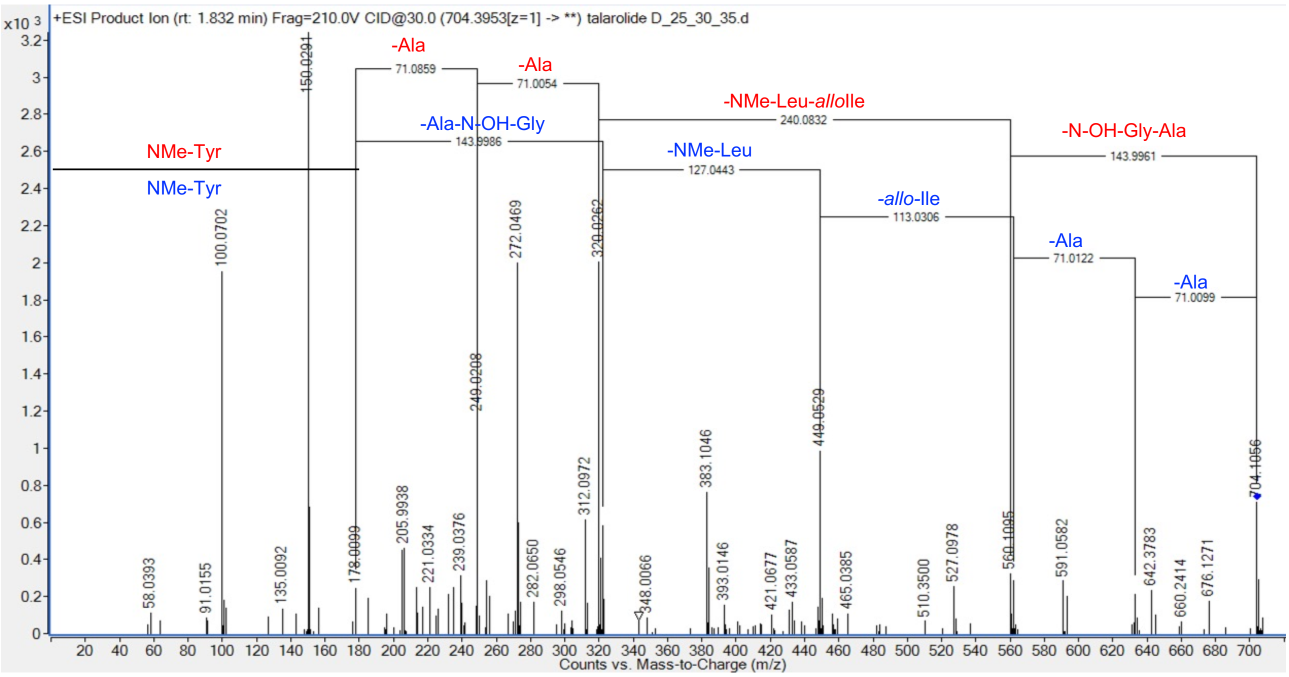

Figure S45. UPLC-QTOF-MS/MS spectrum of talarolide D (**4**) at 30 V colision energy with two different diagnostic fragmentations (in red and blue)

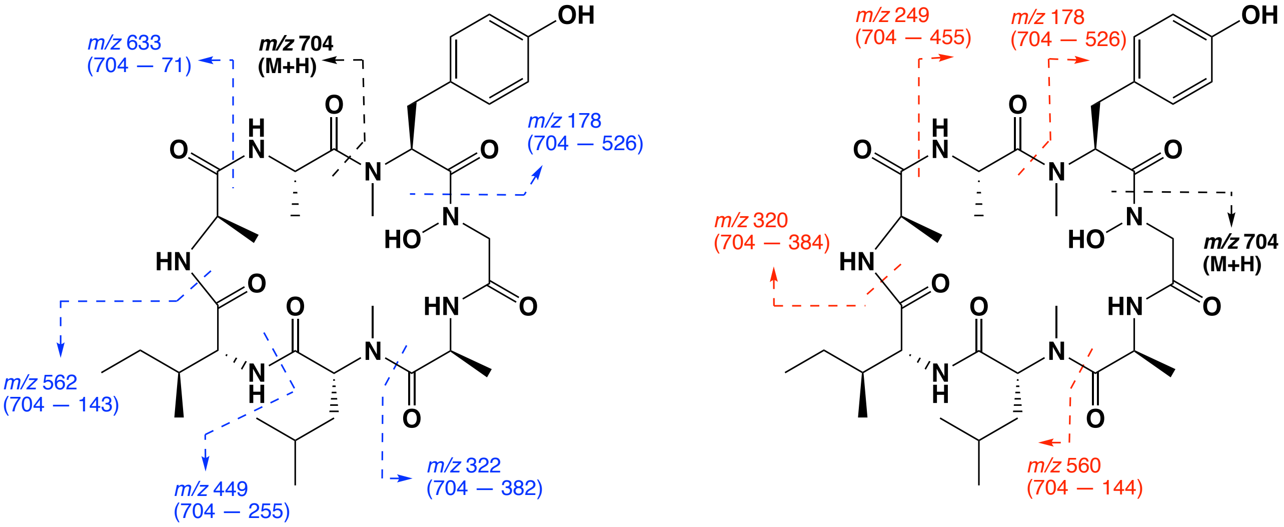

5. Synthetic dipeptides and synthetic talarolide B

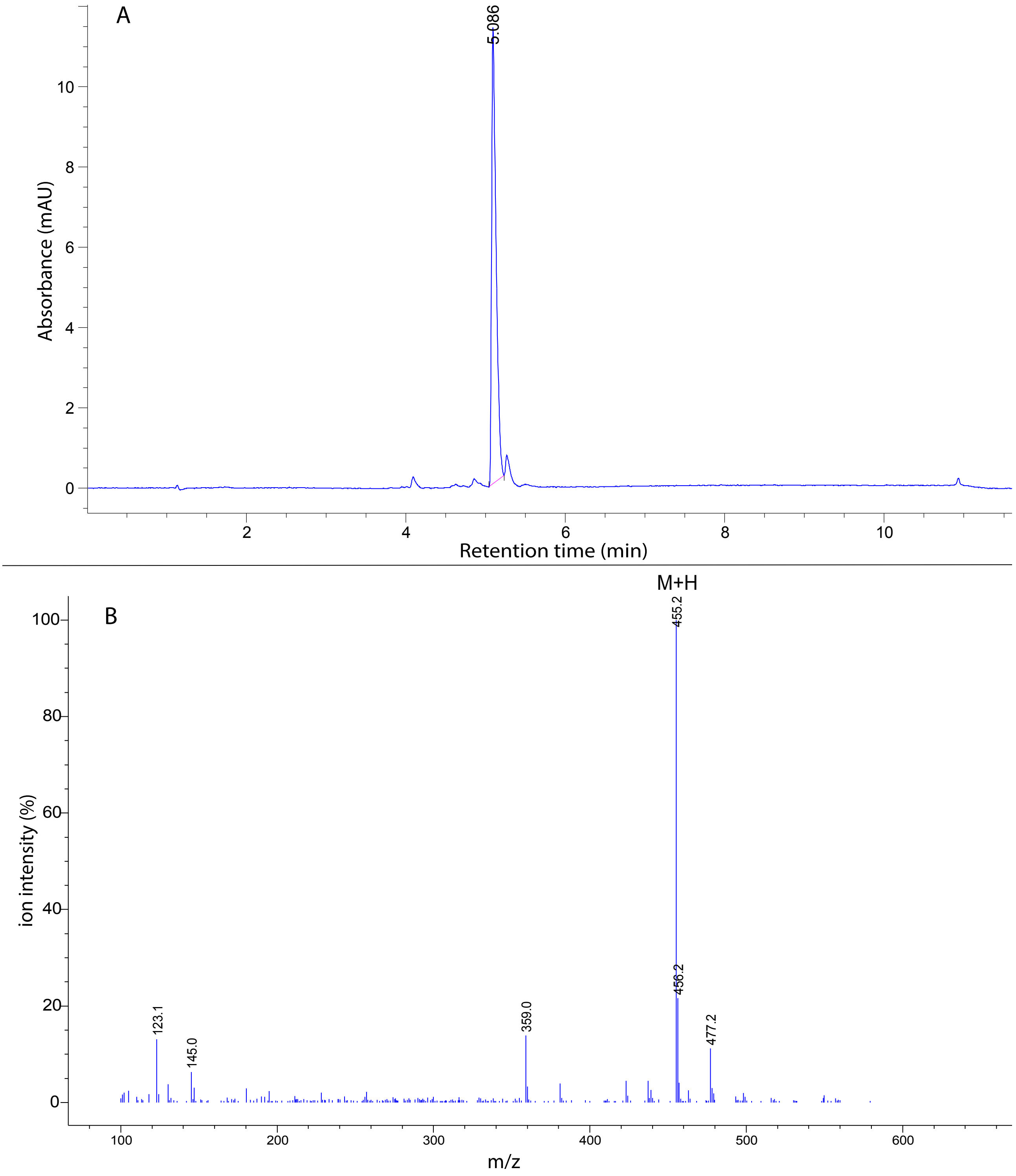

Figure S46. HPLC-DAD-MS of synthetic L-FDAA-D-*allo*-Ile-D-Ala; (A) HPLC-DAD (340 nm) chromatogram and (B) LRMS(+) spectrum of the peak at 5.086 min.

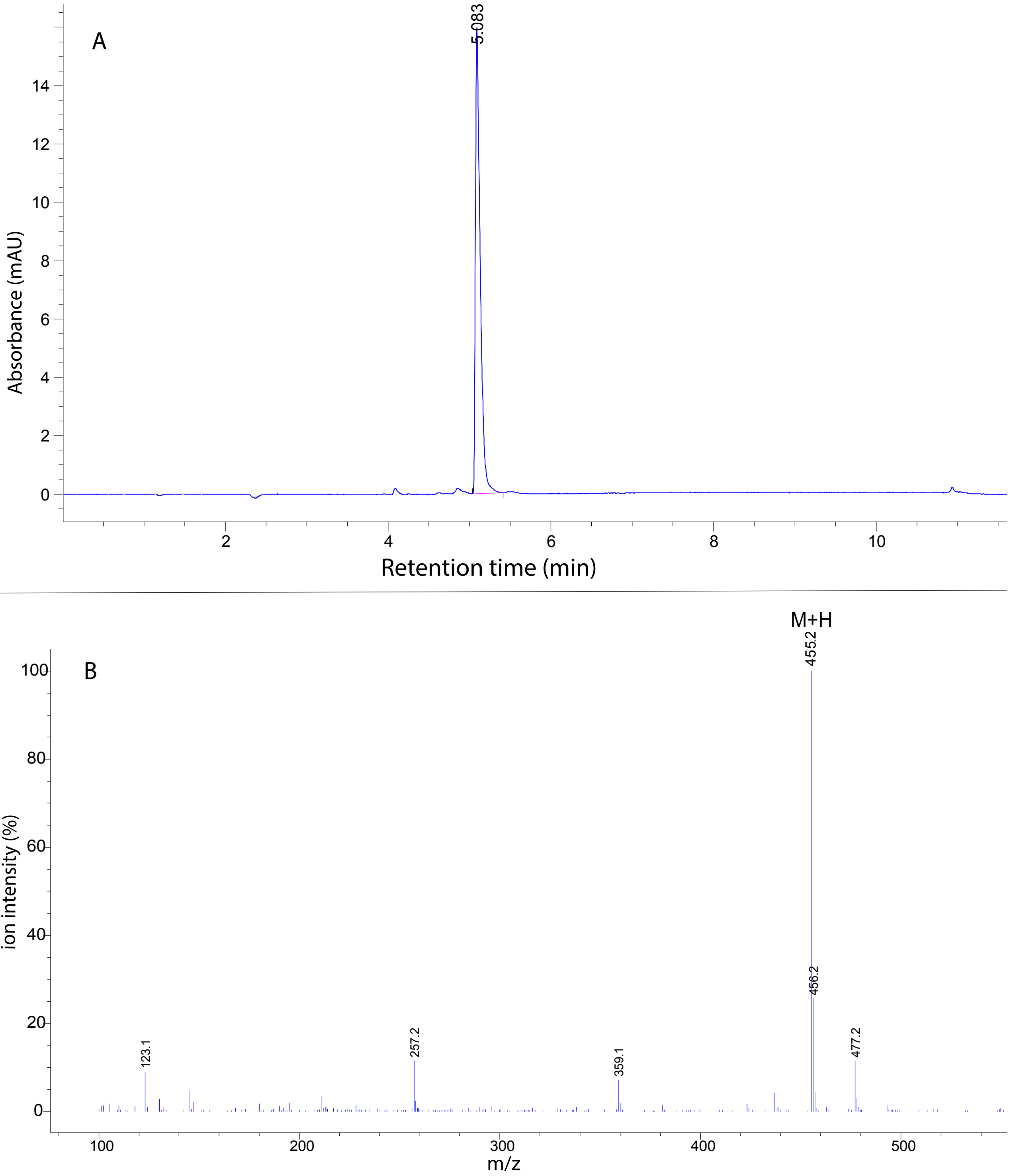

Figure S47. HPLC-DAD-MS of synthetic L-FDAA-D-*allo*-Ile-L-Ala; (A) HPLC-DAD (340 nm) chromatogram and (B) LRMS(+) spectrum of the peak at 5.083 min.

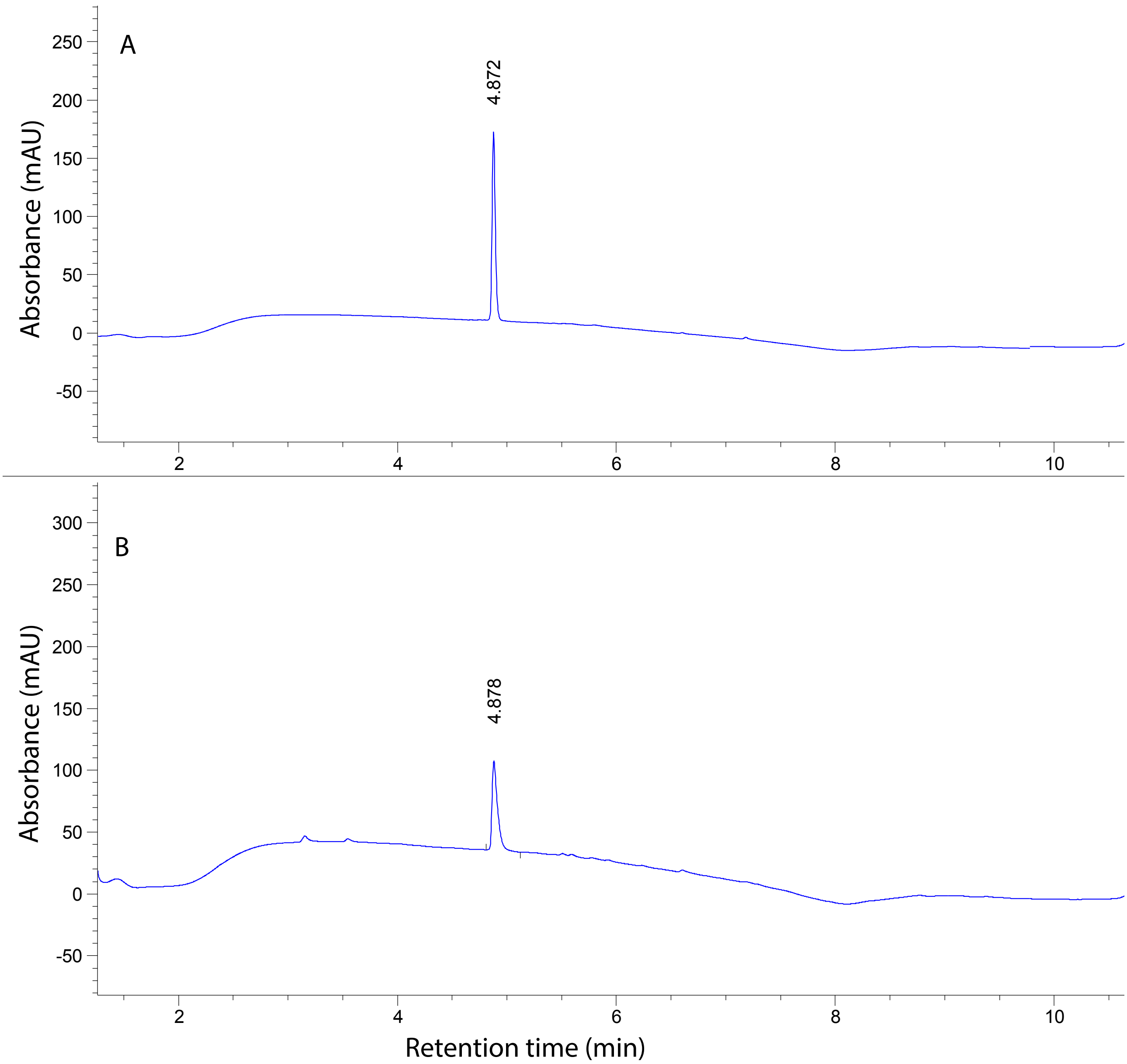

Figure S48. HPLC-DAD (210 nm) chromatograms of (A) synthetic **2** and (B) natural product **2**

Mass Spectrum Molecular Formula Report

Analysis Info

Analysis Name	D:\Data\Waleed\Talarolide-B 000001.d	Acquisition Date	6/6/2023 11:07:44 AM
Method	tune-med_AP.m	Operator	a.salim
Sample Name	Talarolide-B	Instrument / Ser#	micrOTOF 213750.00232
Comment			

Acquisition Parameter

Source Type	ESI	Ion Polarity	Positive	Set Nebulizer	0.8 Bar
Focus	Not active			Set Dry Heater	180 °C
Scan Begin	100 m/z	Set Capillary	4500 V	Set Dry Gas	5.0 l/min
Scan End	1000 m/z	Set End Plate Offset	-500 V	Set Divert Valve	Source

Generate Molecular Formula Parameter

Formula, min.		
Formula, max.		
Measured m/z	Tolerance	Charge
Check Valence	Minimum	Maximum
Nirogen Rule	Electron Configuration	
Filter H/C Ratio	Minimum	Maximum
Estimate Carbon		

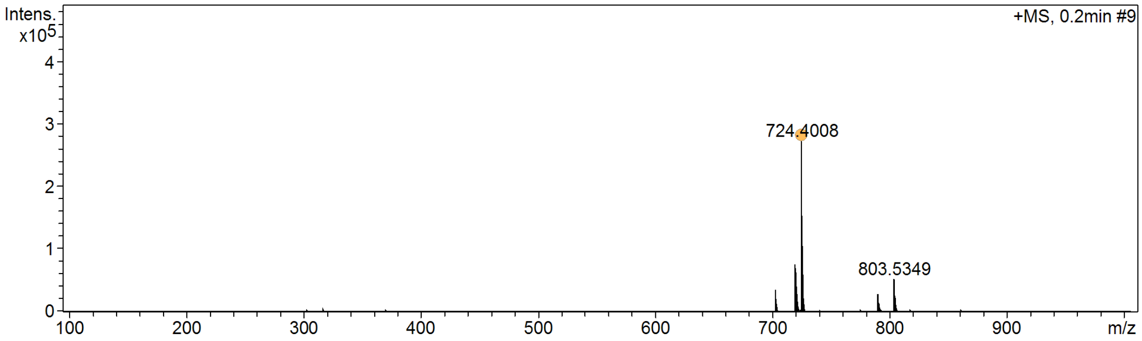

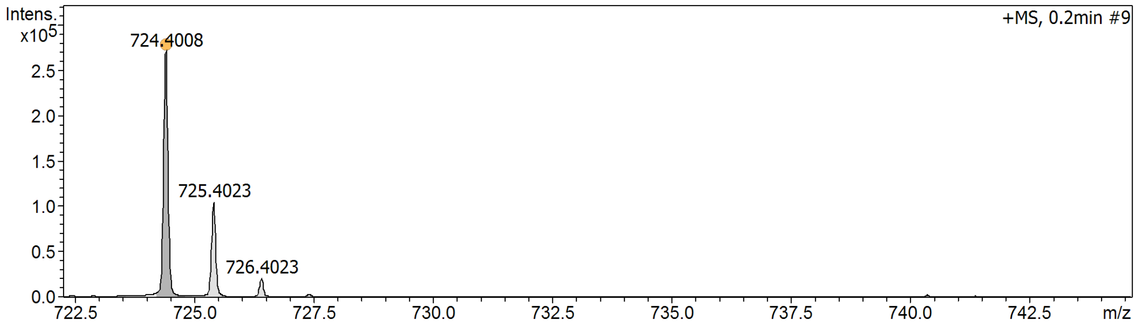

Meas. m/z	#	Ion Formula	m/z	err [ppm]	mSigma	# Sigma	Score	rdb	e⁻ Conf	N-Rule
724.4008	1	C35H55N7NaO8	724.4004	-0.4	17.3	1	100.00	11.5	even	ok

Figure S49. HRMS data for synthetic talarolide B (**2**)

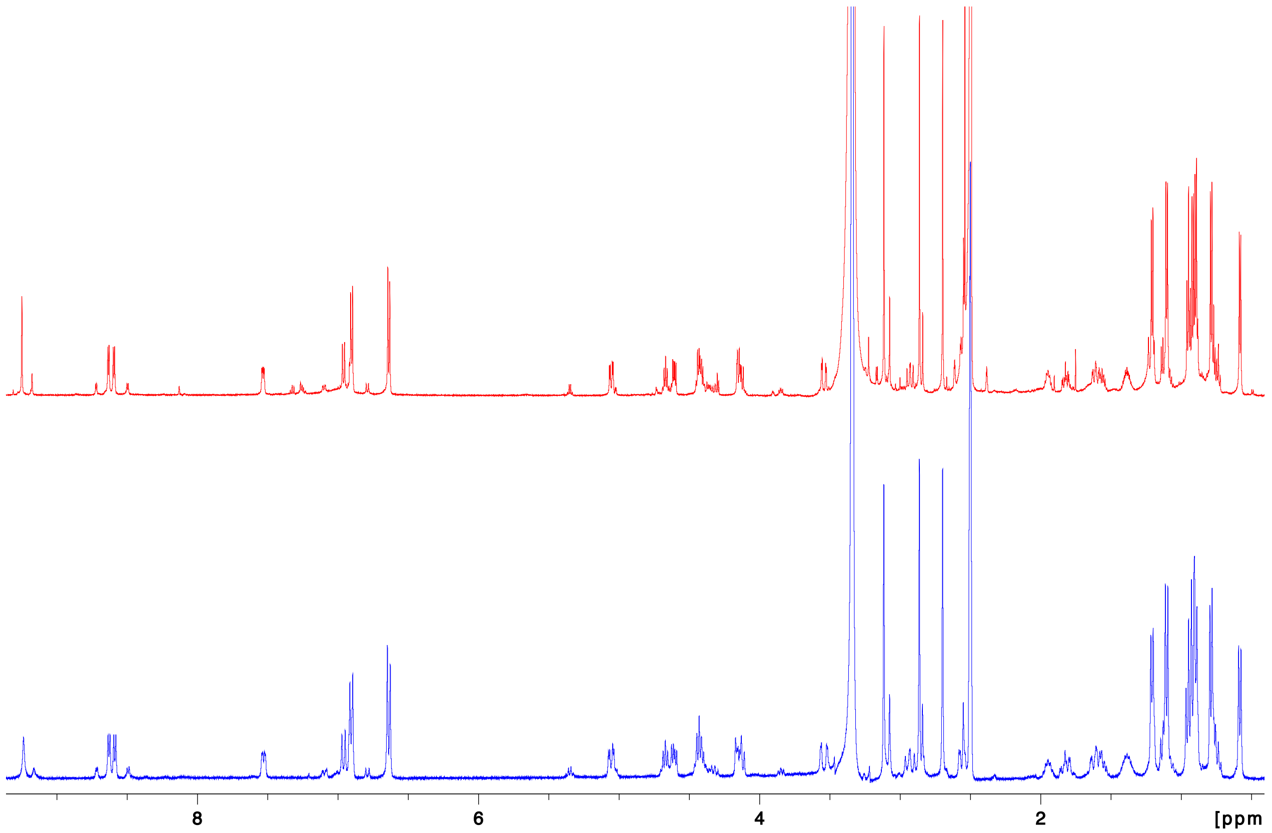

Figure S50. ^{1}H NMR(600 MHz, DMSO-d_6) spectra of synthetic (red) and natural (blue) talarolide B (**2**)

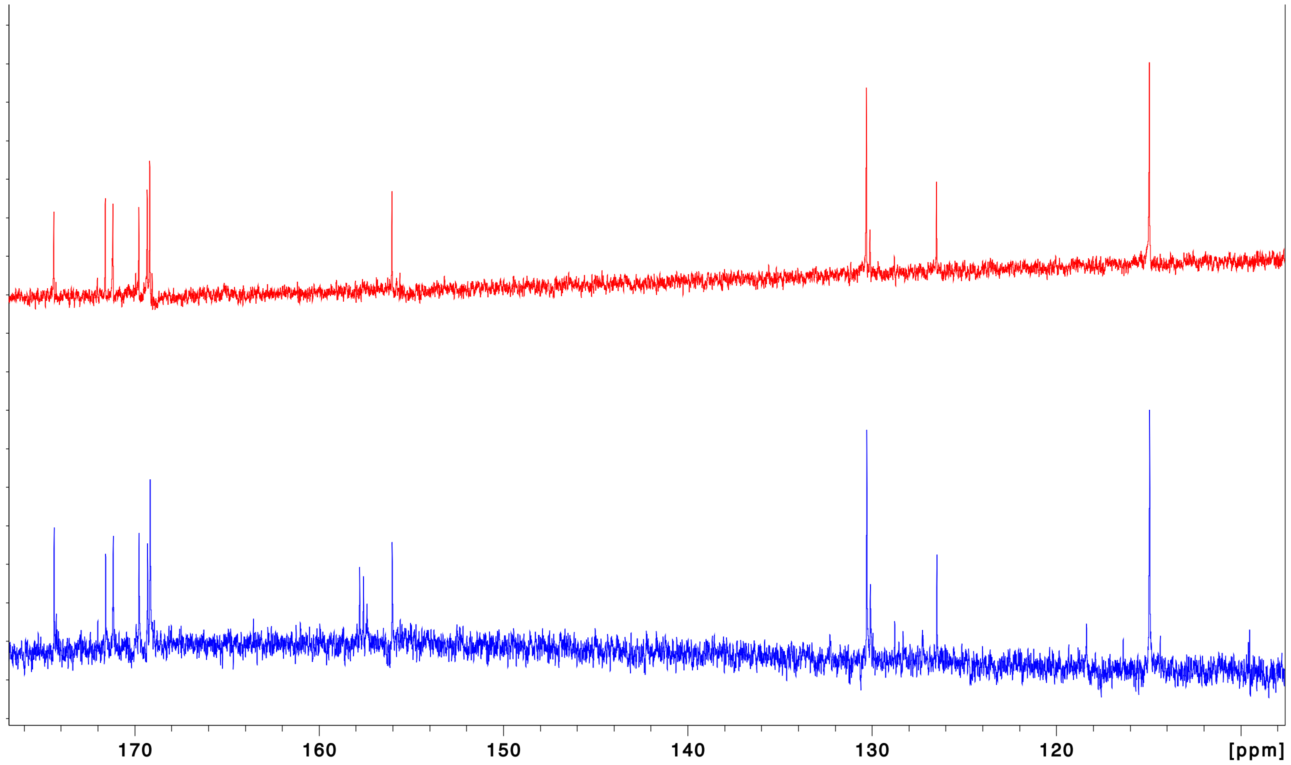

Figure S51. ^{13}C NMR(150 MHz, DMSO-d_6) spectra of synthetic (red) and natural (blue) talarolide B (**2**)

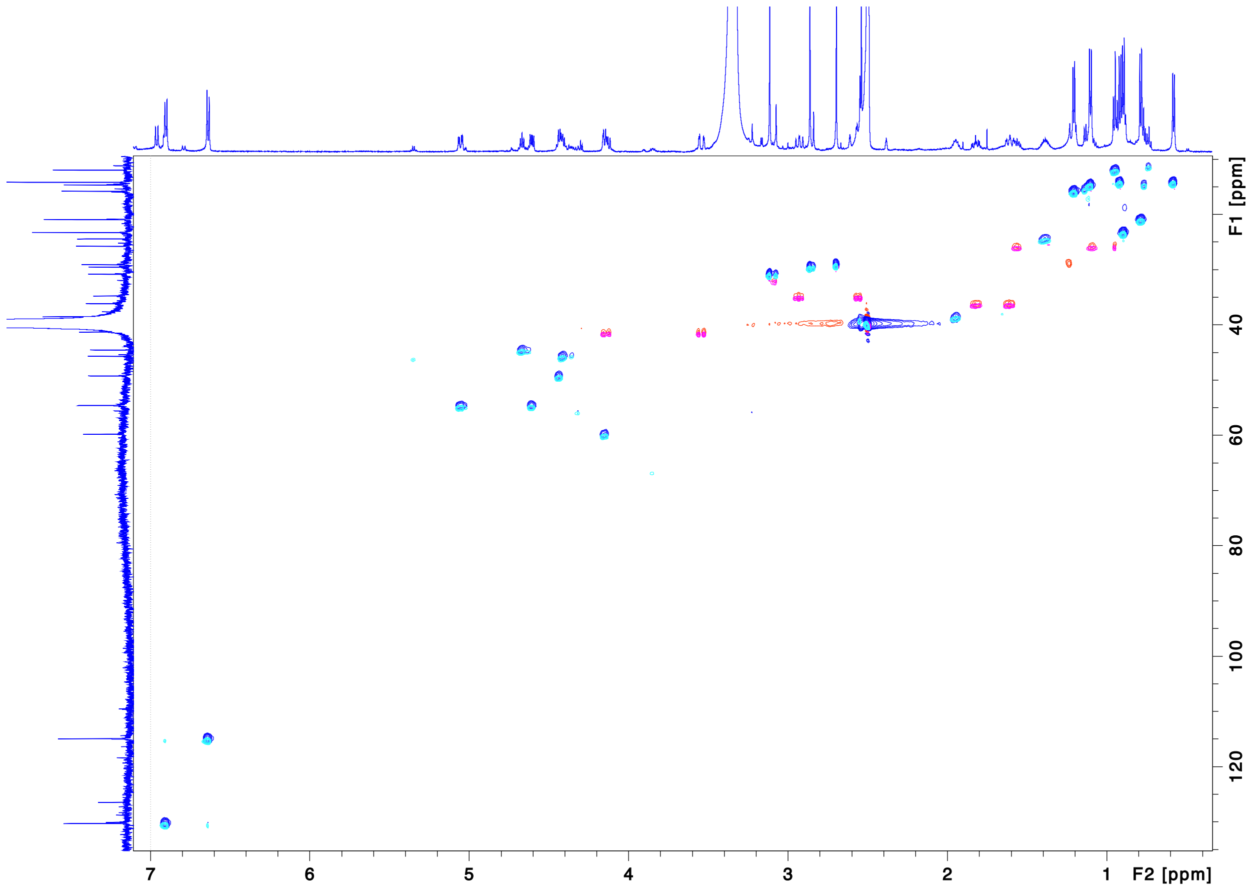

Figure S52. Overlay of HSQC (DMSO-d_6) spectra of synthetic (cyan and magenta peaks) and natural (blue and red peaks) talarolide B (**2**)

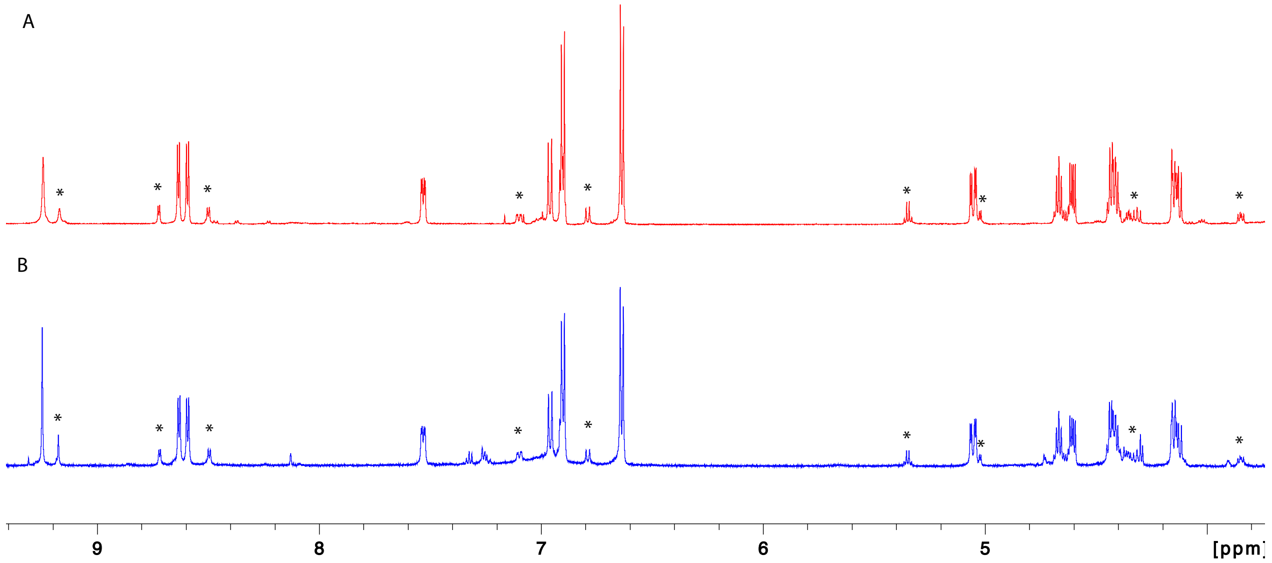

Figure S53. ^{1}H NMR (DMSO-d_6) spectra of synthetic (red) and natural (blue) talarolide B (**2**) showing the signals of the minor conformers (marked with *)

6. ROE and distance restraints for talarolide A in DMSO-d_6

(N-OH)Gly1
assi(resi 1 and name HA1)(resi 1 and name HO) 0.0 0.0 5.0 ! (6)w
assi(resi 1 and name HA1)(resi 2 and name HN) 0.0 0.0 3.5 ! (10)M
assi(resi 1 and name HA1)(resi 1 and name HA2) 0.0 0.0 2.7 ! (16)S
assi(resi 1 and name HA2)(resi 2 and name HN) 0.0 0.0 5.0 ! (7)W
assi(resi 1 and name HO)(resi 6 and name HI*) 0.0 0.0 6.0 ! (6)w + correction
assi(resi 1 and name HO)(resi 4 and name HN) 0.0 0.0 6.0 ! (1)Vw
assi(resi 1 and name HO)(resi 4 and name HG1*) 0.0 0.0 6.0 ! (5)w

Ala2
assi(resi 2 and name HA)(resi 2 and name HN) 0.0 0.0 3.5 ! (8)M
assi(resi 2 and name HA)(resi 3 and name HI*) 0.0 0.0 5.0 ! (10)M + 1.5 A
correction
assi(resi 2 and name HA)(resi 2 and name HB*) 0.0 0.0 4.2 ! (13)S + 1.5 A
correction
assi(resi 2 and name HA)(resi 4 and name HN) 0.0 0.0 6.0 ! (2)vw

Leu3(N-CH3)
assi(resi 3 and name HA)(resi 4 and name HN) 0.0 0.0 5.0 ! (7)w
assi(resi 3 and name HA)(resi 3 and name HB2) 0.0 0.0 3.5 ! (10)M
assi(resi 3 and name HA)(resi 3 and name HG) 0.0 0.0 5.0 ! (6)w
assi(resi 3 and name HA)(resi 3 and name HD1) 0.0 0.0 3.5 ! (11)M
assi(resi 3 and name HA)(resi 3 and name HD2) 0.0 0.0 5.0 ! (7)W
assi(resi 3 and name HI*)(resi 4 and name HG1*) 0.0 0.0 6.0 ! (9)M
assi(resi 3 and name HI*)(resi 2 and name HB*) 0.0 0.0 6.0 ! (6)W

allo-Ile4
assi(resi 4 and name HA)(resi 4 and name HN) 0.0 0.0 6.0 ! (8) M
assi(resi 4 and name HA)(resi 5 and name HN) 0.0 0.0 3.5 ! (11) M
assi(resi 4 and name HA)(resi 4 and name HG*) 0.0 0.0 6.0 ! (11) M + correction
assi(resi 4 and name HN)(resi 3 and name HB*) 0.0 0.0 6.0 ! (3) VW

Ala5
assi(resi 5 and name HA)(resi 1 and name HO) 0.0 0.0 5.5 ! (6)W
assi(resi 5 and name HA)(resi 6 and name HI*) 0.0 0.0 4.2 ! (12)S
assi(resi 5 and name HA)(resi 5 and name HB*) 0.0 0.0 4.2 ! (12)S
assi(resi 5 and name HN)(resi 4 and name HB) 0.0 0.0 4.0 ! (10)M
assi(resi 5 and name HN)(resi 4 and name HG1*) 0.0 0.0 6.0 ! (2)VW

Ala6(N-CH3)
assi(resi 6 and name HA)(resi 7 and name HD*) 0.0 0.0 4.5 ! (9)M
assi(resi 6 and name HA)(resi 6 and name HB*) 0.0 0.0 4.2 ! (14)S + 1.5 A
correction
assi(resi 6 and name HI*)(resi 6 and name HB*) 0.0 0.0 6.0 ! (9)M + correction
assi(resi 6 and name HI*)(resi 5 and name HB*) 0.0 0.0 6.0 ! (9)M + correction
assi(resi 6 and name HA)(resi 7 and name HE*) 0.0 0.0 6.0 ! (4)W

Tyr7(N-CH3)
assi(resi 7 and name HA)(resi 1 and name HO) 0.0 0.0 3.5 ! (8)M

```
assi(resi 7  and name  HA  )(resi  7  and name  HD* )      0.0 0.0     4.5       ! (8)M
assi(resi 7  and name  HA  )(resi  4  and name  HG1* )    0.0 0.0     6.0       ! (8)M
assi(resi 7  and name  HD*  )(resi  4  and name  HG1* )  0.0 0.0     7.0       ! (7)W
assi(resi 7  and name  HE*  )(resi  6  and name  HB* )     0.0 0.0     6.0       ! (6)W
assi(resi 7  and name  HD*  )(resi  7  and name  HB* )    0.0 0.0     6.0       ! (9,10)W
assi(resi 7  and name  HD*  )(resi  7  and name  HI* )     0.0 0.0     6.0       ! (9)M
assi(resi 7  and name  HA  )(resi  7  and name  HI* )      0.0 0.0     6.0       ! (6)W
assi(resi 7  and name  HA  )(resi  6  and name  HA )       0.0 0.0     3.5       ! (14)S
```

Hydrogen Bond restraints

```
assign (resid  5 and name  O     )(resid 1 and name  HO   ) 1.88  0.3 0.42
assign (resid  5 and name  O     )(resid 1 and name  ON    ) 2.88 0.3 0.3
```

Dihedral Angle restraints

Ala2 ^{3}J = 5.3 Hz

```
  assign (resid   1 and name C )   (resid 2 and name N )
          (resid   2 and name CA)   (resid 2 and name C )   1.0  -60.0   30.0  2
```

Ile4 ^{3}J = 9.6 Hz

```
  assign (resid   3 and name C )   (resid 4 and name N )
          (resid   4 and name CA)   (resid 4 and name C )   1.0  -100.0  30.0  2
```

ala5 ^{3}J = 4.3 Hz

```
  assign (resid   4 and name C )   (resid 5 and name N )
          (resid   5 and name CA)   (resid 5 and name C )   1.0   60.0   30.0   2
```
